# Mind the gap: covariate constrained randomisation can protect against substantial power loss in parallel cluster randomised trials

**DOI:** 10.1186/s12874-022-01588-8

**Published:** 2022-04-13

**Authors:** Caroline Kristunas, Michael Grayling, Laura J. Gray, Karla Hemming

**Affiliations:** 1 Department of Health Sciences, University of Leicester, Leicester, UK; 2 Institute of Clinical Sciences, University of Birmingham, Birmingham, UK; 3 Population Health Sciences Institute, Newcastle University, Newcastle upon Tyne, UK; 4 Institute of Applied Health Research, University of Birmingham, Birmingham, UK

**Keywords:** Group-randomised trial, Restricted randomisation, Candidate set size, Covariate adjusted analysis, Small sample

## Abstract

**Background:**

Cluster randomised trials often randomise a small number of units, putting them at risk of poor balance of covariates across treatment arms. Covariate constrained randomisation aims to reduce this risk by removing the worst balanced allocations from consideration. This is known to provide only a small gain in power over that averaged under simple randomisation and is likely influenced by the number and prognostic effect of the covariates.

We investigated the performance of covariate constrained randomisation in comparison to the worst balanced allocations, and considered the impact on the power of the prognostic effect and number of covariates adjusted for in the analysis.

**Methods:**

Using simulation, we examined the Monte Carlo type I error rate and power of cross-sectional, two-arm parallel cluster-randomised trials with a continuous outcome and four binary cluster-level covariates, using either simple or covariate constrained randomisation. Data were analysed using a small sample corrected linear mixed-effects model, adjusted for some or all of the binary covariates. We varied the number of clusters, intra-cluster correlation, number and prognostic effect of covariates balanced in the randomisation and adjusted in the analysis, and the size of the candidate set from which the allocation was selected. For each scenario, 20,000 simulations were conducted.

**Results:**

When compared to the worst balanced allocations, covariate constrained randomisation with an adjusted analysis provided gains in power of up to 20 percentage points. Even with analysis-based adjustment for those covariates balanced in the randomisation, the type I error rate was not maintained when the intracluster correlation is very small (0.001). Generally, greater power was achieved when more prognostic covariates are restricted in the randomisation and as the size of the candidate set decreases. However, adjustment for weakly prognostic covariates lead to a loss in power of up to 20 percentage points.

**Conclusions:**

When compared to the worst balanced allocations, covariate constrained randomisation provides moderate to substantial improvements in power. However, the prognostic effect of the covariates should be carefully considered when selecting them for inclusion in the randomisation.

**Supplementary Information:**

The online version contains supplementary material available at 10.1186/s12874-022-01588-8.

## Background

Cluster randomised trials (CRTs) typically randomise a relatively small number of clusters [1, 2]. The randomisation of a small number of units is known to increase the possibility of chance imbalances across intervention and control arms, which can lead to a reduction in study power, as well as potentially undermining the validity of the findings [3–5]. Restricted randomisation is advocated as a method to prevent this chance of imbalance [6].

Historically, restricted randomisation methods for CRTs included either stratified randomisation or matched pair designs [7, 8]. Matched pair designs have mostly been viewed with scepticism [6, 9], but stratification has become widely adopted [2, 10]. Yet, implementing stratification in trials with a small number of clusters and more than a couple of strata can lead to sparse strata which cannot be balanced across study arms [11, 12]. The matched pair design has been shown to be more efficient than stratification when there are more than 10 clusters per arm and the matching is strong, but otherwise there is little gain from either stratification or pair matching over simple randomisation [13]. Consequently, in CRTs, randomisation methods such as minimisation and covariate constrained randomisation that focus on the global balance - as opposed to balance on discrete characteristics – have become more popular [14].

Under covariate constrained randomisation, the pool of possible randomisation schemes (randomisation space) is restricted to exclude those schemes with the worst balance of the covariates of interest. This is determined by using a balance metric to score the balance of each scheme with respect to the covariates [15, 16]. A randomisation scheme is then selected at random from the restricted randomisation space, known as the candidate set.

Covariate constrained randomisation often provides a better balance than other restricted randomisation techniques [16]. In addition, it has been shown to lead to a small increase in power compared to simple randomisation, whilst maintaining the type I error rate provided that all covariates used in the design are adjusted in the analysis [15, 17, 18]. However, despite the appeal of covariate constrained randomisation, it is relatively rarely used, being adopted in only around 10% of CRTs [2]. This could be due to the general slow uptake of novel methods, but the existence of packages in SAS, Stata and R that allow covariate constrained randomisation to be implemented [19–21], should have encouraged some use. The lack of uptake might instead be explained by researchers questioning whether the complexity of implementing this method is justified, when the gains in power are relatively small, in comparison to covariate adjustment alone. For example, in their evaluations, Li et al. found that covariate constrained randomisation often resulted in only a small increase in power, compared to simple randomisation with comparable covariate adjustment [17, 18]. Researchers may therefore choose not to balance some prognostic covariates, so that they are able to implement a simpler method of randomisation, such as stratification or simple randomisation.

However, to date, power gains have been compared to that averaged over all possible allocations under simple randomisation [22]. Yet, in any given CRT the range of power that can be achieved under different allocations can vary considerably depending on the number and size of the clusters and the intra-cluster correlation [23, 24], and it is possible that the allocation selected under simple randomisation might be that with the lowest power. We suggest that rather than compare to the power averaged over all simple randomisations, a more useful metric is to compare to those allocations that represent the “worst” possible set of allocations (i.e., the bottom 10^th^ percentile of the allocations).

Since balancing covariates in the design requires adjustment for those covariates in the analysis in order to maintain the type I error rate, consideration of the prognostic effect of the covariates is also needed [17, 18]. In individually randomised trials with continuous outcomes, adjusting for prognostic covariates increases precision, and adjusting for non-prognostic covariates does not detrimentally affect precision [25]. However, when the outcome is binary and the sample size small, adjustment for covariates can lead to an inflated type I error rate [25–27]. For CRTs, the impact of the magnitude of the prognostic effect and number of covariates adjusted in the analysis is unclear.

### Objectives

Our primary objective was to consider the performance of covariate constrained randomisation compared to the allocations with the worst possible balance. Under this comparison we confirm previous findings of (i) the necessity to adjust the analysis for all of the covariates balanced in the randomisation, in order to maintain the type I error rate; (ii) the gain in power from balancing more covariates and decreasing the candidate set size; and (iii) the gain in power obtained under covariate constrained randomisation over simple randomisation with covariate adjustment. Our secondary objective, was to consider the impact of the prognostic effect and number of covariates on the power of an adjusted analysis under simple randomisation. We limited our considerations to equal allocations, cross-sectional designs, continuous outcomes and binary cluster-level covariates.

### Motivating example

We illustrate the use of covariate constrained randomisation using data adapted from a planned CRT. Ten emergency departments (the clusters) will be randomised 1:1 to an acute mental healthcare bundle, or to standard emergency department healthcare. Regardless of their size, it is expected that each emergency department will contribute around 300 patients over the duration of the trial. Two possible primary outcomes are being considered: a clinical outcome that is quite rare and likely to have an intra-cluster correlation of around 0.001; and a process-type outcome with a likely intra-cluster correlation of 0.1.

Three potential binary cluster-level confounders have been identified: (i) annual patient volume (classified as small or large); (ii) existence of a dedicated mental health team; and (iii) direct access to urgent mental health follow-up appointments. The values of these potential confounders for each emergency department are shown in Table 1, alongside the strata that would be formed if a stratified randomisation was used. Randomising using stratification would result in eight (= 2*2*2) sparse stratum, with only two strata comprising more than one emergency department, making it unlikely that a balanced allocation will be achieved (Table 1). A forth binary cluster-level confounder was also being considered, the use of a formal guideline, policy or tool for mental healthcare, but limited information was available on this at the time of randomisation. Further information could be obtained on this confounder during the trial and adjusted for in the analysis. Covariate constrained randomisation is considered to be an appealing method for this trial, as it may allow all confounders to be balanced in the randomisation and improve the power.

**Table 1:** Illustrative case study randomising ten emergency departments (clusters) with three cluster-level binary covariates

	**Emergency department (cluster)**									
**Characteristic**	1	2	3	4	5	6	7	8	9	10
Large patient volume	Yes	No	Yes	Yes	No	Yes	No	No	No	No
Dedicated mental health team	Yes	No	Yes	Yes	Yes	No	No	No	No	Yes
Access to urgent mental health follow-up	Yes	No	No	No	Yes	Yes	Yes	No	No	No
	**Strata**									
	1	2	3	4	5	6	7	8		
Large patient volume	Yes	Yes	Yes	Yes	No	No	No	No		
Dedicated mental health team	Yes	Yes	No	No	Yes	Yes	No	No		
Access to urgent mental health follow-up	Yes	No	Yes	No	Yes	No	Yes	No		
Number of clusters	1	2	1	0	1	1	1	3		

The researchers seek guidance on how to conduct the randomisation for their trial, particularly whether the benefits of using covariate constrained randomisation are likely to outweigh the additional complexity of using this method over simple randomisation, and whether it would be beneficial to collect information on the fourth confounder for inclusion in the analysis.

## Methods

For our primary objective, a series of simulation studies were conducted based on our motivating example, using a cross-sectional, two-arm parallel CRT, with 1:1 allocation to treatment arms, a continuous outcome and up to four binary cluster-level covariates. In brief, for each simulated trial, covariate constrained randomisation was used to select a randomisation scheme. Given the selected randomisation scheme, individual-level outcome data were generated for each participant under an assumed treatment effect. These data were then analysed using linear mixed models with a small sample correction and the *p*-values stored. For each scenario, this entire process was repeated 20,000 times to determine the Monte Carlo power and type I error rate.

We considered a range of scenarios, increasing the number of clusters, intra-cluster correlation coefficients within the range of the two potential primary outcomes, number of cluster-level covariates balanced in the randomisation and adjusted for in the analysis, and the size of the candidate set.

For our secondary objective, some of the simulation studies for our primary objective were adapted to consider the power and type I error rate under simple randomisation, as the number and size of the prognostic effect of the covariates in the data generation model were varied. Further details are provided at the end of this section.

### Generation of the randomisation scheme

For each scenario, four binary cluster-level covariates were randomly generated from a Bernoulli distribution for each cluster. The allocation of the treatment to clusters was then determined by:

1. Generating the entire randomisation space
2. Scoring the balance of each possible scheme
3. Forming a candidate set of schemes from those schemes with a desired degree of balance
4. Selecting a scheme at random from this candidate set.

For scenarios with a smaller number of clusters, the generated randomisation space consisted of all possible schemes, whereas for larger trials, the randomisation space was restricted to 20,000 schemes (duplicates removed), for computational feasibility.

The balance of each scheme was scored using the “B” balance metric, as considered by Li et al. [17] and proposed by Raab and Butcher [14], which takes the weighted sum of the squared difference in the mean covariate values across the intervention and control arms:

$$B=\sum_{c=1}^{C}{\omega }_{c}{\left({\overline{z} }_{1c}-{\overline{z} }_{0c}\right)}^{2}$$

where, ${\omega }_{c}$ is the weight for the ${c}^{th}$ cluster-level covariate (taken to be the inverse variance of the cluster means for the ${c}^{th}$ covariate); $C$ is the total number of covariates included in the constrained randomisation; and ${\overline{z} }_{1c}$ and ${\overline{z} }_{0c}$ are the average of the ${c}^{th}$ cluster-level covariates across intervention and control clusters, respectively.

The schemes were then ordered by their balance score and candidate sets formed by taking, in increments of 10%, a percentage of the schemes with the best or worst scores. When the candidate set consisted of 100% of the schemes, this was equivalent to using simple randomisation. A scheme was then selected at random from the candidate set, which determined the treatment allocation for the clusters in that scenario.

### Generation of the outcome observations for each individual within each cluster

The continuous outcome, ${Y}_{ij}$, for the ${i}^{\mathrm{th}}$ ($i=1,\dots ,M$) participant, in the ${j}^{\mathrm{th}}$ ($j=1,\dots ,K$) cluster, was generated from the following linear mixed-effects model:

$${Y}_{ij}={z}_{j}^{T}\gamma +\theta {X}_{j}+{\alpha }_{j}+{\varepsilon }_{ij}$$

where ${z}_{j}$ is the vector of cluster-level covariates, the coefficient vector $\gamma ={2}_{L\times 1}$ represents the prognostic effect of each of the L covariates included in the cluster-level data generation; ${X}_{j}$ is the binary treatment indicator (determined by the selected randomisation scheme); and $\theta$, is the treatment effect. The two variance terms for the random cluster effects (${\alpha }_{j}\sim N(0, {\sigma }_{b}^{2})$) and the residual variance (${\varepsilon }_{ij}\sim N\left(0, {\sigma }_{e}^{2}=1\right)$) together define the intra-cluster correlation $\rho ={\sigma }_{b}^{2}/\left({\sigma }_{b}^{2}+{\sigma }_{e}^{2}\right)$.

### Analysis of outcomes

Given the selected randomisation scheme and individual-level data, the trial was analysed using a linear mixed-effects model, with a random cluster effect and fixed-effects for each of the covariates being adjusted for in the analysis. The model was fitted by restricted maximum likelihood using the lme function from the nlme package in R [28]. An F-test was used to test the treatment effect. Satterthwaite corrected degrees of freedom were used, as they perform better than Kenward-Roger and between-within corrections for parallel CRTs randomising 10 or more clusters [29].

To fully compare the performance of covariate constrained randomisation with simple randomisation and the worst possible allocations, three situations were considered, adjusting for a varying number of covariates relative to those constrained in the randomisation. Firstly, to confirm the need for those covariates balanced in the randomisation to be adjusted in the analysis, when all covariates were balanced in the randomisation, fewer covariates were adjusted in the analysis. Secondly, the same number of covariates were balanced in the randomisation as were adjusted in the analysis. This mimics the situation in which no additional information on prognostic covariates is gained during the trial. The final situation is when information on additional prognostic covariates is obtained during the trial and so those additional covariates are also adjusted for in the analysis. In this case, more covariates were adjusted in the analysis than were balanced in the randomisation.

### Estimation of the power and type I error rate

The process of simulating the CRT, randomisation, outcome generation and analysis was repeated 20,000 times under each scenario, to provide a Monte Carlo standard error of approximately 0.002 for the type I error rate and 0.003 for power [30]. The proportion of the simulations that detected a treatment effect (*p*-value < 0.05) provided the Monte Carlo power ($\theta \ne 0$) and the type I error rate ($\theta =0$).

### Simulation scenarios

Each scenario included five, nine or 13 clusters of size 300 per arm, which are representative of the smaller CRTs which are likely to benefit most from covariate constrained randomisation, since approximately 35% of CRTs randomise less than 20 clusters (Table 2) [2, 31]. We considered a range of intra-cluster correlations: 0.001, 0.01, 0.05 and 0.1, in keeping with those values commonly reported in CRTs [32]. Four independent cluster-level covariates following a Bernoulli distribution with a probability of 0.3 (for comparability with the findings of Li et al. [17]) were included. Each cluster-level covariate had a fixed magnitude of effect (2) on the outcome. Treatment effects were either zero (to assess type I error) or one of three non-zero values to assess the power, selected to ensure power did not reach 100%.

**Table 2:** Summary of the factorial design of the simulation study for the primary objective

Parameter	Values
Number of clusters in each treatment arm (K)	5, 9, 13
Number of observations per cluster (M)	300
Intra-cluster correlation (ρ)	0.001, 0.01, 0.05, 0.1
Standardised treatment effect (θ)	0 or 0.5 (0.2 (13 clusters per arm), 0.25 (9 clusters per arm, ICC = 0.01 or 0.001))
Number of covariates in the data generation model (L)	4
Magnitude of prognostic effect of covariates	2
Number of covariates balanced in the randomisation (C)	1, 2, 3, 4
Candidate set	10%, 20%, …, 90% worst schemes, 100% (simple randomisation), 10%, …, 90% best schemes
Number of covariates adjusted in the analysis (A)	1, 2, 3, 4

### Impact of the prognostic effect and number of covariates adjusted in the analysis under simple randomisation

To investigate the impact of the number of covariates being adjusted in the analysis and the magnitude of their prognostic effect, we conducted further simulations including four, eight or 12 covariates in the data generation model, and with three magnitudes of the prognostic effect of the covariates (0.25, 0.5 and 1.0) (Table 3). These further simulations were conducted for the trial with nine clusters per arm and an intra-cluster correlation coefficient of 0.05, and under simple randomisation only.

**Table 3:** Summary of the factorial design of the simulation study for the secondary objective

Parameter	Values
Number of clusters in each treatment arm (K)	9
Number of observations per cluster (M)	300
Intra-cluster correlation (ρ)	0.05
Standardised treatment effect (θ)	0 or 0.5
Number of covariates in the data generation model (L)	4, 8, 12
Magnitude of prognostic effect of covariates	0.25, 0.5, 1.0
Number of covariates balanced in the randomisation (C)	1, 2, 3, 4
Candidate set	100% (simple randomisation)
Number of covariates adjusted in the analysis (A)	1, 2, 3, 4, 5, 6, 7, 8, 9, 10, 11, 12

## Results

### Type I error under covariate constrained randomisation

**Fig. 1:**
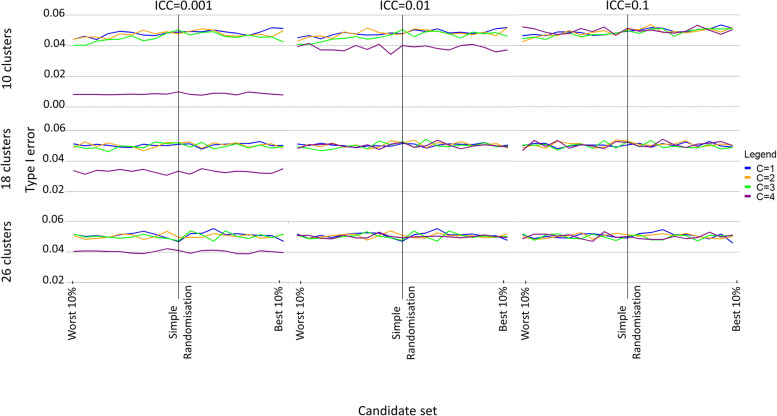
Type I error rate when an increasing number of covariates (C) are balanced in the randomisation and the same number of covariates (A = C) are adjusted in the analysis

When not all of the covariates that were balanced in the randomisation were adjusted in the analysis, the type I error rate was not well maintained under covariate constrained randomisation, becoming increasingly inflated as the candidate set size was restricted (Additional file 1: Fig. 1). When all of the covariates that were balanced in the randomisation were adjusted for in the analysis, as well as when additional covariates were also adjusted for in the analysis, the type I error rate was generally well maintained across the different candidate set sizes (Fig. 1 and Additional file 1: Fig. 2). However, both under simple randomisation and covariate constrained randomisation, if the intra-cluster correlation was particularly small (0.001 and 0.01) the type I error became increasingly conservative as fewer clusters were randomised, even occurring in some scenarios with 26 clusters (Fig. 1 and Additional file 1: Fig. 2).

**Fig. 2:**
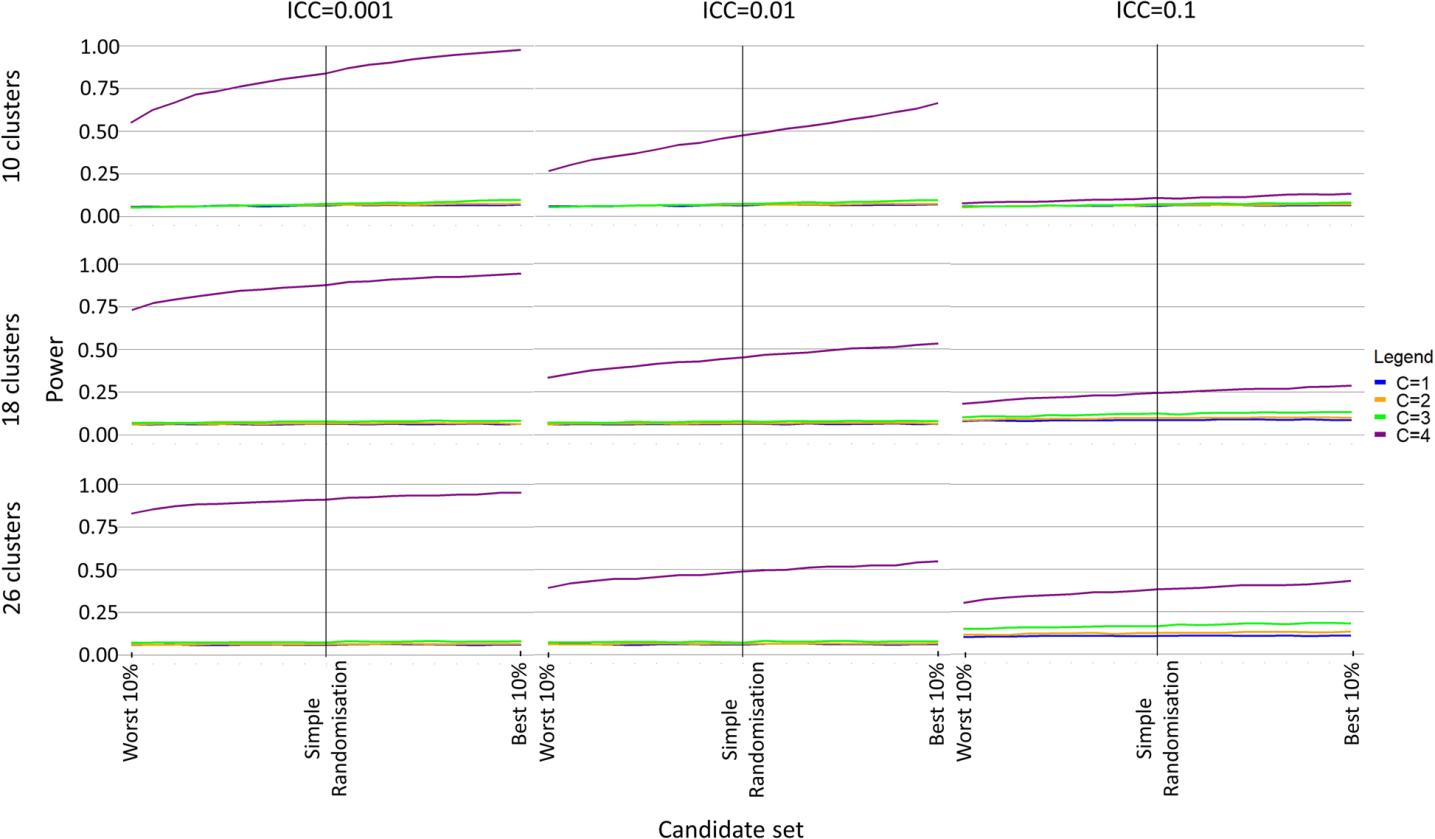
Power when an increasing number of covariates (C) are balanced in the randomisation and the same number of covariates (A = C) are adjusted in the analysis

### Power for covariate constrained randomisation comparing the best to worst balanced randomisation schemes

Here we focus only on those scenarios in which the type I error rate was well maintained, as comparisons of power are otherwise unreasonable. The following scenarios are therefore excluded: covariate constrained randomisation when not all of the covariates balanced in the randomisation were adjusted in the analysis; simple and covariate constrained randomisation when the analysis was adjusted for all of the covariates present and the intra-cluster correlation coefficient was 0.001 (or 0.01 for a total of 10 clusters only).

**Fig. 3:**
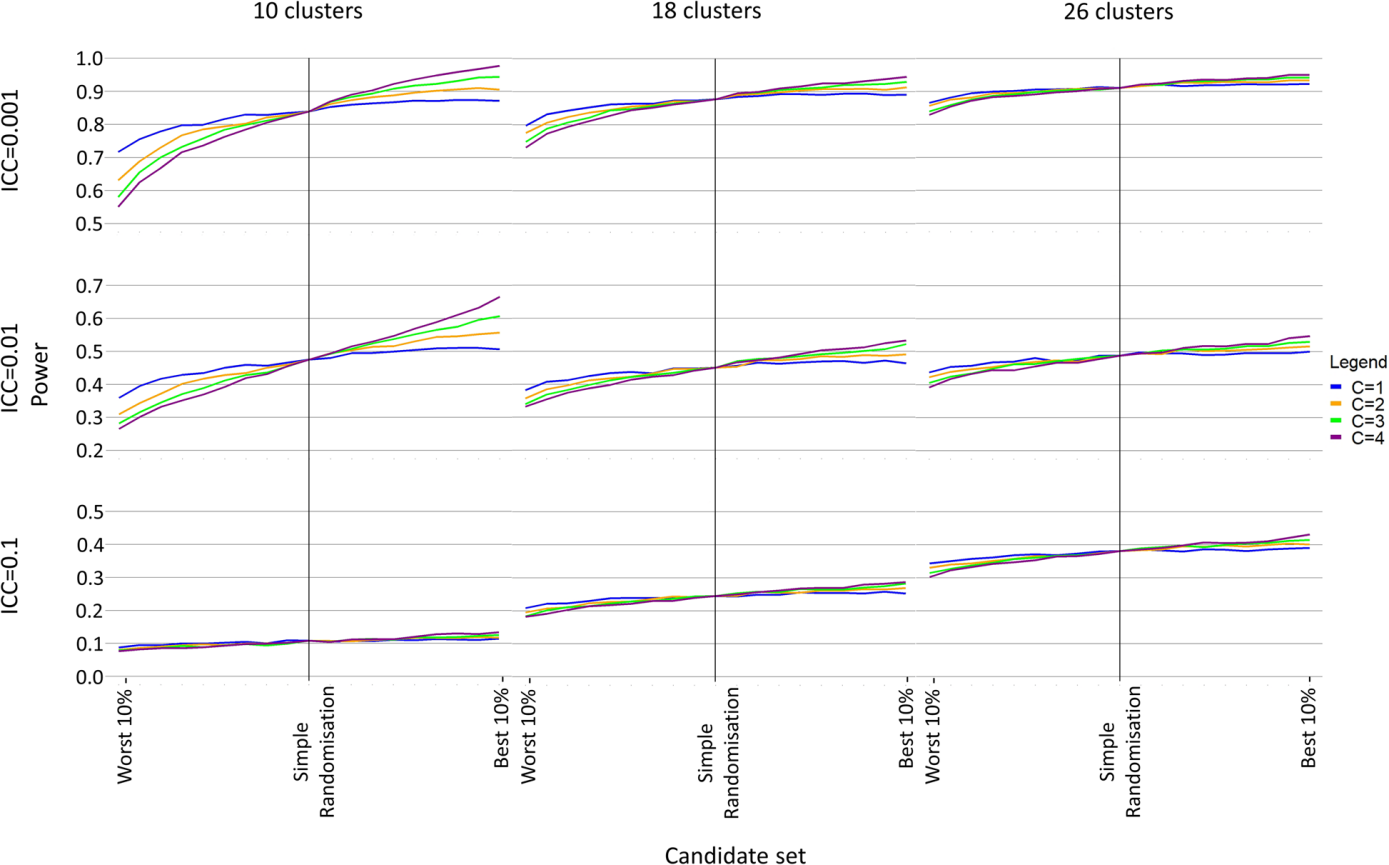
Power when an increasing number of covariates (C ≤ A) are balanced in the randomisation and all four covariates are adjusted in the analysis (A = 4)

Under covariate constrained randomisation, a difference in power of up to approximately 20 percentage points was observed between the best and worst randomisation schemes (Fig. 2, Additional File 2). Power increased as the candidate set was restricted to include only those schemes with the best balance and as more covariates were balanced in the randomisation (Fig. 2, Additional File 2). The difference in power was greatest when the intra-cluster correlation coefficient was small, few clusters were randomised, and more covariates were balanced in the randomisation (Fig. 2, Additional File 2). When all four of the covariates were adjusted in the analysis, the power under simple randomisation could be improved upon by balancing on all or some of those covariates in the randomisation (Fig. 3, Additional File 3). The power gains from balancing on the covariates were most evident when compared to those schemes with the worst balance (Fig. 3, Additional File 3).

### Impact of the prognostic effect and number of covariates adjusted in the analysis under simple randomisation

**Fig. 4:**
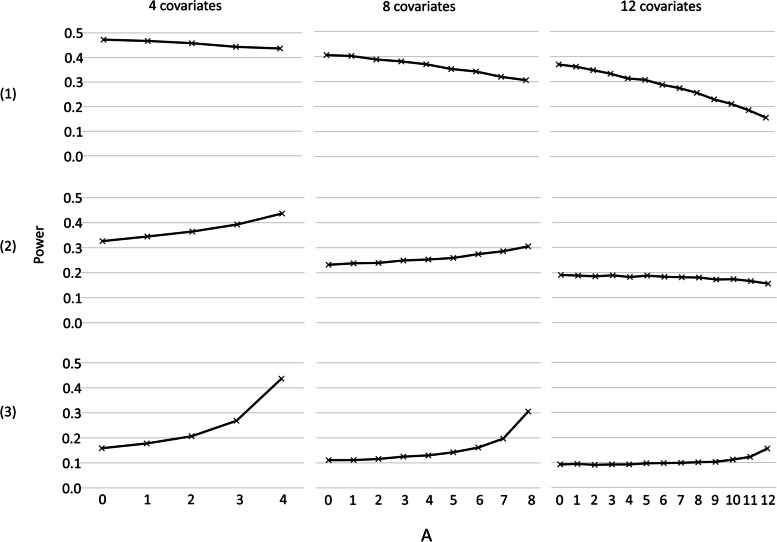
Power under simple randomisation with covariate adjustment, for a data generation model with four, eight or 12 covariates, adjusted for A of the covariates in the analysis, with covariate coefficients of (1) 0.25, (2) 0.5 or (3) 1.0. All with 18 clusters and an intra-cluster correlation coefficient of 0.05. (Total variance changes across scenarios)

Under simple randomisation, the type I error was well maintained under an adjusted analysis regardless of the number of covariates, or the size of their prognostic effect (Additional file 1: Fig. 3). When the prognostic effect was strong and fewer covariates were adjusted in the analysis than were present in the data generation model, a small gain in power was observed with each additional covariate that was adjusted for in the analysis (Fig. 4). If all of the covariates were adjusted for and the prognostic effect was strong, then there was a much larger gain in power (Fig. 4). When the prognostic effect of the covariates was weak, covariate adjustment resulted in a loss of power as more covariates were adjusted for (Fig. 4).

### Application to the motivating example

Based on the findings of the simulation study, it is clear that a covariate constrained randomisation could not be used for the trial in the motivating example if the clinical measure (with intra-cluster correlation 0.001) was chosen, as the type I error rate could not be maintained under an adjusted analysis. This would also be the case if simple randomisation was used with an adjusted analysis and even if some additional clusters could be recruited. Assuming therefore that the process measure was selected as the primary outcome, we performed a covariate constrained randomisation assuming a prognostic value of two for each covariate and a standardised treatment effect of 0.5, the power under a covariate constrained randomisation with a 10% candidate set size was 3 percentage points greater than that averaged over all simple randomisations, whereas the power was 6 percentage points greater than the 10% of schemes with the worst balance, that could be selected under simple randomisation. An additional gain in power could be obtained by gathering information on guideline use and adjusting for it in the analysis. However, despite the benefits in power that can be obtained through covariate constrained randomisation, for the trial in the motivating example the benefit is limited to restricting the possible randomisation schemes to those with the best balance. If more clusters could be recruited then a greater gain in power would be obtained through the use of covariate constrained randomisation.

## Discussion

### Summary of findings

Previous studies have suggested that, in parallel CRTs, covariate constrained randomisation provides only small gains in power over simple randomisation [17, 18]. However, these comparisons have been with the power averaged over all possible randomisation schemes under simple randomisation [17, 18], disregarding the sometimes-considerable variability in power between different allocations of clusters [23]. We have shown that when compared to the worst balanced randomisation schemes, covariate constrained randomisation can provide moderate to substantial improvements in power, even under comparable covariate adjustment in the analysis, and is therefore more beneficial than previously thought. Moreover, when the intra-cluster correlation coefficient is small even an analysis adjusted for all of the covariates constrained in the randomisation and a Satterthwaite small sample correction is unable to maintain the type I error rate. Finally, we found that increasing the number of covariates adjusted in the analysis had a non-linear association with power and in some scenarios, under simple randomisation, a reduction in power was observed when many covariates with a weak prognostic effect were included. This differs from what is known for individually randomised trials with continuous outcomes [25].

### Research in context

Several of our findings echo what was already known [14–18, 25, 33]. Firstly, any covariates that are balanced in the randomisation should be adjusted for in the analysis, to ensure correct type I errors and greatest power. This is supportive of Li’s finding: “analysis-based adjustment is always necessary even after design-based adjustment” [17]. Secondly, assuming all covariates have been adjusted for, covariate constrained randomisation provides only small to moderate gains in power over the average power under simple randomisation [17].

Until now, the implication that the power gains observed under covariate constrained randomisation are due to the analysis adjustment, rather than the act of constraining the randomisation, might have led researchers to conclude that the complexity of implementing covariate constrained randomisation might not be warranted. However, in almost all evaluations of covariate constrained randomisation, performance has been compared to that averaged across all possible simple random allocations, which masks the potential variability in power between different allocations. We therefore compared the performance with the worst set of allocations that could be selected (whilst also adjusting for covariates in the analysis). It is under this comparison that we identified the largest gains in power. For example, with 26 clusters, an intra-cluster correlation coefficient of 0.05, and a candidate set size of 10%, a gain in power of 17.8 percentage points was observed over those allocations with the worst balance, compared to a gain in power of 6.4 percentage points over the average under simple randomisation. Although no trial would employ a method of randomisation that only selects from the worst balanced allocations, this comparison gives an indication of the “worst case scenario” under simple randomisation, which could be avoided by using covariate constrained randomisation. Additionally, we consider the situation where information on some prognostic covariates becomes available during the trial and show the power that can be achieved by adjusting for these additional covariates in the analysis. The magnitude of the power gain over simple randomisation with covariate adjustment is similar to that found by Li et al., but demonstrates the benefit of collecting information on additional prognostic covariates during a trial [17].

Under individual randomisation, with a continuous outcome, it is known that adjustment for covariates improves power, irrespective of the number included and their prognostic value [25]. In contrast, for binary outcomes, caution is required, as over adjustment for covariates without prognostic value can lead to reductions in precision [25]. In our assessment of covariate adjustment for CRTs, we identified that it may result in substantial gains in power for covariates that exert a large influence on the outcome. Yet, despite considering only continuous outcomes, we identified the need for careful consideration of whether to adjust for covariates in the analysis of parallel CRTs: adjusting for too many non-prognostic covariates may result in a loss of power. This is likely to be as a result of the covariate adjustment increasing the standard error of the treatment effect estimate by using additional degrees of freedom, which is not outweighed by the prognostic effect of the covariates reducing the bias in the estimate [25].

Small sample corrections are known to be crucial to obtain nominal type I errors in the analysis of clustered data with fewer than about 40 clusters [29]. However, despite using the Satterwhite small sample correction, when the intra-cluster correlation was small and few clusters were randomised, the type I error became conservative across both simple and covariate constrained randomisation. Whilst perhaps not widely appreciated, we are not the first to observe small sample corrections performing poorly when the intra-cluster correlation coefficient is small: small sample corrected generalised estimating equations and linear mixed-effects models with Kenward-Roger or between-within small sample corrections fail to maintain the type I error rate when the intra-cluster correlation coefficient is small, and therefore are not recommended in these scenarios [29]. However, the Satterthwaite correction has been shown to maintain the type I error rate with small intra-cluster correlations in settings without covariate adjustment and so our finding is likely a problem of the performance of the small sample correction for a covariate adjusted analysis. Future work to investigate the performance of various small sample corrections for a covariate adjusted analysis is required, since it appears to differ to that under an unadjusted analysis. Cluster-level analysis methods have been shown to maintain the type I error rate fairly well when the number of clusters is small [29], however, this has also not been investigated under a covariate adjusted analysis, where the loss of degree of freedom is likely to have a detrimental effect on the performance of these methods.

We observed the power of an analysis that is adjusted for a fixed number of covariates, varies depending on the total number of covariates present in the data generation model. This is a result of a change in the total variance, with a change in the number of covariates in the data generation model; as more covariates are present the total variance increases, resulting in a lower power than when the same number of covariates are adjusted for, but fewer covariates are present. Secondly, the change in power as additional covariates are adjusted for in the analysis is not uniform, there is a greater change when the final covariate present in the data generation model is adjusted in the analysis, than when previous covariates are adjusted for. This is likely to be due to the removal of any remaining bias in the treatment effect estimate, by correctly specifying the analysis model, reducing the variability in the outcome estimate across different allocations, thus reducing the variability in the power.

### Limitations

Throughout this study CRTs with an equal allocation, cross-sectional design, equal size clusters (fixed at 300), a continuous outcome, and binary cluster-level covariates were considered, which may limit the generalisability of the findings. In practice, clusters are unlikely to be of equal size and may be informative of the outcome of interest. This has been shown to result in a reduction in power [34]. Covariate constrained randomisation might be able to mitigate this effect, if the size of the clusters was included as a covariate to be balanced in the randomisation. The balance metric used in the study can also be used for continuous covariates and those with more than two categories, so our methods can naturally be extended to these types of outcomes. Alternative balance metrics exist which have been implemented into software and may perform better for these types of outcomes [19–21]. If known, individual-level covariates could be constrained in the randomisation, and have been in previous studies [17, 18], but practically speaking, information on these is unlikely to be known at the point of randomisation. Under covariate constrained randomisation we also fixed the prognostic effect of the covariates. Under simple randomisation, the size of the prognostic effect of the covariates can influence the power of an adjusted analysis. It is expected that greater power will be achieved under covariate constrained randomisation when those covariates being balanced have a strong prognostic effect.

We have considered CRTs randomising few clusters and used Satterthwaite correction degrees of freedom to account for this, a further correction to the standard errors may have improved the maintenance of the type I error rate. Furthermore, post-randomisation withdrawal of a cluster can occur, which will not only impact the power of the trial, but may also impact the balance of the allocation of the remaining clusters. We do not investigate this here, but the effect on the balance of the allocation of a cluster withdrawing from the trial will depend on the covariate values of the cluster, and will therefore depend entirely on which cluster withdraws from the trial. It is also often the case that not all of the clusters are known a priori, requiring randomisation to be conducted sequentially, in which case covariate constrained randomisation would not be suitable. Our work is also limited to two-arm parallel designs, covariate constrained randomisation is likely to perform differently for other designs of cluster trial. Further work is being conducted for stepped-wedge designs, where thought is needed as to how best to define a balanced allocation and researchers could look at the cluster randomised crossover design, although the impact of an imbalance in cluster-level covariates is likely to be less due to the comparisons made within the same cluster.

We showed here that the greatest power is achieved when the candidate set size is at its smallest. However, care is needed in the application of this finding, as decreasing the size of the candidate set might result in a low absolute number of possible allocations which might be viewed as non-random. For example, for a trial randomising 16 clusters, a 10% candidate set size would include 1,287 randomisation schemes, which is unlikely to be problematic. However, for a trial randomising only eight clusters, a 10% candidate set would include only 7 randomisation schemes, which might be viewed as too few to be considered random. In addition, we did not investigate whether the randomisation schemes forming the candidate sets resulted in cluster allocations being confounded [15, 19]. It is possible that poor balance would be obtained unless two clusters are always allocated to different treatment arms, in this case the allocation of one cluster can be predicted by the allocation of the other which could impact the validity of the randomisation [15, 19]. Increasing the candidate set size may be able to eliminate this problem. Several authors have discussed the potential validity of restricted randomisations, and these may prove useful in determining the appropriate size of the candidate set for a particular covariate constrained randomisation [15, 33, 35, 36].

## Supplementary Information

**Additional file 1: Appendix Figure 1.** Type I error rate when fourcovariates are balanced in the randomisation (C=4) and an increasing number ofcovariates (A≤C) are adjusted in the analysis.

**Additional file 2: Table 1.** Power by candidate set size when an increasing number of covariates (C) are balanced in the randomisation and the same number of covariates are adjusted in the analysis, for trials with 10 clusters and intracluster correlation coefficient (ICC) between 0.001 and 0.1. **Table 2.** Power by candidate set size when an increasing number of covariates (C) are balanced in the randomisation and the same number of covariates are adjusted in the analysis, for trials with 18 clusters and intracluster correlation coefficient (ICC) between 0.001 and 0.1. **Table 3.** Power by candidate set size when an increasing number of covariates (C) are balanced in the randomisation and the same number of covariates are adjusted in the analysis, for trials with 26 clusters and intracluster correlation coefficient (ICC) between 0.001 and 0.1.

**Additional file 3: Table 1.** Power by candidate set size when an increasing number of covariates (C) are balanced in the randomisation and all four covariates are adjusted in the analysis, for trials with 10 clusters and intracluster correlation coefficient (ICC) between 0.001 and 0.1 **Table 2.** Power by candidate set size when an increasing number of covariates (C) are balanced in the randomisation and all four covariates are adjusted in the analysis, for trials with 18 clusters and intracluster correlation coefficient (ICC) between 0.001 and 0.1. **Table 3.** Power by candidate set size when an increasing number of covariates (C) are balanced in the randomisation and all four covariates are adjusted in the analysis, for trials with 26 clusters and intracluster correlation coefficient (ICC) between 0.001 and 0.1.

**Additional file 4.**

## Data Availability

The simulation code that supports the findings of this study is provided in Additional file 4.

## Abbreviation

CRT: Cluster randomised trial
