# Supplementary material for: Mind the gap: covariate constrained randomisation can protect against substantial power loss in parallel cluster randomised trials
Source: BMC Med Res Methodol. 2022 Apr 13;22:111. doi: 10.1186/s12874-022-01588-8 (PMC9006416; doi:10.1186/s12874-022-01588-8)
Supplement: Supplementary file 1 — Additional file 1: Appendix Figure 1. Type I error rate when fourcovariates are balanced in the randomisation (C=4) and an increasing number ofcovariates (A≤C) are adjusted in the analysis. [file 12874_2022_1588_MOESM1_ESM.docx]

**Appendix**


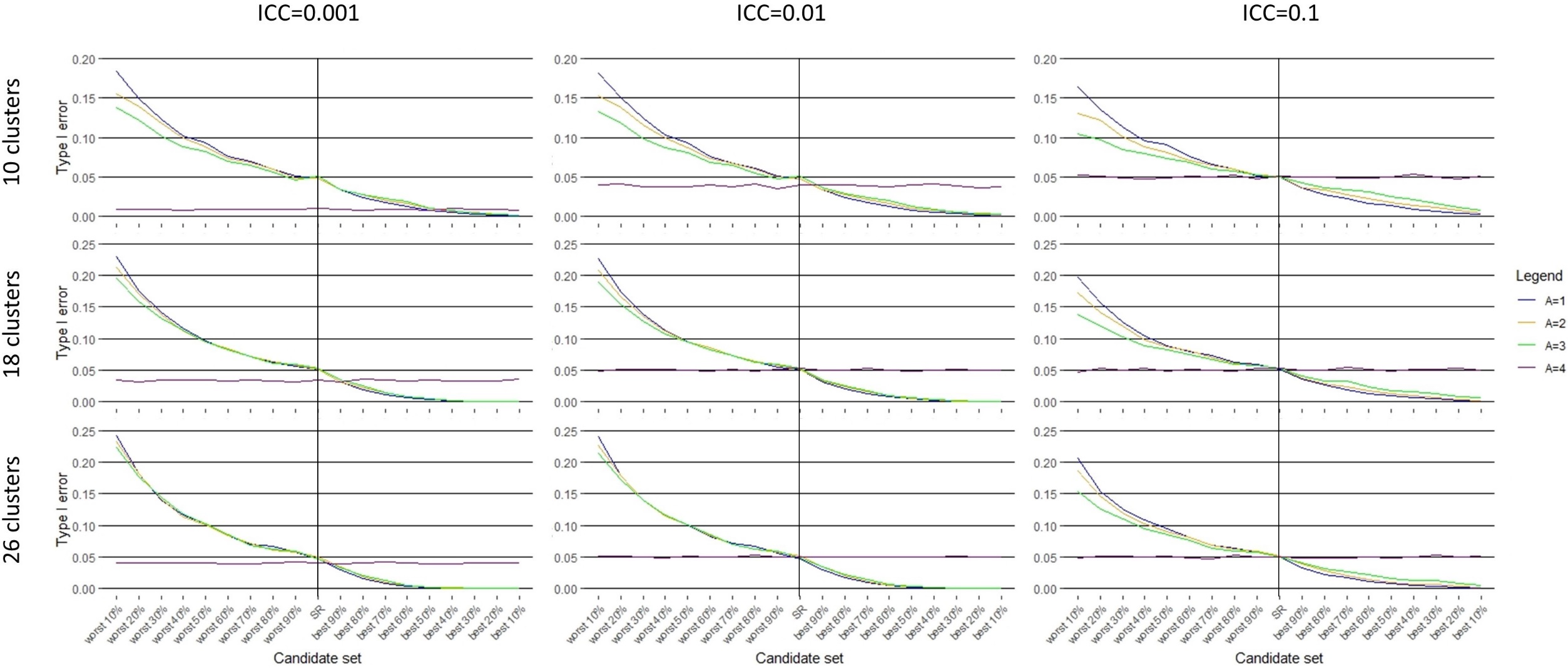
Appendix Figure 1: Type I error rate when four covariates are balanced in the randomisation (C=4) and an increasing number of covariates (A≤C) are adjusted in the analysis.


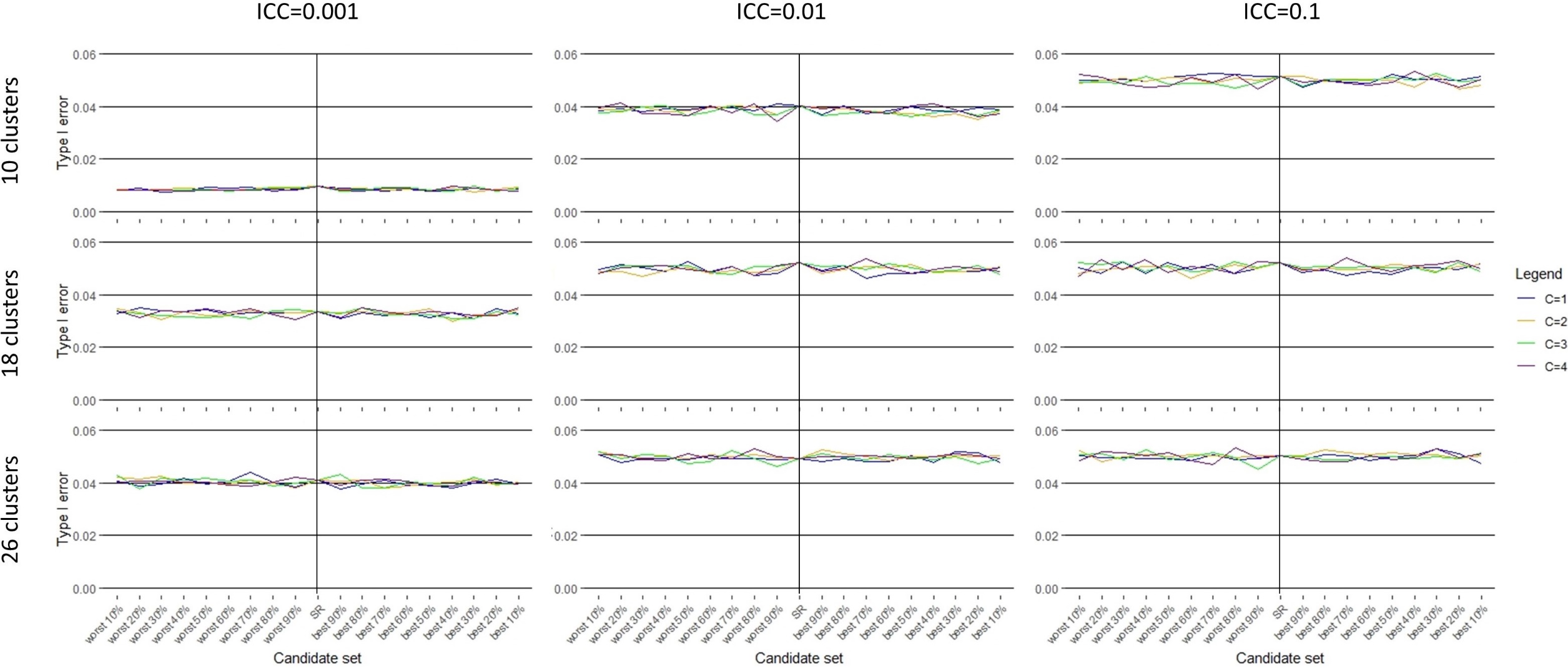
Appendix Figure 2: Type I error rate when an increasing number of covariates (C≤A) are balanced in the randomisation and all four covariates are adjusted in the analysis (A=4).


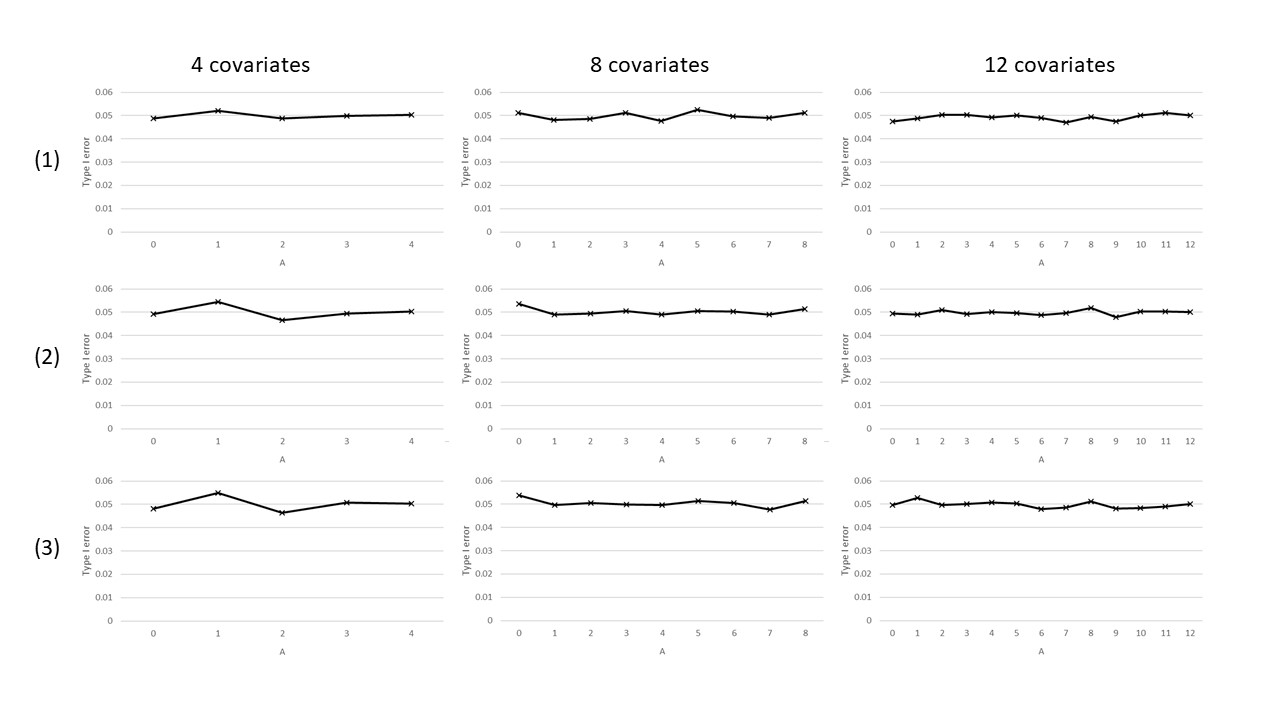
Appendix Figure 3: Type I error rate under simple randomisation for 18 clusters, an intra-cluster correlation coefficient of 0.05, for a data generation model with four, eight or 12 covariates, adjusted for an increasing number (A) of the covariates in the analysis, with covariate coefficients of (1) 0.25, (2) 0.5 or (3) 1.0.
