# Supplementary material for: Mind the gap: covariate constrained randomisation can protect against substantial power loss in parallel cluster randomised trials
Source: BMC Med Res Methodol. 2022 Apr 13;22:111. doi: 10.1186/s12874-022-01588-8 (PMC9006416; doi:10.1186/s12874-022-01588-8)
Supplement: Supplementary file 3 — Additional file 3: Table 1. Power by candidate set size when an increasing number of covariates (C) are balanced in the randomisation and all four covariates are adjusted in the analysis, for trials with 10 clusters and intracluster correlation coefficient (ICC) between 0.001 and 0.1 Table 2. Power by candidate set size when an increasing number of covariates (C) are balanced in the randomisation and all four covariates are adjusted in the analysis, for trials with 18 clusters and intracluster correlation coefficient (ICC) between 0.001 and 0.1. Table 3. Power by candidate set size when an increasing number of covariates (C) are balanced in the randomisation and all four covariates are adjusted in the analysis, for trials with 26 clusters and intracluster correlation coefficient (ICC) between 0.001 and 0.1. [file 12874_2022_1588_MOESM3_ESM.pdf]

Table 1: Power by candidate set size when an increasing number of covariates (C) are balanced in the randomisation and all four covariates are adjusted in the analysis, for trials with 10 clusters and intracluster correlation coefficient (ICC) between 0.001 and 0.1.

		Power by candidate set size																		
ICC	C	10% Worst	20% Worst	30% Worst	40% Worst	50% Worst	60% Worst	70% Worst	80% Worst	90% Worst	SR	90% Best	80% Best	70% Best	60% Best	50% Best	40% Best	30% Best	20% Best	10% Best
0.001	1	71.63	75.53	77.87	79.69	79.88	81.59	82.96	82.86	83.46	83.89	85.27	85.92	86.36	86.70	87.20	87.04	87.36	87.31	87.10
	2	63.07	68.78	72.95	76.69	78.50	79.34	80.17	82.09	82.77	83.89	86.11	87.30	88.18	88.75	89.61	90.16	90.57	90.95	90.48
	3	58.06	65.51	69.99	73.12	75.72	78.35	79.79	81.13	82.37	83.89	86.77	88.31	89.32	90.77	91.71	92.24	93.12	94.16	94.39
	4	55.05	62.42	66.72	71.56	73.59	76.20	78.42	80.51	82.21	83.89	86.88	88.95	90.26	92.23	93.62	94.75	95.81	96.71	97.66
0.01	1	35.92	39.53	41.73	42.88	43.44	45.02	45.91	45.67	46.58	47.52	47.98	49.43	49.53	49.91	50.42	50.86	51.04	51.00	50.61
	2	30.93	34.40	37.23	40.20	41.67	42.84	43.40	45.05	45.98	47.52	49.43	50.36	51.37	51.59	53.03	54.34	54.57	55.19	55.66
	3	28.13	31.62	34.55	37.09	38.89	41.12	42.82	43.57	45.47	47.52	49.20	50.75	52.41	53.72	55.15	56.51	57.48	59.54	60.63
	4	26.50	30.20	33.23	35.17	36.97	39.22	41.90	43.07	45.59	47.52	49.35	51.49	52.95	54.67	56.89	58.81	61.03	63.20	66.47
0.05	1	12.60	13.76	14.31	15.22	14.78	15.33	16.17	15.55	16.40	16.21	16.56	16.76	16.73	17.14	17.14	17.39	17.35	17.17	17.66
	2	11.46	12.46	13.37	13.77	14.53	14.55	15.00	15.42	15.61	16.21	16.68	17.12	17.38	17.83	18.08	18.62	18.21	18.91	18.71
	3	10.78	11.58	12.34	13.38	13.26	14.42	14.87	14.54	15.48	16.21	16.37	17.37	17.53	17.65	18.76	18.92	19.15	19.77	20.49
	4	10.32	11.33	12.20	12.55	13.09	13.63	14.34	15.03	15.42	16.21	16.39	17.53	17.34	18.39	19.35	20.04	20.80	20.93	22.19
0.1	1	8.73	9.49	9.39	9.97	9.87	10.17	10.46	9.94	10.91	10.76	10.50	10.63	10.66	11.07	10.96	11.24	11.11	10.98	11.43
	2	8.05	8.61	8.98	9.38	9.71	9.46	9.87	9.94	10.13	10.76	10.71	10.61	10.99	11.21	11.65	11.64	11.58	11.90	11.69
	3	7.87	8.15	8.55	9.20	8.76	9.34	9.73	9.36	9.92	10.76	10.42	11.13	11.33	11.04	11.92	11.76	11.87	12.21	12.54
	4	7.58	8.20	8.49	8.50	8.78	9.28	9.73	9.87	10.15	10.76	10.38	11.11	11.24	11.18	11.99	12.74	13.00	12.77	13.36

Table 2: Power by candidate set size when an increasing number of covariates (C) are balanced in the randomisation and all four covariates are adjusted in the analysis, for trials with 18 clusters and intracluster correlation coefficient (ICC) between 0.001 and 0.1.

		Power by candidate set size																		
ICC	C	10% Worst	20% Worst	30% Worst	40% Worst	50% Worst	60% Worst	70% Worst	80% Worst	90% Worst	SR	90% Best	80% Best	70% Best	60% Best	50% Best	40% Best	30% Best	20% Best	10% Best
0.001	1	79.70	83.16	84.24	85.25	86.14	86.36	86.37	87.35	87.34	87.71	88.45	88.71	89.24	89.26	89.04	89.37	89.47	89.00	89.11
	2	77.47	80.63	82.34	83.62	84.49	85.53	85.93	87.09	87.06	87.71	88.94	89.29	89.81	90.37	90.75	90.79	90.91	90.56	91.30
	3	74.83	78.84	80.66	82.14	84.35	84.75	85.57	86.41	87.07	87.71	89.50	89.71	90.52	90.87	91.25	92.00	92.18	92.37	93.01
	4	73.12	77.30	79.36	81.10	82.77	84.41	85.18	86.17	86.87	87.71	89.50	89.91	91.00	91.59	92.49	92.51	93.16	93.83	94.49
0.01	1	38.19	40.74	41.24	42.43	43.35	43.68	43.32	44.79	44.75	45.02	45.58	46.54	46.21	46.60	46.88	47.00	46.48	47.18	46.35
	2	35.69	38.45	39.65	41.17	41.81	42.24	43.20	44.62	44.59	45.02	45.29	47.20	47.26	47.61	48.50	48.24	48.85	48.60	49.05
	3	33.95	36.84	38.26	39.80	41.14	42.22	42.78	43.45	44.43	45.02	46.96	47.65	47.90	48.46	49.08	49.49	50.09	50.59	52.14
	4	33.18	35.42	37.42	38.72	39.77	41.35	42.26	42.72	44.14	45.02	46.60	47.32	48.03	49.16	50.28	50.72	51.19	52.41	53.21
0.05	1	36.95	39.06	39.88	41.19	41.78	42.48	42.12	43.72	43.28	43.40	44.13	45.08	45.10	45.29	45.22	45.34	45.35	45.77	44.98
	2	34.62	37.15	38.09	39.25	40.49	40.69	41.80	42.91	42.76	43.40	44.14	45.77	46.05	45.94	46.81	46.64	47.42	46.97	47.89
	3	33.08	35.40	36.98	38.29	39.91	40.83	41.42	41.89	43.10	43.40	45.27	46.43	46.28	47.02	47.47	47.82	48.73	49.09	50.47
	4	31.96	34.06	36.13	37.33	38.61	40.05	40.86	41.46	42.88	43.40	44.72	45.80	46.83	48.00	48.59	49.16	49.81	50.95	51.39
0.1	1	20.81	22.10	22.24	22.94	23.74	23.92	23.84	23.98	24.17	24.45	24.33	24.89	24.82	25.55	25.39	25.41	25.25	25.79	25.20
	2	19.47	20.65	21.00	22.26	22.67	22.78	23.57	24.35	24.09	24.45	24.44	25.77	25.44	25.50	26.12	26.04	26.56	26.47	26.92
	3	18.34	20.11	21.10	21.35	22.16	22.78	22.98	23.64	24.34	24.45	25.30	25.77	25.61	26.60	26.42	26.37	27.04	27.46	28.27
	4	18.16	19.10	20.28	21.33	21.74	22.12	22.95	23.01	23.88	24.45	24.94	25.62	26.14	26.75	26.98	26.87	27.91	28.22	28.70

Table 3: Power by candidate set size when an increasing number of covariates (C) are balanced in the randomisation and all four covariates are adjusted in the analysis, for trials with 26 clusters and intracluster correlation coefficient (ICC) between 0.001 and 0.1.

		Power by candidate set size																		
ICC	C	10% Worst	20% Worst	30% Worst	40% Worst	50% Worst	60% Worst	70% Worst	80% Worst	90% Worst	SR	90% Best	80% Best	70% Best	60% Best	50% Best	40% Best	30% Best	20% Best	10% Best
0.001	1	86.56	88.12	89.43	89.97	90.16	90.53	90.65	90.76	91.31	91.02	91.87	92.06	91.62	91.87	91.97	92.25	92.10	92.12	92.27
	2	85.63	87.52	88.16	89.35	89.53	89.88	90.39	90.76	90.91	91.02	91.62	92.10	92.72	92.56	92.91	92.81	92.77	93.33	93.42
	3	83.91	85.90	87.50	88.60	89.14	89.79	90.14	90.53	90.49	91.02	92.10	91.91	93.06	93.12	93.29	93.57	93.63	94.21	94.17
	4	82.87	85.35	87.14	88.25	88.64	89.11	89.71	90.10	90.80	91.02	92.11	92.41	93.16	93.60	93.48	93.96	94.10	95.01	95.06
0.01	1	43.70	45.36	45.72	46.76	46.95	48.06	47.05	47.44	48.78	48.74	49.76	49.44	49.43	48.99	49.06	49.56	49.53	49.52	49.97
	2	42.26	43.83	44.67	45.33	46.27	46.91	47.37	46.98	47.93	48.74	49.41	49.25	50.73	50.14	50.30	50.54	50.82	51.21	51.56
	3	40.54	42.39	43.39	44.81	46.09	46.25	47.10	47.75	48.41	48.74	49.43	50.35	50.62	50.59	50.87	51.62	51.64	52.63	52.99
	4	39.13	41.72	43.20	44.38	44.36	45.51	46.61	46.58	47.70	48.74	49.44	49.73	51.07	51.69	51.57	52.37	52.25	54.10	54.69
0.05	1	59.37	60.94	62.58	63.03	63.23	64.31	63.53	64.31	65.30	65.37	66.14	66.27	66.21	66.15	65.85	66.47	66.36	66.66	67.19
	2	57.18	59.80	60.40	61.22	62.52	62.91	63.64	64.49	64.67	65.37	65.90	66.23	67.48	66.65	67.50	67.58	67.84	67.98	68.72
	3	55.39	57.75	59.55	60.89	62.50	62.40	62.98	64.57	64.51	65.37	66.36	67.14	67.96	68.09	68.07	68.60	69.06	69.75	70.21
	4	53.98	57.14	58.91	60.28	60.71	62.05	63.04	63.16	64.41	65.37	66.50	66.90	67.93	68.84	68.64	69.86	69.70	70.83	71.74
0.1	1	34.36	35.05	35.71	36.16	36.81	37.14	36.85	37.31	37.93	38.10	38.54	38.27	37.95	38.61	38.50	38.07	38.53	38.82	39.06
	2	33.00	34.02	34.34	35.09	35.75	36.55	36.43	36.79	37.40	38.10	38.37	38.58	39.54	39.42	39.78	39.44	39.96	40.36	40.11
	3	31.48	32.72	33.70	34.54	35.65	36.06	36.66	36.84	37.17	38.10	38.89	39.29	39.78	39.23	40.00	40.38	40.57	41.19	41.48
	4	30.19	32.28	33.27	34.18	34.70	35.28	36.40	36.55	37.19	38.10	38.57	39.03	39.83	40.68	40.49	40.64	41.06	42.07	43.08
