# Supplementary material for: Mind the gap: covariate constrained randomisation can protect against substantial power loss in parallel cluster randomised trials
Source: BMC Med Res Methodol. 2022 Apr 13;22:111. doi: 10.1186/s12874-022-01588-8 (PMC9006416; doi:10.1186/s12874-022-01588-8)
Supplement: Supplementary file 4 — Additional file 4. [file 12874_2022_1588_MOESM4_ESM.docx]

## R script to simulate data for a cross-sectional, two-arm parallel cluster randomised trial with a continuous outcome and four binary cluster-level covariates. Simple or covariate constrained randomisation are used to allocate the clusters 1:1 to treatment or control groups. The data is then analysed, adjusting for a varying number of covaariates and the Monte Carlo type I error rate and power estimated.

##Setup – instal required library and set seed##

library("nlme")

set.seed(120113)

### Define the values of the parameters that vary between scenarios ###

rho<-0.001 #ICC

nClust<-10 #number of clusters

######################################################

### Define the rest of the parameters required for the setup ###

S<-1000 #number of simulated randomisation schemes

nsim<-20000 #total number of simulations

errorSR_UN<-0 #set a counter for error messages from analysis

errorSR_AD<-0 #as above

errorB1_UN<-0 #as above

errorB1_AD<-0 #as above

errorB2_UN<-0 #as above

errorB2_AD<-0 #as above

ClustSize<-300 #cluster size

nCC<-4 #number of binary cluster-level covariates

obs=nClust*ClustSize #number of observations across the trial

lambda<-sqrt(rho/(1-rho)) #sig_b/sig_e

sig_e<-2 #sig_e (error SD)

theta<-0.5 #treatment effect

gamma<- rep(2,nCC) #binary cluster-level covariate coefficients

############

## Initiation ##

############

### Create the cluster and individual identifiers ###

cl<-1:nClust #list of cluster ID numbers

cluster=rep(1:nClust,each=ClustSize) #list the cluster IDs for all individuals in the study

id=rep(seq(1,ClustSize),nClust) #list the individual IDs for all individuals in the study

###

# create matrices to store the results of the simulations in

## Simple randomisation ##

# Unadjusted analysis:

SRunALPHAtab<-matrix(NA,nsim)

SRunESTMtab<-matrix(NA,nsim)

SRunPOWERtab<-matrix(NA,nsim)

SRunBIAStab<-matrix(NA,nsim)

# Adjusted analysis:

SRadALPHAtab<-matrix(NA,nsim,nCC)

SRadESTMtab<-matrix(NA,nsim,nCC)

SRadPOWERtab<-matrix(NA,nsim,nCC)

SRadBIAStab<-matrix(NA,nsim,nCC)

colnames(SRadALPHAtab)<-colnames(SRadESTMtab)<-colnames(SRadPOWERtab)<-colnames(SRadBIAStab)<-

c("A = 1","A = 2","A = 3","A = 4") #columns for the number of covariates adjusted in the analysis

## B2/B balance metric ##

# Unadjusted analysis: #

#Best 10% of schemes

B2unALPHAtab_b10<-matrix(NA,nsim,nCC)

B2unESTMtab_b10<-matrix(NA,nsim,nCC)

B2unPOWERtab_b10<-matrix(NA,nsim,nCC)

B2unBIAStab_b10<-matrix(NA,nsim,nCC)

colnames(B2unALPHAtab_b10)<-colnames(B2unESTMtab_b10)<-colnames(B2unPOWERtab_b10)<-colnames(B2unBIAStab_b10)<-

c("C = 1","C = 2","C = 3","C = 4") #columns for the number of covariates constrained in the randomisation

#best 20% of schemes

B2unALPHAtab_b20<-matrix(NA,nsim,nCC)

B2unESTMtab_b20<-matrix(NA,nsim,nCC)

B2unPOWERtab_b20<-matrix(NA,nsim,nCC)

B2unBIAStab_b20<-matrix(NA,nsim,nCC)

colnames(B2unALPHAtab_b20)<-colnames(B2unESTMtab_b20)<-colnames(B2unPOWERtab_b20)<-colnames(B2unBIAStab_b20)<-

c("C = 1","C = 2","C = 3","C = 4")

#best 30% of schemes

B2unALPHAtab_b30<-matrix(NA,nsim,nCC)

B2unESTMtab_b30<-matrix(NA,nsim,nCC)

B2unPOWERtab_b30<-matrix(NA,nsim,nCC)

B2unBIAStab_b30<-matrix(NA,nsim,nCC)

colnames(B2unALPHAtab_b30)<-colnames(B2unESTMtab_b30)<-colnames(B2unPOWERtab_b30)<-colnames(B2unBIAStab_b30)<-

c("C = 1","C = 2","C = 3","C = 4")

#best 40% of schemes

B2unALPHAtab_b40<-matrix(NA,nsim,nCC)

B2unESTMtab_b40<-matrix(NA,nsim,nCC)

B2unPOWERtab_b40<-matrix(NA,nsim,nCC)

B2unBIAStab_b40<-matrix(NA,nsim,nCC)

colnames(B2unALPHAtab_b40)<-colnames(B2unESTMtab_b40)<-colnames(B2unPOWERtab_b40)<-colnames(B2unBIAStab_b40)<-

c("C = 1","C = 2","C = 3","C = 4")

#best 50% of schemes

B2unALPHAtab_b50<-matrix(NA,nsim,nCC)

B2unESTMtab_b50<-matrix(NA,nsim,nCC)

B2unPOWERtab_b50<-matrix(NA,nsim,nCC)

B2unBIAStab_b50<-matrix(NA,nsim,nCC)

colnames(B2unALPHAtab_b50)<-colnames(B2unESTMtab_b50)<-colnames(B2unPOWERtab_b50)<-colnames(B2unBIAStab_b50)<-

c("C = 1","C = 2","C = 3","C = 4")

#best 60% of schemes

B2unALPHAtab_b60<-matrix(NA,nsim,nCC)

B2unESTMtab_b60<-matrix(NA,nsim,nCC)

B2unPOWERtab_b60<-matrix(NA,nsim,nCC)

B2unBIAStab_b60<-matrix(NA,nsim,nCC)

colnames(B2unALPHAtab_b60)<-colnames(B2unESTMtab_b60)<-colnames(B2unPOWERtab_b60)<-colnames(B2unBIAStab_b60)<-

c("C = 1","C = 2","C = 3","C = 4")

#best 70% of schemes

B2unALPHAtab_b70<-matrix(NA,nsim,nCC)

B2unESTMtab_b70<-matrix(NA,nsim,nCC)

B2unPOWERtab_b70<-matrix(NA,nsim,nCC)

B2unBIAStab_b70<-matrix(NA,nsim,nCC)

colnames(B2unALPHAtab_b70)<-colnames(B2unESTMtab_b70)<-colnames(B2unPOWERtab_b70)<-colnames(B2unBIAStab_b70)<-

c("C = 1","C = 2","C = 3","C = 4")

#best 80% of schemes

B2unALPHAtab_b80<-matrix(NA,nsim,nCC)

B2unESTMtab_b80<-matrix(NA,nsim,nCC)

B2unPOWERtab_b80<-matrix(NA,nsim,nCC)

B2unBIAStab_b80<-matrix(NA,nsim,nCC)

colnames(B2unALPHAtab_b80)<-colnames(B2unESTMtab_b80)<-colnames(B2unPOWERtab_b80)<-colnames(B2unBIAStab_b80)<-

c("C = 1","C = 2","C = 3","C = 4")

#best 90% of schemes

B2unALPHAtab_b90<-matrix(NA,nsim,nCC)

B2unESTMtab_b90<-matrix(NA,nsim,nCC)

B2unPOWERtab_b90<-matrix(NA,nsim,nCC)

B2unBIAStab_b90<-matrix(NA,nsim,nCC)

colnames(B2unALPHAtab_b90)<-colnames(B2unESTMtab_b90)<-colnames(B2unPOWERtab_b90)<-colnames(B2unBIAStab_b90)<-

c("C = 1","C = 2","C = 3","C = 4")

#worst 10% of schemes

B2unALPHAtab_w10<-matrix(NA,nsim,nCC)

B2unESTMtab_w10<-matrix(NA,nsim,nCC)

B2unPOWERtab_w10<-matrix(NA,nsim,nCC)

B2unBIAStab_w10<-matrix(NA,nsim,nCC)

colnames(B2unALPHAtab_w10)<-colnames(B2unESTMtab_w10)<-colnames(B2unPOWERtab_w10)<-colnames(B2unBIAStab_w10)<-

c("C = 1","C = 2","C = 3","C = 4")

#worst 20% of schemes

B2unALPHAtab_w20<-matrix(NA,nsim,nCC)

B2unESTMtab_w20<-matrix(NA,nsim,nCC)

B2unPOWERtab_w20<-matrix(NA,nsim,nCC)

B2unBIAStab_w20<-matrix(NA,nsim,nCC)

colnames(B2unALPHAtab_w20)<-colnames(B2unESTMtab_w20)<-colnames(B2unPOWERtab_w20)<-colnames(B2unBIAStab_w20)<-

c("C = 1","C = 2","C = 3","C = 4")

#worst 30% of schemes

B2unALPHAtab_w30<-matrix(NA,nsim,nCC)

B2unESTMtab_w30<-matrix(NA,nsim,nCC)

B2unPOWERtab_w30<-matrix(NA,nsim,nCC)

B2unBIAStab_w30<-matrix(NA,nsim,nCC)

colnames(B2unALPHAtab_w30)<-colnames(B2unESTMtab_w30)<-colnames(B2unPOWERtab_w30)<-colnames(B2unBIAStab_w30)<-

c("C = 1","C = 2","C = 3","C = 4")

#worst 40% of schemes

B2unALPHAtab_w40<-matrix(NA,nsim,nCC)

B2unESTMtab_w40<-matrix(NA,nsim,nCC)

B2unPOWERtab_w40<-matrix(NA,nsim,nCC)

B2unBIAStab_w40<-matrix(NA,nsim,nCC)

colnames(B2unALPHAtab_w40)<-colnames(B2unESTMtab_w40)<-colnames(B2unPOWERtab_w40)<-colnames(B2unBIAStab_w40)<-

c("C = 1","C = 2","C = 3","C = 4")

#worst 50% of schemes

B2unALPHAtab_w50<-matrix(NA,nsim,nCC)

B2unESTMtab_w50<-matrix(NA,nsim,nCC)

B2unPOWERtab_w50<-matrix(NA,nsim,nCC)

B2unBIAStab_w50<-matrix(NA,nsim,nCC)

colnames(B2unALPHAtab_w50)<-colnames(B2unESTMtab_w50)<-colnames(B2unPOWERtab_w50)<-colnames(B2unBIAStab_w50)<-

c("C = 1","C = 2","C = 3","C = 4")

#worst 60% of schemes

B2unALPHAtab_w60<-matrix(NA,nsim,nCC)

B2unESTMtab_w60<-matrix(NA,nsim,nCC)

B2unPOWERtab_w60<-matrix(NA,nsim,nCC)

B2unBIAStab_w60<-matrix(NA,nsim,nCC)

colnames(B2unALPHAtab_w60)<-colnames(B2unESTMtab_w60)<-colnames(B2unPOWERtab_w60)<-colnames(B2unBIAStab_w60)<-

c("C = 1","C = 2","C = 3","C = 4")

#worst 70% of schemes

B2unALPHAtab_w70<-matrix(NA,nsim,nCC)

B2unESTMtab_w70<-matrix(NA,nsim,nCC)

B2unPOWERtab_w70<-matrix(NA,nsim,nCC)

B2unBIAStab_w70<-matrix(NA,nsim,nCC)

colnames(B2unALPHAtab_w70)<-colnames(B2unESTMtab_w70)<-colnames(B2unPOWERtab_w70)<-colnames(B2unBIAStab_w70)<-

c("C = 1","C = 2","C = 3","C = 4")

#worst 80% of schemes

B2unALPHAtab_w80<-matrix(NA,nsim,nCC)

B2unESTMtab_w80<-matrix(NA,nsim,nCC)

B2unPOWERtab_w80<-matrix(NA,nsim,nCC)

B2unBIAStab_w80<-matrix(NA,nsim,nCC)

colnames(B2unALPHAtab_w80)<-colnames(B2unESTMtab_w80)<-colnames(B2unPOWERtab_w80)<-colnames(B2unBIAStab_w80)<-

c("C = 1","C = 2","C = 3","C = 4")

#worst 90% of schemes

B2unALPHAtab_w90<-matrix(NA,nsim,nCC)

B2unESTMtab_w90<-matrix(NA,nsim,nCC)

B2unPOWERtab_w90<-matrix(NA,nsim,nCC)

B2unBIAStab_w90<-matrix(NA,nsim,nCC)

colnames(B2unALPHAtab_w90)<-colnames(B2unESTMtab_w90)<-colnames(B2unPOWERtab_w90)<-colnames(B2unBIAStab_w90)<-

c("C = 1","C = 2","C = 3","C = 4")

## B2/B balance metric ##

# Adjusted analysis: #

#constrained on 1 covariate, adjusted for 1 covariate

B2B1s1adALPHAtab<-matrix(NA,nsim,18)

B2B1s1adESTMtab<-matrix(NA,nsim,18)

B2B1s1adPOWERtab<-matrix(NA,nsim,18)

B2B1s1adBIAStab<-matrix(NA,nsim,18)

colnames(B2B1s1adALPHAtab)<-colnames(B2B1s1adESTMtab)<-colnames(B2B1s1adPOWERtab)<-colnames(B2B1s1adBIAStab)<-

("b10","b20","b30","b40","b50","b60","b70","b80","b90","w90","w80","w70","w60","w50","w40","w30","w20","w10")

#constrained on 2 covariate, adjusted for 1 covariate

B2B1s2adALPHAtab<-matrix(NA,nsim,18)

B2B1s2adESTMtab<-matrix(NA,nsim,18)

B2B1s2adPOWERtab<-matrix(NA,nsim,18)

B2B1s2adBIAStab<-matrix(NA,nsim,18)

colnames(B2B1s2adALPHAtab)<-colnames(B2B1s2adESTMtab)<-colnames(B2B1s2adPOWERtab)<-colnames(B2B1s2adBIAStab)<-

c("b10","b20","b30","b40","b50","b60","b70","b80","b90","w90","w80","w70","w60","w50","w40","w30","w20","w10")

#constrained on 1 covariate, adjusted for 3 covariate

B2B1s3adALPHAtab<-matrix(NA,nsim,18)

B2B1s3adESTMtab<-matrix(NA,nsim,18)

B2B1s3adPOWERtab<-matrix(NA,nsim,18)

B2B1s3adBIAStab<-matrix(NA,nsim,18)

colnames(B2B1s3adALPHAtab)<-colnames(B2B1s3adESTMtab)<-colnames(B2B1s3adPOWERtab)<-colnames(B2B1s3adBIAStab)<-

c("b10","b20","b30","b40","b50","b60","b70","b80","b90","w90","w80","w70","w60","w50","w40","w30","w20","w10")

#constrained on 1 covariate, adjusted for 4 covariate

B2B1s4adALPHAtab<-matrix(NA,nsim,18)

B2B1s4adESTMtab<-matrix(NA,nsim,18)

B2B1s4adPOWERtab<-matrix(NA,nsim,18)

B2B1s4adBIAStab<-matrix(NA,nsim,18)

colnames(B2B1s4adALPHAtab)<-colnames(B2B1s4adESTMtab)<-colnames(B2B1s4adPOWERtab)<-colnames(B2B1s4adBIAStab)<-

c("b10","b20","b30","b40","b50","b60","b70","b80","b90","w90","w80","w70","w60","w50","w40","w30","w20","w10")

#constrained on 2 covariate, adjusted for 1 covariate

B2B2s1adALPHAtab<-matrix(NA,nsim,18)

B2B2s1adESTMtab<-matrix(NA,nsim,18)

B2B2s1adPOWERtab<-matrix(NA,nsim,18)

B2B2s1adBIAStab<-matrix(NA,nsim,18)

colnames(B2B2s1adALPHAtab)<-colnames(B2B2s1adESTMtab)<-colnames(B2B2s1adPOWERtab)<-colnames(B2B2s1adBIAStab)<-

c("b10","b20","b30","b40","b50","b60","b70","b80","b90","w90","w80","w70","w60","w50","w40","w30","w20","w10")

#constrained on 2 covariate, adjusted for 2 covariate

B2B2s2adALPHAtab<-matrix(NA,nsim,18)

B2B2s2adESTMtab<-matrix(NA,nsim,18)

B2B2s2adPOWERtab<-matrix(NA,nsim,18)

B2B2s2adBIAStab<-matrix(NA,nsim,18)

colnames(B2B2s2adALPHAtab)<-colnames(B2B2s2adESTMtab)<-colnames(B2B2s2adPOWERtab)<-colnames(B2B2s2adBIAStab)<-

c("b10","b20","b30","b40","b50","b60","b70","b80","b90","w90","w80","w70","w60","w50","w40","w30","w20","w10")

#constrained on 2 covariate, adjusted for 3 covariate

B2B2s3adALPHAtab<-matrix(NA,nsim,18)

B2B2s3adESTMtab<-matrix(NA,nsim,18)

B2B2s3adPOWERtab<-matrix(NA,nsim,18)

B2B2s3adBIAStab<-matrix(NA,nsim,18)

colnames(B2B2s3adALPHAtab)<-colnames(B2B2s3adESTMtab)<-colnames(B2B2s3adPOWERtab)<-colnames(B2B2s3adBIAStab)<-

c("b10","b20","b30","b40","b50","b60","b70","b80","b90","w90","w80","w70","w60","w50","w40","w30","w20","w10")

#constrained on 2 covariate, adjusted for 4 covariate

B2B2s4adALPHAtab<-matrix(NA,nsim,18)

B2B2s4adESTMtab<-matrix(NA,nsim,18)

B2B2s4adPOWERtab<-matrix(NA,nsim,18)

B2B2s4adBIAStab<-matrix(NA,nsim,18)

colnames(B2B2s4adALPHAtab)<-colnames(B2B2s4adESTMtab)<-colnames(B2B2s4adPOWERtab)<-colnames(B2B2s4adBIAStab)<-

c("b10","b20","b30","b40","b50","b60","b70","b80","b90","w90","w80","w70","w60","w50","w40","w30","w20","w10")

#constrained on 3 covariate, adjusted for 1 covariate

B2B3s1adALPHAtab<-matrix(NA,nsim,18)

B2B3s1adESTMtab<-matrix(NA,nsim,18)

B2B3s1adPOWERtab<-matrix(NA,nsim,18)

B2B3s1adBIAStab<-matrix(NA,nsim,18)

colnames(B2B3s1adALPHAtab)<-colnames(B2B3s1adESTMtab)<-colnames(B2B3s1adPOWERtab)<-colnames(B2B3s1adBIAStab)<-

c("b10","b20","b30","b40","b50","b60","b70","b80","b90","w90","w80","w70","w60","w50","w40","w30","w20","w10")

#constrained on 3 covariate, adjusted for 2 covariate

B2B3s2adALPHAtab<-matrix(NA,nsim,18)

B2B3s2adESTMtab<-matrix(NA,nsim,18)

B2B3s2adPOWERtab<-matrix(NA,nsim,18)

B2B3s2adBIAStab<-matrix(NA,nsim,18)

colnames(B2B3s2adALPHAtab)<-colnames(B2B3s2adESTMtab)<-colnames(B2B3s2adPOWERtab)<-colnames(B2B3s2adBIAStab)<-

c("b10","b20","b30","b40","b50","b60","b70","b80","b90","w90","w80","w70","w60","w50","w40","w30","w20","w10")

#constrained on 3 covariate, adjusted for 3 covariate

B2B3s3adALPHAtab<-matrix(NA,nsim,18)

B2B3s3adESTMtab<-matrix(NA,nsim,18)

B2B3s3adPOWERtab<-matrix(NA,nsim,18)

B2B3s3adBIAStab<-matrix(NA,nsim,18)

colnames(B2B3s3adALPHAtab)<-colnames(B2B3s3adESTMtab)<-colnames(B2B3s3adPOWERtab)<-colnames(B2B3s3adBIAStab)<-

c("b10","b20","b30","b40","b50","b60","b70","b80","b90","w90","w80","w70","w60","w50","w40","w30","w20","w10")

#constrained on 3 covariate, adjusted for 4 covariate

B2B3s4adALPHAtab<-matrix(NA,nsim,18)

B2B3s4adESTMtab<-matrix(NA,nsim,18)

B2B3s4adPOWERtab<-matrix(NA,nsim,18)

B2B3s4adBIAStab<-matrix(NA,nsim,18)

colnames(B2B3s4adALPHAtab)<-colnames(B2B3s4adESTMtab)<-colnames(B2B3s4adPOWERtab)<-colnames(B2B3s4adBIAStab)<-

c("b10","b20","b30","b40","b50","b60","b70","b80","b90","w90","w80","w70","w60","w50","w40","w30","w20","w10")

#constrained on 4 covariate, adjusted for 1 covariate

B2B4s1adALPHAtab<-matrix(NA,nsim,18)

B2B4s1adESTMtab<-matrix(NA,nsim,18)

B2B4s1adPOWERtab<-matrix(NA,nsim,18)

B2B4s1adBIAStab<-matrix(NA,nsim,18)

colnames(B2B4s1adALPHAtab)<-colnames(B2B4s1adESTMtab)<-colnames(B2B4s1adPOWERtab)<-colnames(B2B4s1adBIAStab)<-

c("b10","b20","b30","b40","b50","b60","b70","b80","b90","w90","w80","w70","w60","w50","w40","w30","w20","w10")

#constrained on 4 covariate, adjusted for 2 covariate

B2B4s2adALPHAtab<-matrix(NA,nsim,18)

B2B4s2adESTMtab<-matrix(NA,nsim,18)

B2B4s2adPOWERtab<-matrix(NA,nsim,18)

B2B4s2adBIAStab<-matrix(NA,nsim,18)

colnames(B2B4s2adALPHAtab)<-colnames(B2B4s2adESTMtab)<-colnames(B2B4s2adPOWERtab)<-colnames(B2B4s2adBIAStab)<-

c("b10","b20","b30","b40","b50","b60","b70","b80","b90","w90","w80","w70","w60","w50","w40","w30","w20","w10")

#constrained on 4 covariate, adjusted for 3 covariate

B2B4s3adALPHAtab<-matrix(NA,nsim,18)

B2B4s3adESTMtab<-matrix(NA,nsim,18)

B2B4s3adPOWERtab<-matrix(NA,nsim,18)

B2B4s3adBIAStab<-matrix(NA,nsim,18)

colnames(B2B4s3adALPHAtab)<-colnames(B2B4s3adESTMtab)<-colnames(B2B4s3adPOWERtab)<-colnames(B2B4s3adBIAStab)<-

c("b10","b20","b30","b40","b50","b60","b70","b80","b90","w90","w80","w70","w60","w50","w40","w30","w20","w10")

#constrained on 4 covariate, adjusted for 4 covariate

B2B4s4adALPHAtab<-matrix(NA,nsim,18)

B2B4s4adESTMtab<-matrix(NA,nsim,18)

B2B4s4adPOWERtab<-matrix(NA,nsim,18)

B2B4s4adBIAStab<-matrix(NA,nsim,18)

colnames(B2B4s4adALPHAtab)<-colnames(B2B4s4adESTMtab)<-colnames(B2B4s4adPOWERtab)<-colnames(B2B4s4adBIAStab)<-

c("b10","b20","b30","b40","b50","b60","b70","b80","b90","w90","w80","w70","w60","w50","w40","w30","w20","w10")

###

##put matrices in lists, by number of covariates adjusted for in the analysis, for each estimate:

B2s1adALPHAtab_names<-list(B2B1s1adALPHAtab,B2B2s1adALPHAtab,B2B3s1adALPHAtab,B2B4s1adALPHAtab)

B2s2adALPHAtab_names<-list(B2B1s2adALPHAtab,B2B2s2adALPHAtab,B2B3s2adALPHAtab,B2B4s2adALPHAtab)

B2s3adALPHAtab_names<-list(B2B1s3adALPHAtab,B2B2s3adALPHAtab,B2B3s3adALPHAtab,B2B4s3adALPHAtab)

B2s4adALPHAtab_names<-list(B2B1s4adALPHAtab,B2B2s4adALPHAtab,B2B3s4adALPHAtab,B2B4s4adALPHAtab)

B2s1adESTMtab_names<-list(B2B1s1adESTMtab,B2B2s1adESTMtab,B2B3s1adESTMtab,B2B4s1adESTMtab)

B2s2adESTMtab_names<-list(B2B1s2adESTMtab,B2B2s2adESTMtab,B2B3s2adESTMtab,B2B4s2adESTMtab)

B2s3adESTMtab_names<-list(B2B1s3adESTMtab,B2B2s3adESTMtab,B2B3s3adESTMtab,B2B4s3adESTMtab)

B2s4adESTMtab_names<-list(B2B1s4adESTMtab,B2B2s4adESTMtab,B2B3s4adESTMtab,B2B4s4adESTMtab)

B2s1adPOWERtab_names<-list(B2B1s1adPOWERtab,B2B2s1adPOWERtab,B2B3s1adPOWERtab,B2B4s1adPOWERtab)

B2s2adPOWERtab_names<-list(B2B1s2adPOWERtab,B2B2s2adPOWERtab,B2B3s2adPOWERtab,B2B4s2adPOWERtab)

B2s3adPOWERtab_names<-list(B2B1s3adPOWERtab,B2B2s3adPOWERtab,B2B3s3adPOWERtab,B2B4s3adPOWERtab)

B2s4adPOWERtab_names<-list(B2B1s4adPOWERtab,B2B2s4adPOWERtab,B2B3s4adPOWERtab,B2B4s4adPOWERtab)

B2s1adBIAStab_names<-list(B2B1s1adBIAStab,B2B2s1adBIAStab,B2B3s1adBIAStab,B2B4s1adBIAStab)

B2s2adBIAStab_names<-list(B2B1s2adBIAStab,B2B2s2adBIAStab,B2B3s2adBIAStab,B2B4s2adBIAStab)

B2s3adBIAStab_names<-list(B2B1s3adBIAStab,B2B2s3adBIAStab,B2B3s3adBIAStab,B2B4s3adBIAStab)

B2s4adBIAStab_names<-list(B2B1s4adBIAStab,B2B2s4adBIAStab,B2B3s4adBIAStab,B2B4s4adBIAStab)

###

##############

##simulation##

##############

# generate nsim valid simulated trials (removing false data)

i<-0 #set simulation ID

while(i<nsim){

i<-i+1 #identify a new simulation

print(i)

tempi<-i

### NB: to change if changing the no. covariates ###

# generate the binary cluster-level covariates

cl.x1<-rbinom(nClust,1,0.3) #for each cluster, their covariate values

cl.x2<-rbinom(nClust,1,0.3)

cl.x3<-rbinom(nClust,1,0.3)

cl.x4<-rbinom(nClust,1,0.3)

#require min 2 clusters with/without the covariate

testx1=sum(cl.x1)

testx2=sum(cl.x2)

testx3=sum(cl.x3)

testx4=sum(cl.x4)

testV=nClust-2

#redraw x if all clusters have the same value of x

while((testx1<2) | (testx1>testV)){cl.x1<-rbinom(nClust,1,0.3)

testx1=sum(cl.x1)}

while((testx2<2) | (testx2>testV)){cl.x2<-rbinom(nClust,1,0.3)

testx2=sum(cl.x2)}

while((testx3<2) | (testx3>testV)){cl.x3<-rbinom(nClust,1,0.3)

testx3=sum(cl.x3)}

while((testx4<2) | (testx4>testV)){cl.x4<-rbinom(nClust,1,0.3)

testx4=sum(cl.x4)}

#there should now be at least 1 cluster with/without each of the binary covariates

cl.x<-cbind(cl.x1,cl.x2,cl.x3,cl.x4) #clusters x binary covariate values

x1<-rep(cl.x1,each=ClustSize)

x2<-rep(cl.x2,each=ClustSize)

x3<-rep(cl.x3,each=ClustSize)

x4<-rep(cl.x4,each=ClustSize)

x<-cbind(x1,x2,x3,x4)

#put the data together

X1<-cl.x1

X2<-cl.x[,1:2] #binary covarite values are stored for each cluster

X3<-cl.x[,1:3]

X4<-cl.x[,1:4]

XX<-list(X1,X2,X3,X4)

#generate random intercept values (bij's)

alpha_ij=rep(rnorm(nClust,1,lambda*sig_e),each=ClustSize)

#generate the outcome

mu.y<-x%*%gamma+alpha_ij

y0<-rnorm(obs,mu.y,sig_e)

##############################################################

#simulate S randomisation schemes (permutations)

trt_alloc<-matrix(0,S,nClust) #matrix of zeros

for (s in 1:S){

trt<-sample(cl,nClust/2) #sample half of the clusters

trt_alloc[s,trt]<-1 #allocate them to the treatment group

}

trt_alloc<-unique(trt_alloc) # removes duplicate randomisation schemes

R<-dim(trt_alloc)[1] #number of randomisation schemes

print(R)

ctrl_alloc<-1-trt_alloc # complement of randomisation schemes ie indicating control clusters

#so we have a matrix indicating possible allocations of clusters to the

#treatment and the same but indicating the control clusters

################################################################

################## Simple randomisation ########################

for(j in 1:nCC){

# choose a randomisation scheme at random:

rw<-sample(nrow(trt_alloc),size=1) #row in possible schemes

scheme<-trt_alloc[rw,] #extract randomisation scheme

#generate treatment indicator for each observation

t<-rep(scheme,each=ClustSize)

## Type I error ##

# Unadjusted analysis: #

#Define UNADJUSTED model for SR when trt effect=0 (T1E):

fm0<-as.formula(paste0("y0~t"))

#try to fit the model and print any error messages:

fit0<-try(lme(fixed=fm0,random=~1|cluster,control=lmeControl(opt = "optim")),silent=F)

#if an error was created break the sim run and add 1 to the error counter:

if(class(fit0)=="try-error"){errorSR_UN<-errorSR_UN+1;i<-i-1;break}

#otherwise: put the results in the matrix generated earlier:

SRunALPHAtab[i]<-summary(fit0)$tTable["t","p-value"]

SRunESTMtab[i]<-summary(fit0)$tTable["t","Value"]

# Adjusted analysis: #

#Define ADJUSTED model for SR including x1->xj, when trt effect=0 (T1E):

fm0<-as.formula(paste0("y0~t+",paste(paste0("x",1:j),collapse="+")))

#try to fit the model and print any error messages:

fit0<-try(lme(fixed=fm0,random=~1|cluster,control=lmeControl(opt = "optim")),silent=F)

#if an error was created break the sim run and add 1 to the error counter:

if(class(fit0)=="try-error"){errorSR_AD<-errorSR_AD+1;i<-i-1;break}

#otherwise: put the results in the matrix generated earlier:

SRadALPHAtab[i,j]<-summary(fit0)$tTable["t","p-value"]

SRadESTMtab[i,j]<-summary(fit0)$tTable["t","Value"]

## Power ##

#generate the average y for those in the trt and control when trt effect=theta:

mu.pwr<-mu.y+theta*t

#y generated from normal with mean dependent on trt group

y1<-rnorm(obs,mu.pwr,sig_e)

# Unadjusted analysis: #

#define the UNADJUSTED model for SR as above:

fm1<-as.formula(paste0("y1~t"))

#try to fit the model as above

fit1<-try(lme(fixed=fm1,random=~1|cluster,control=lmeControl(opt = "optim")),silent=F)

#if an error occurs as above

if(class(fit1)=="try-error"){errorSR_UN<-errorSR_UN+1;i<-i-1;break}

#otherwise: put the results in the matrix generated earlier:

SRunPOWERtab[i]<-summary(fit1)$tTable["t","p-value"]

SRunBIAStab[i]<-summary(fit1)$tTable["t","Value"]-theta

# Adjusted analysis: #

#define the ADJUSTED model for SR as above:

fm1<-as.formula(paste0("y1~t+",paste(paste0("x",1:j),collapse="+")))

#try to fit the model as above

fit1<-try(lme(fixed=fm1,random=~1|cluster,control=lmeControl(opt = "optim")),silent=F)

#if an error occurs as above

if(class(fit1)=="try-error"){errorSR_AD<-errorSR_AD+1;i<-i-1;break}

#otherwise: put the results in the matrix generated earlier:

SRadPOWERtab[i,j]<-summary(fit1)$tTable["t","p-value"]

SRadBIAStab[i,j]<-summary(fit1)$tTable["t","Value"]-theta

}

if(i<tempi){next}

#################################################################

######## Covariate constrained randomisation: B2 metric #########

# generate treatment allocation for each observation for each randomisation scheme

trt<- matrix(0,R,obs)

for(k in 1:R){

trt[k,]<-rep(trt_alloc[k,],each=ClustSize)

}

#define the cut offs for the candidate set:

q10<-round(0.1*R)

q20<-round(0.2*R)

q30<-round(0.3*R)

q40<-round(0.4*R)

q50<-round(0.5*R)

q60<-round(0.6*R)

q70<-round(0.7*R)

q80<-round(0.8*R)

q90<-round(0.9*R)

#Define the randomisation space

B2<-matrix(NA,R,4) #matrix to store scores for each scheme depending on no. covariates constrained

B2[,1]<-((2/nClust)*(((ctrl_alloc%*%X1)-(trt_alloc%*%X1))/sd(X1)))^2

B2[,2]<-apply(((2/nClust)*(((ctrl_alloc%*%X2)-(trt_alloc%*%X2))/(matrix(apply(X2,2,sd),R,2,byrow=T))))^2,1,sum)

B2[,3]<-apply(((2/nClust)*(((ctrl_alloc%*%X3)-(trt_alloc%*%X3))/(matrix(apply(X3,2,sd),R,3,byrow=T))))^2,1,sum)

B2[,4]<-apply(((2/nClust)*(((ctrl_alloc%*%X4)-(trt_alloc%*%X4))/(matrix(apply(X4,2,sd),R,4,byrow=T))))^2,1,sum)

#

## When balancing on j of the covariates, select a scheme from each of the candidate sets,

## and analyse adjusting for between 0 and j of the covariates that were balanced on:

#####################################################

for(j in 1:4){

## Choose a randomisation scheme at random from the various candidate sets: ##

#best 10%

rw_b10<-sample(order(B2[,j])[1:q10],1) #row in 10% possible schemes with best balance scores

scheme_b10<-trt_alloc[rw_b10,] #extract randomisation scheme

#generate treatment indicator for each observation

t_b10<-rep(scheme_b10,each=ClustSize)

#best 20%

rw_b20<-sample(order(B2[,j])[1:q20],1)

scheme_b20<-trt_alloc[rw_b20,]

t_b20<-rep(scheme_b20,each=ClustSize)

#best 30%

rw_b30<-sample(order(B2[,j])[1:q30],1)

scheme_b30<-trt_alloc[rw_b30,]

t_b30<-rep(scheme_b30,each=ClustSize)

#best 40%

rw_b40<-sample(order(B2[,j])[1:q40],1)

scheme_b40<-trt_alloc[rw_b40,]

t_b40<-rep(scheme_b40,each=ClustSize)

#best 50%

rw_b50<-sample(order(B2[,j])[1:q50],1)

scheme_b50<-trt_alloc[rw_b50,]

t_b50<-rep(scheme_b50,each=ClustSize)

#best 60%

rw_b60<-sample(order(B2[,j])[1:q60],1)

scheme_b60<-trt_alloc[rw_b60,]

t_b60<-rep(scheme_b60,each=ClustSize)

#best 70%

rw_b70<-sample(order(B2[,j])[1:q70],1)

scheme_b70<-trt_alloc[rw_b70,]

t_b70<-rep(scheme_b70,each=ClustSize)

#best 80%

rw_b80<-sample(order(B2[,j])[1:q80],1)

scheme_b80<-trt_alloc[rw_b80,]

t_b80<-rep(scheme_b80,each=ClustSize)

#best 90%

rw_b90<-sample(order(B2[,j])[1:q90],1)

scheme_b90<-trt_alloc[rw_b90,]

t_b90<-rep(scheme_b90,each=ClustSize)

#worst 90%

rw_w90<-sample(order(B2[,j], decreasing=T)[1:q90],1)

scheme_w90<-trt_alloc[rw_w90,]

t_w90<-rep(scheme_w90,each=ClustSize)

#worst 80%

rw_w80<-sample(order(B2[,j], decreasing=T)[1:q80],1)

scheme_w80<-trt_alloc[rw_w80,]

t_w80<-rep(scheme_w80,each=ClustSize)

#worst 70%

rw_w70<-sample(order(B2[,j], decreasing=T)[1:q70],1)

scheme_w70<-trt_alloc[rw_w70,]

t_w70<-rep(scheme_w70,each=ClustSize)

#worst 60%

rw_w60<-sample(order(B2[,j], decreasing=T)[1:q60],1)

scheme_w60<-trt_alloc[rw_w60,]

t_w60<-rep(scheme_w60,each=ClustSize)

#worst 50%

rw_w50<-sample(order(B2[,j], decreasing=T)[1:q50],1)

scheme_w50<-trt_alloc[rw_w50,]

t_w50<-rep(scheme_w50,each=ClustSize)

#worst 40%

rw_w40<-sample(order(B2[,j], decreasing=T)[1:q40],1)

scheme_w40<-trt_alloc[rw_w40,]

t_w40<-rep(scheme_w40,each=ClustSize)

#worst 30%

rw_w30<-sample(order(B2[,j], decreasing=T)[1:q30],1)

scheme_w30<-trt_alloc[rw_w30,]

t_w30<-rep(scheme_w30,each=ClustSize)

#worst 20%

rw_w20<-sample(order(B2[,j], decreasing=T)[1:q20],1)

scheme_w20<-trt_alloc[rw_w20,]

t_w20<-rep(scheme_w20,each=ClustSize)

#worst 10%

rw_w10<-sample(order(B2[,j], decreasing=T)[1:q10],1)

scheme_w10<-trt_alloc[rw_w10,]

t_w10<-rep(scheme_w10,each=ClustSize)

###################################################

## Type I error ##

# Unadjusted analysis: #

#Best 10%

fm0<-as.formula(paste0("y0~t_b10"))

#try to fit the model and print any error messages:

fit0<-try(lme(fixed=fm0,random=~1|cluster,control=lmeControl(opt = "optim")),silent=F)

#if an error was created break the sim run and add 1 to the error counter:

if(class(fit0)=="try-error"){errorB2_UN<-errorB2_UN+1;i<-i-1;break}

#otherwise: put the results in the matrix generated earlier:

B2unALPHAtab_b10[i,j]<-summary(fit0)$tTable["t_b10","p-value"]

B2unESTMtab_b10[i,j]<-summary(fit0)$tTable["t_b10","Value"]

#Best 20%

fm0<-as.formula(paste0("y0~t_b20"))

fit0<-try(lme(fixed=fm0,random=~1|cluster,control=lmeControl(opt = "optim")),silent=F)

if(class(fit0)=="try-error"){errorB2_UN<-errorB2_UN+1;i<-i-1;break}

B2unALPHAtab_b20[i,j]<-summary(fit0)$tTable["t_b20","p-value"]

B2unESTMtab_b20[i,j]<-summary(fit0)$tTable["t_b20","Value"]

#Best 30%

fm0<-as.formula(paste0("y0~t_b30"))

fit0<-try(lme(fixed=fm0,random=~1|cluster,control=lmeControl(opt = "optim")),silent=F)

if(class(fit0)=="try-error"){errorB2_UN<-errorB2_UN+1;i<-i-1;break}

B2unALPHAtab_b30[i,j]<-summary(fit0)$tTable["t_b30","p-value"]

B2unESTMtab_b30[i,j]<-summary(fit0)$tTable["t_b30","Value"]

#Best 40%

fm0<-as.formula(paste0("y0~t_b40"))

fit0<-try(lme(fixed=fm0,random=~1|cluster,control=lmeControl(opt = "optim")),silent=F)

if(class(fit0)=="try-error"){errorB2_UN<-errorB2_UN+1;i<-i-1;break}

B2unALPHAtab_b40[i,j]<-summary(fit0)$tTable["t_b40","p-value"]

B2unESTMtab_b40[i,j]<-summary(fit0)$tTable["t_b40","Value"]

#Best 50%

fm0<-as.formula(paste0("y0~t_b50"))

fit0<-try(lme(fixed=fm0,random=~1|cluster,control=lmeControl(opt = "optim")),silent=F)

if(class(fit0)=="try-error"){errorB2_UN<-errorB2_UN+1;i<-i-1;break}

B2unALPHAtab_b50[i,j]<-summary(fit0)$tTable["t_b50","p-value"]

B2unESTMtab_b50[i,j]<-summary(fit0)$tTable["t_b50","Value"]

#Best 60%

fm0<-as.formula(paste0("y0~t_b60"))

fit0<-try(lme(fixed=fm0,random=~1|cluster,control=lmeControl(opt = "optim")),silent=F)

if(class(fit0)=="try-error"){errorB2_UN<-errorB2_UN+1;i<-i-1;break}

B2unALPHAtab_b60[i,j]<-summary(fit0)$tTable["t_b60","p-value"]

B2unESTMtab_b60[i,j]<-summary(fit0)$tTable["t_b60","Value"]

#Best 70%

fm0<-as.formula(paste0("y0~t_b70"))

fit0<-try(lme(fixed=fm0,random=~1|cluster,control=lmeControl(opt = "optim")),silent=F)

if(class(fit0)=="try-error"){errorB2_UN<-errorB2_UN+1;i<-i-1;break}

B2unALPHAtab_b70[i,j]<-summary(fit0)$tTable["t_b70","p-value"]

B2unESTMtab_b70[i,j]<-summary(fit0)$tTable["t_b70","Value"]

#Best 80%")

fm0<-as.formula(paste0("y0~t_b80"))

fit0<-try(lme(fixed=fm0,random=~1|cluster,control=lmeControl(opt = "optim")),silent=F)

if(class(fit0)=="try-error"){errorB2_UN<-errorB2_UN+1;i<-i-1;break}

B2unALPHAtab_b80[i,j]<-summary(fit0)$tTable["t_b80","p-value"]

B2unESTMtab_b80[i,j]<-summary(fit0)$tTable["t_b80","Value"]

#Best 90%")

fm0<-as.formula(paste0("y0~t_b90"))

fit0<-try(lme(fixed=fm0,random=~1|cluster,control=lmeControl(opt = "optim")),silent=F)

if(class(fit0)=="try-error"){errorB2_UN<-errorB2_UN+1;i<-i-1;break}

B2unALPHAtab_b90[i,j]<-summary(fit0)$tTable["t_b90","p-value"]

B2unESTMtab_b90[i,j]<-summary(fit0)$tTable["t_b90","Value"]

#Worst 10%")

fm0<-as.formula(paste0("y0~t_w10"))

fit0<-try(lme(fixed=fm0,random=~1|cluster,control=lmeControl(opt = "optim")),silent=F)

if(class(fit0)=="try-error"){errorB2_UN<-errorB2_UN+1;i<-i-1;break}

B2unALPHAtab_w10[i,j]<-summary(fit0)$tTable["t_w10","p-value"]

B2unESTMtab_w10[i,j]<-summary(fit0)$tTable["t_w10","Value"]

#Worst 20%")

fm0<-as.formula(paste0("y0~t_w20"))

fit0<-try(lme(fixed=fm0,random=~1|cluster,control=lmeControl(opt = "optim")),silent=F)

if(class(fit0)=="try-error"){errorB2_UN<-errorB2_UN+1;i<-i-1;break}

B2unALPHAtab_w20[i,j]<-summary(fit0)$tTable["t_w20","p-value"]

B2unESTMtab_w20[i,j]<-summary(fit0)$tTable["t_w20","Value"]

#Worst 30%")

fm0<-as.formula(paste0("y0~t_w30"))

fit0<-try(lme(fixed=fm0,random=~1|cluster,control=lmeControl(opt = "optim")),silent=F)

if(class(fit0)=="try-error"){errorB2_UN<-errorB2_UN+1;i<-i-1;break}

B2unALPHAtab_w30[i,j]<-summary(fit0)$tTable["t_w30","p-value"]

B2unESTMtab_w30[i,j]<-summary(fit0)$tTable["t_w30","Value"]

#Worst 40%")

fm0<-as.formula(paste0("y0~t_w40"))

fit0<-try(lme(fixed=fm0,random=~1|cluster,control=lmeControl(opt = "optim")),silent=F)

if(class(fit0)=="try-error"){errorB2_UN<-errorB2_UN+1;i<-i-1;break}

B2unALPHAtab_w40[i,j]<-summary(fit0)$tTable["t_w40","p-value"]

B2unESTMtab_w40[i,j]<-summary(fit0)$tTable["t_w40","Value"]

#Worst 50%")

fm0<-as.formula(paste0("y0~t_w50"))

fit0<-try(lme(fixed=fm0,random=~1|cluster,control=lmeControl(opt = "optim")),silent=F)

if(class(fit0)=="try-error"){errorB2_UN<-errorB2_UN+1;i<-i-1;break}

B2unALPHAtab_w50[i,j]<-summary(fit0)$tTable["t_w50","p-value"]

B2unESTMtab_w50[i,j]<-summary(fit0)$tTable["t_w50","Value"]

#Worst 60%")

fm0<-as.formula(paste0("y0~t_w60"))

fit0<-try(lme(fixed=fm0,random=~1|cluster,control=lmeControl(opt = "optim")),silent=F)

if(class(fit0)=="try-error"){errorB2_UN<-errorB2_UN+1;i<-i-1;break}

B2unALPHAtab_w60[i,j]<-summary(fit0)$tTable["t_w60","p-value"]

B2unESTMtab_w60[i,j]<-summary(fit0)$tTable["t_w60","Value"]

#Worst 70%")

fm0<-as.formula(paste0("y0~t_w70"))

fit0<-try(lme(fixed=fm0,random=~1|cluster,control=lmeControl(opt = "optim")),silent=F)

if(class(fit0)=="try-error"){errorB2_UN<-errorB2_UN+1;i<-i-1;break}

B2unALPHAtab_w70[i,j]<-summary(fit0)$tTable["t_w70","p-value"]

B2unESTMtab_w70[i,j]<-summary(fit0)$tTable["t_w70","Value"]

#Worst 80%")

fm0<-as.formula(paste0("y0~t_w80"))

fit0<-try(lme(fixed=fm0,random=~1|cluster,control=lmeControl(opt = "optim")),silent=F)

if(class(fit0)=="try-error"){errorB2_UN<-errorB2_UN+1;i<-i-1;break}

B2unALPHAtab_w80[i,j]<-summary(fit0)$tTable["t_w80","p-value"]

B2unESTMtab_w80[i,j]<-summary(fit0)$tTable["t_w80","Value"]

#Worst 90%")

fm0<-as.formula(paste0("y0~t_w90"))

fit0<-try(lme(fixed=fm0,random=~1|cluster,control=lmeControl(opt = "optim")),silent=F)

if(class(fit0)=="try-error"){errorB2_UN<-errorB2_UN+1;i<-i-1;break}

B2unALPHAtab_w90[i,j]<-summary(fit0)$tTable["t_w90","p-value"]

B2unESTMtab_w90[i,j]<-summary(fit0)$tTable["t_w90","Value"]

####

# Adjusted analysis: (balanced on j covariates) #

#Best 10%

#x1

fm0<-as.formula(paste0("y0~t_b10+x1"))

fit0<-try(lme(fixed=fm0,random=~1|cluster,control=lmeControl(opt = "optim")),silent=F)

if(class(fit0)=="try-error"){errorB2_AD<-errorB2_AD+1;i<-i-1;break}

B2s1adALPHAtab_names[[j]][i,1]<-summary(fit0)$tTable["t_b10","p-value"]

B2s1adESTMtab_names[[j]][i,1]<-summary(fit0)$tTable["t_b10","Value"]

#x2

fm0<-as.formula(paste0("y0~t_b10+x1+x2"))

fit0<-try(lme(fixed=fm0,random=~1|cluster,control=lmeControl(opt = "optim")),silent=F)

if(class(fit0)=="try-error"){errorB2_AD<-errorB2_AD+1;i<-i-1;break}

B2s2adALPHAtab_names[[j]][i,1]<-summary(fit0)$tTable["t_b10","p-value"]

B2s2adESTMtab_names[[j]][i,1]<-summary(fit0)$tTable["t_b10","Value"]

#x3

fm0<-as.formula(paste0("y0~t_b10+x1+x2+x3"))

fit0<-try(lme(fixed=fm0,random=~1|cluster,control=lmeControl(opt = "optim")),silent=F)

if(class(fit0)=="try-error"){errorB2_AD<-errorB2_AD+1;i<-i-1;break}

B2s3adALPHAtab_names[[j]][i,1]<-summary(fit0)$tTable["t_b10","p-value"]

B2s3adESTMtab_names[[j]][i,1]<-summary(fit0)$tTable["t_b10","Value"]

#x4

fm0<-as.formula(paste0("y0~t_b10+x1+x2+x3+x4"))

fit0<-try(lme(fixed=fm0,random=~1|cluster,control=lmeControl(opt = "optim")),silent=F)

if(class(fit0)=="try-error"){errorB2_AD<-errorB2_AD+1;i<-i-1;break}

B2s4adALPHAtab_names[[j]][i,1]<-summary(fit0)$tTable["t_b10","p-value"]

B2s4adESTMtab_names[[j]][i,1]<-summary(fit0)$tTable["t_b10","Value"]

#Best 20%

#x1

fm0<-as.formula(paste0("y0~t_b20+x1"))

fit0<-try(lme(fixed=fm0,random=~1|cluster,control=lmeControl(opt = "optim")),silent=F)

if(class(fit0)=="try-error"){errorB2_AD<-errorB2_AD+1;i<-i-1;break}

B2s1adALPHAtab_names[[j]][i,2]<-summary(fit0)$tTable["t_b20","p-value"]

B2s1adESTMtab_names[[j]][i,2]<-summary(fit0)$tTable["t_b20","Value"]

#x2

fm0<-as.formula(paste0("y0~t_b20+x1+x2"))

fit0<-try(lme(fixed=fm0,random=~1|cluster,control=lmeControl(opt = "optim")),silent=F)

if(class(fit0)=="try-error"){errorB2_AD<-errorB2_AD+1;i<-i-1;break}

B2s2adALPHAtab_names[[j]][i,2]<-summary(fit0)$tTable["t_b20","p-value"]

B2s2adESTMtab_names[[j]][i,2]<-summary(fit0)$tTable["t_b20","Value"]

#x3

fm0<-as.formula(paste0("y0~t_b20+x1+x2+x3"))

fit0<-try(lme(fixed=fm0,random=~1|cluster,control=lmeControl(opt = "optim")),silent=F)

if(class(fit0)=="try-error"){errorB2_AD<-errorB2_AD+1;i<-i-1;break}

B2s3adALPHAtab_names[[j]][i,2]<-summary(fit0)$tTable["t_b20","p-value"]

B2s3adESTMtab_names[[j]][i,2]<-summary(fit0)$tTable["t_b20","Value"]

#x4

fm0<-as.formula(paste0("y0~t_b20+x1+x2+x3+x4"))

fit0<-try(lme(fixed=fm0,random=~1|cluster,control=lmeControl(opt = "optim")),silent=F)

if(class(fit0)=="try-error"){errorB2_AD<-errorB2_AD+1;i<-i-1;break}

B2s4adALPHAtab_names[[j]][i,2]<-summary(fit0)$tTable["t_b20","p-value"]

B2s4adESTMtab_names[[j]][i,2]<-summary(fit0)$tTable["t_b20","Value"]

#Best 30%

#x1

fm0<-as.formula(paste0("y0~t_b30+x1"))

fit0<-try(lme(fixed=fm0,random=~1|cluster,control=lmeControl(opt = "optim")),silent=F)

if(class(fit0)=="try-error"){errorB2_AD<-errorB2_AD+1;i<-i-1;break}

B2s1adALPHAtab_names[[j]][i,3]<-summary(fit0)$tTable["t_b30","p-value"]

B2s1adESTMtab_names[[j]][i,3]<-summary(fit0)$tTable["t_b30","Value"]

#x2

fm0<-as.formula(paste0("y0~t_b30+x1+x2"))

fit0<-try(lme(fixed=fm0,random=~1|cluster,control=lmeControl(opt = "optim")),silent=F)

if(class(fit0)=="try-error"){errorB2_AD<-errorB2_AD+1;i<-i-1;break}

B2s2adALPHAtab_names[[j]][i,3]<-summary(fit0)$tTable["t_b30","p-value"]

B2s2adESTMtab_names[[j]][i,3]<-summary(fit0)$tTable["t_b30","Value"]

#x3

fm0<-as.formula(paste0("y0~t_b30+x1+x2+x3"))

fit0<-try(lme(fixed=fm0,random=~1|cluster,control=lmeControl(opt = "optim")),silent=F)

if(class(fit0)=="try-error"){errorB2_AD<-errorB2_AD+1;i<-i-1;break}

B2s3adALPHAtab_names[[j]][i,3]<-summary(fit0)$tTable["t_b30","p-value"]

B2s3adESTMtab_names[[j]][i,3]<-summary(fit0)$tTable["t_b30","Value"]

#x4

fm0<-as.formula(paste0("y0~t_b30+x1+x2+x3+x4"))

fit0<-try(lme(fixed=fm0,random=~1|cluster,control=lmeControl(opt = "optim")),silent=F)

if(class(fit0)=="try-error"){errorB2_AD<-errorB2_AD+1;i<-i-1;break}

B2s4adALPHAtab_names[[j]][i,3]<-summary(fit0)$tTable["t_b30","p-value"]

B2s4adESTMtab_names[[j]][i,3]<-summary(fit0)$tTable["t_b30","Value"]

#Best 40%

#x1

fm0<-as.formula(paste0("y0~t_b40+x1"))

fit0<-try(lme(fixed=fm0,random=~1|cluster,control=lmeControl(opt = "optim")),silent=F)

if(class(fit0)=="try-error"){errorB2_AD<-errorB2_AD+1;i<-i-1;break}

B2s1adALPHAtab_names[[j]][i,4]<-summary(fit0)$tTable["t_b40","p-value"]

B2s1adESTMtab_names[[j]][i,4]<-summary(fit0)$tTable["t_b40","Value"]

#x2

fm0<-as.formula(paste0("y0~t_b40+x1+x2"))

fit0<-try(lme(fixed=fm0,random=~1|cluster,control=lmeControl(opt = "optim")),silent=F)

if(class(fit0)=="try-error"){errorB2_AD<-errorB2_AD+1;i<-i-1;break}

B2s2adALPHAtab_names[[j]][i,4]<-summary(fit0)$tTable["t_b40","p-value"]

B2s2adESTMtab_names[[j]][i,4]<-summary(fit0)$tTable["t_b40","Value"]

#x3

fm0<-as.formula(paste0("y0~t_b40+x1+x2+x3"))

fit0<-try(lme(fixed=fm0,random=~1|cluster,control=lmeControl(opt = "optim")),silent=F)

if(class(fit0)=="try-error"){errorB2_AD<-errorB2_AD+1;i<-i-1;break}

B2s3adALPHAtab_names[[j]][i,4]<-summary(fit0)$tTable["t_b40","p-value"]

B2s3adESTMtab_names[[j]][i,4]<-summary(fit0)$tTable["t_b40","Value"]

#x4

fm0<-as.formula(paste0("y0~t_b40+x1+x2+x3+x4"))

fit0<-try(lme(fixed=fm0,random=~1|cluster,control=lmeControl(opt = "optim")),silent=F)

if(class(fit0)=="try-error"){errorB2_AD<-errorB2_AD+1;i<-i-1;break}

B2s4adALPHAtab_names[[j]][i,4]<-summary(fit0)$tTable["t_b40","p-value"]

B2s4adESTMtab_names[[j]][i,4]<-summary(fit0)$tTable["t_b40","Value"]

#Best 50%

#x1

fm0<-as.formula(paste0("y0~t_b50+x1"))

fit0<-try(lme(fixed=fm0,random=~1|cluster,control=lmeControl(opt = "optim")),silent=F)

if(class(fit0)=="try-error"){errorB2_AD<-errorB2_AD+1;i<-i-1;break}

B2s1adALPHAtab_names[[j]][i,5]<-summary(fit0)$tTable["t_b50","p-value"]

B2s1adESTMtab_names[[j]][i,5]<-summary(fit0)$tTable["t_b50","Value"]

#x2

fm0<-as.formula(paste0("y0~t_b50+x1+x2"))

fit0<-try(lme(fixed=fm0,random=~1|cluster,control=lmeControl(opt = "optim")),silent=F)

if(class(fit0)=="try-error"){errorB2_AD<-errorB2_AD+1;i<-i-1;break}

B2s2adALPHAtab_names[[j]][i,5]<-summary(fit0)$tTable["t_b50","p-value"]

B2s2adESTMtab_names[[j]][i,5]<-summary(fit0)$tTable["t_b50","Value"]

#x3

fm0<-as.formula(paste0("y0~t_b50+x1+x2+x3"))

fit0<-try(lme(fixed=fm0,random=~1|cluster,control=lmeControl(opt = "optim")),silent=F)

if(class(fit0)=="try-error"){errorB2_AD<-errorB2_AD+1;i<-i-1;break}

B2s3adALPHAtab_names[[j]][i,5]<-summary(fit0)$tTable["t_b50","p-value"]

B2s3adESTMtab_names[[j]][i,5]<-summary(fit0)$tTable["t_b50","Value"]

#x4

fm0<-as.formula(paste0("y0~t_b50+x1+x2+x3+x4"))

fit0<-try(lme(fixed=fm0,random=~1|cluster,control=lmeControl(opt = "optim")),silent=F)

if(class(fit0)=="try-error"){errorB2_AD<-errorB2_AD+1;i<-i-1;break}

B2s4adALPHAtab_names[[j]][i,5]<-summary(fit0)$tTable["t_b50","p-value"]

B2s4adESTMtab_names[[j]][i,5]<-summary(fit0)$tTable["t_b50","Value"]

#Best 60%

#x1

fm0<-as.formula(paste0("y0~t_b60+x1"))

fit0<-try(lme(fixed=fm0,random=~1|cluster,control=lmeControl(opt = "optim")),silent=F)

if(class(fit0)=="try-error"){errorB2_AD<-errorB2_AD+1;i<-i-1;break}

B2s1adALPHAtab_names[[j]][i,6]<-summary(fit0)$tTable["t_b60","p-value"]

B2s1adESTMtab_names[[j]][i,6]<-summary(fit0)$tTable["t_b60","Value"]

#x2

fm0<-as.formula(paste0("y0~t_b60+x1+x2"))

fit0<-try(lme(fixed=fm0,random=~1|cluster,control=lmeControl(opt = "optim")),silent=F)

if(class(fit0)=="try-error"){errorB2_AD<-errorB2_AD+1;i<-i-1;break}

B2s2adALPHAtab_names[[j]][i,6]<-summary(fit0)$tTable["t_b60","p-value"]

B2s2adESTMtab_names[[j]][i,6]<-summary(fit0)$tTable["t_b60","Value"]

#x3

fm0<-as.formula(paste0("y0~t_b60+x1+x2+x3"))

fit0<-try(lme(fixed=fm0,random=~1|cluster,control=lmeControl(opt = "optim")),silent=F)

if(class(fit0)=="try-error"){errorB2_AD<-errorB2_AD+1;i<-i-1;break}

B2s3adALPHAtab_names[[j]][i,6]<-summary(fit0)$tTable["t_b60","p-value"]

B2s3adESTMtab_names[[j]][i,6]<-summary(fit0)$tTable["t_b60","Value"]

#x4

fm0<-as.formula(paste0("y0~t_b60+x1+x2+x3+x4"))

fit0<-try(lme(fixed=fm0,random=~1|cluster,control=lmeControl(opt = "optim")),silent=F)

if(class(fit0)=="try-error"){errorB2_AD<-errorB2_AD+1;i<-i-1;break}

B2s4adALPHAtab_names[[j]][i,6]<-summary(fit0)$tTable["t_b60","p-value"]

B2s4adESTMtab_names[[j]][i,6]<-summary(fit0)$tTable["t_b60","Value"]

#Best 70%

#x1

fm0<-as.formula(paste0("y0~t_b70+x1"))

fit0<-try(lme(fixed=fm0,random=~1|cluster,control=lmeControl(opt = "optim")),silent=F)

if(class(fit0)=="try-error"){errorB2_AD<-errorB2_AD+1;i<-i-1;break}

B2s1adALPHAtab_names[[j]][i,7]<-summary(fit0)$tTable["t_b70","p-value"]

B2s1adESTMtab_names[[j]][i,7]<-summary(fit0)$tTable["t_b70","Value"]

#x2

fm0<-as.formula(paste0("y0~t_b70+x1+x2"))

fit0<-try(lme(fixed=fm0,random=~1|cluster,control=lmeControl(opt = "optim")),silent=F)

if(class(fit0)=="try-error"){errorB2_AD<-errorB2_AD+1;i<-i-1;break}

B2s2adALPHAtab_names[[j]][i,7]<-summary(fit0)$tTable["t_b70","p-value"]

B2s2adESTMtab_names[[j]][i,7]<-summary(fit0)$tTable["t_b70","Value"]

#x3

fm0<-as.formula(paste0("y0~t_b70+x1+x2+x3"))

fit0<-try(lme(fixed=fm0,random=~1|cluster,control=lmeControl(opt = "optim")),silent=F)

if(class(fit0)=="try-error"){errorB2_AD<-errorB2_AD+1;i<-i-1;break}

B2s3adALPHAtab_names[[j]][i,7]<-summary(fit0)$tTable["t_b70","p-value"]

B2s3adESTMtab_names[[j]][i,7]<-summary(fit0)$tTable["t_b70","Value"]

#x4

fm0<-as.formula(paste0("y0~t_b70+x1+x2+x3+x4"))

fit0<-try(lme(fixed=fm0,random=~1|cluster,control=lmeControl(opt = "optim")),silent=F)

if(class(fit0)=="try-error"){errorB2_AD<-errorB2_AD+1;i<-i-1;break}

B2s4adALPHAtab_names[[j]][i,7]<-summary(fit0)$tTable["t_b70","p-value"]

B2s4adESTMtab_names[[j]][i,7]<-summary(fit0)$tTable["t_b70","Value"]

#Best 80%

#x1

fm0<-as.formula(paste0("y0~t_b80+x1"))

fit0<-try(lme(fixed=fm0,random=~1|cluster,control=lmeControl(opt = "optim")),silent=F)

if(class(fit0)=="try-error"){errorB2_AD<-errorB2_AD+1;i<-i-1;break}

B2s1adALPHAtab_names[[j]][i,8]<-summary(fit0)$tTable["t_b80","p-value"]

B2s1adESTMtab_names[[j]][i,8]<-summary(fit0)$tTable["t_b80","Value"]

#x2

fm0<-as.formula(paste0("y0~t_b80+x1+x2"))

fit0<-try(lme(fixed=fm0,random=~1|cluster,control=lmeControl(opt = "optim")),silent=F)

if(class(fit0)=="try-error"){errorB2_AD<-errorB2_AD+1;i<-i-1;break}

B2s2adALPHAtab_names[[j]][i,8]<-summary(fit0)$tTable["t_b80","p-value"]

B2s2adESTMtab_names[[j]][i,8]<-summary(fit0)$tTable["t_b80","Value"]

#x3

fm0<-as.formula(paste0("y0~t_b80+x1+x2+x3"))

fit0<-try(lme(fixed=fm0,random=~1|cluster,control=lmeControl(opt = "optim")),silent=F)

if(class(fit0)=="try-error"){errorB2_AD<-errorB2_AD+1;i<-i-1;break}

B2s3adALPHAtab_names[[j]][i,8]<-summary(fit0)$tTable["t_b80","p-value"]

B2s3adESTMtab_names[[j]][i,8]<-summary(fit0)$tTable["t_b80","Value"]

#x4

fm0<-as.formula(paste0("y0~t_b80+x1+x2+x3+x4"))

fit0<-try(lme(fixed=fm0,random=~1|cluster,control=lmeControl(opt = "optim")),silent=F)

if(class(fit0)=="try-error"){errorB2_AD<-errorB2_AD+1;i<-i-1;break}

B2s4adALPHAtab_names[[j]][i,8]<-summary(fit0)$tTable["t_b80","p-value"]

B2s4adESTMtab_names[[j]][i,8]<-summary(fit0)$tTable["t_b80","Value"]

#Best 90%

#x1

fm0<-as.formula(paste0("y0~t_b90+x1"))

fit0<-try(lme(fixed=fm0,random=~1|cluster,control=lmeControl(opt = "optim")),silent=F)

if(class(fit0)=="try-error"){errorB2_AD<-errorB2_AD+1;i<-i-1;break}

B2s1adALPHAtab_names[[j]][i,9]<-summary(fit0)$tTable["t_b90","p-value"]

B2s1adESTMtab_names[[j]][i,9]<-summary(fit0)$tTable["t_b90","Value"]

#x2

fm0<-as.formula(paste0("y0~t_b90+x1+x2"))

fit0<-try(lme(fixed=fm0,random=~1|cluster,control=lmeControl(opt = "optim")),silent=F)

if(class(fit0)=="try-error"){errorB2_AD<-errorB2_AD+1;i<-i-1;break}

B2s2adALPHAtab_names[[j]][i,9]<-summary(fit0)$tTable["t_b90","p-value"]

B2s2adESTMtab_names[[j]][i,9]<-summary(fit0)$tTable["t_b90","Value"]

#x3

fm0<-as.formula(paste0("y0~t_b90+x1+x2+x3"))

fit0<-try(lme(fixed=fm0,random=~1|cluster,control=lmeControl(opt = "optim")),silent=F)

if(class(fit0)=="try-error"){errorB2_AD<-errorB2_AD+1;i<-i-1;break}

B2s3adALPHAtab_names[[j]][i,9]<-summary(fit0)$tTable["t_b90","p-value"]

B2s3adESTMtab_names[[j]][i,9]<-summary(fit0)$tTable["t_b90","Value"]

#x4

fm0<-as.formula(paste0("y0~t_b90+x1+x2+x3+x4"))

fit0<-try(lme(fixed=fm0,random=~1|cluster,control=lmeControl(opt = "optim")),silent=F)

if(class(fit0)=="try-error"){errorB2_AD<-errorB2_AD+1;i<-i-1;break}

B2s4adALPHAtab_names[[j]][i,9]<-summary(fit0)$tTable["t_b90","p-value"]

B2s4adESTMtab_names[[j]][i,9]<-summary(fit0)$tTable["t_b90","Value"]

#Worst 90%

#x1

fm0<-as.formula(paste0("y0~t_w90+x1"))

fit0<-try(lme(fixed=fm0,random=~1|cluster,control=lmeControl(opt = "optim")),silent=F)

if(class(fit0)=="try-error"){errorB2_AD<-errorB2_AD+1;i<-i-1;break}

B2s1adALPHAtab_names[[j]][i,10]<-summary(fit0)$tTable["t_w90","p-value"]

B2s1adESTMtab_names[[j]][i,10]<-summary(fit0)$tTable["t_w90","Value"]

#x2

fm0<-as.formula(paste0("y0~t_w90+x1+x2"))

fit0<-try(lme(fixed=fm0,random=~1|cluster,control=lmeControl(opt = "optim")),silent=F)

if(class(fit0)=="try-error"){errorB2_AD<-errorB2_AD+1;i<-i-1;break}

B2s2adALPHAtab_names[[j]][i,10]<-summary(fit0)$tTable["t_w90","p-value"]

B2s2adESTMtab_names[[j]][i,10]<-summary(fit0)$tTable["t_w90","Value"]

#x3

fm0<-as.formula(paste0("y0~t_w90+x1+x2+x3"))

fit0<-try(lme(fixed=fm0,random=~1|cluster,control=lmeControl(opt = "optim")),silent=F)

if(class(fit0)=="try-error"){errorB2_AD<-errorB2_AD+1;i<-i-1;break}

B2s3adALPHAtab_names[[j]][i,10]<-summary(fit0)$tTable["t_w90","p-value"]

B2s3adESTMtab_names[[j]][i,10]<-summary(fit0)$tTable["t_w90","Value"]

#x4

fm0<-as.formula(paste0("y0~t_w90+x1+x2+x3+x4"))

fit0<-try(lme(fixed=fm0,random=~1|cluster,control=lmeControl(opt = "optim")),silent=F)

if(class(fit0)=="try-error"){errorB2_AD<-errorB2_AD+1;i<-i-1;break}

B2s4adALPHAtab_names[[j]][i,10]<-summary(fit0)$tTable["t_w90","p-value"]

B2s4adESTMtab_names[[j]][i,10]<-summary(fit0)$tTable["t_w90","Value"]

#Worst 80%

#x1

fm0<-as.formula(paste0("y0~t_w80+x1"))

fit0<-try(lme(fixed=fm0,random=~1|cluster,control=lmeControl(opt = "optim")),silent=F)

if(class(fit0)=="try-error"){errorB2_AD<-errorB2_AD+1;i<-i-1;break}

B2s1adALPHAtab_names[[j]][i,11]<-summary(fit0)$tTable["t_w80","p-value"]

B2s1adESTMtab_names[[j]][i,11]<-summary(fit0)$tTable["t_w80","Value"]

#x2

fm0<-as.formula(paste0("y0~t_w80+x1+x2"))

fit0<-try(lme(fixed=fm0,random=~1|cluster,control=lmeControl(opt = "optim")),silent=F)

if(class(fit0)=="try-error"){errorB2_AD<-errorB2_AD+1;i<-i-1;break}

B2s2adALPHAtab_names[[j]][i,11]<-summary(fit0)$tTable["t_w80","p-value"]

B2s2adESTMtab_names[[j]][i,11]<-summary(fit0)$tTable["t_w80","Value"]

#x3

fm0<-as.formula(paste0("y0~t_w80+x1+x2+x3"))

fit0<-try(lme(fixed=fm0,random=~1|cluster,control=lmeControl(opt = "optim")),silent=F)

if(class(fit0)=="try-error"){errorB2_AD<-errorB2_AD+1;i<-i-1;break}

B2s3adALPHAtab_names[[j]][i,11]<-summary(fit0)$tTable["t_w80","p-value"]

B2s3adESTMtab_names[[j]][i,11]<-summary(fit0)$tTable["t_w80","Value"]

#x4

fm0<-as.formula(paste0("y0~t_w80+x1+x2+x3+x4"))

fit0<-try(lme(fixed=fm0,random=~1|cluster,control=lmeControl(opt = "optim")),silent=F)

if(class(fit0)=="try-error"){errorB2_AD<-errorB2_AD+1;i<-i-1;break}

B2s4adALPHAtab_names[[j]][i,11]<-summary(fit0)$tTable["t_w80","p-value"]

B2s4adESTMtab_names[[j]][i,11]<-summary(fit0)$tTable["t_w80","Value"]

#Worst 70%

#x1

fm0<-as.formula(paste0("y0~t_w70+x1"))

fit0<-try(lme(fixed=fm0,random=~1|cluster,control=lmeControl(opt = "optim")),silent=F)

if(class(fit0)=="try-error"){errorB2_AD<-errorB2_AD+1;i<-i-1;break}

B2s1adALPHAtab_names[[j]][i,12]<-summary(fit0)$tTable["t_w70","p-value"]

B2s1adESTMtab_names[[j]][i,12]<-summary(fit0)$tTable["t_w70","Value"]

#x2

fm0<-as.formula(paste0("y0~t_w70+x1+x2"))

fit0<-try(lme(fixed=fm0,random=~1|cluster,control=lmeControl(opt = "optim")),silent=F)

if(class(fit0)=="try-error"){errorB2_AD<-errorB2_AD+1;i<-i-1;break}

B2s2adALPHAtab_names[[j]][i,12]<-summary(fit0)$tTable["t_w70","p-value"]

B2s2adESTMtab_names[[j]][i,12]<-summary(fit0)$tTable["t_w70","Value"]

#x3

fm0<-as.formula(paste0("y0~t_w70+x1+x2+x3"))

fit0<-try(lme(fixed=fm0,random=~1|cluster,control=lmeControl(opt = "optim")),silent=F)

if(class(fit0)=="try-error"){errorB2_AD<-errorB2_AD+1;i<-i-1;break}

B2s3adALPHAtab_names[[j]][i,12]<-summary(fit0)$tTable["t_w70","p-value"]

B2s3adESTMtab_names[[j]][i,12]<-summary(fit0)$tTable["t_w70","Value"]

#x4

fm0<-as.formula(paste0("y0~t_w70+x1+x2+x3+x4"))

fit0<-try(lme(fixed=fm0,random=~1|cluster,control=lmeControl(opt = "optim")),silent=F)

if(class(fit0)=="try-error"){errorB2_AD<-errorB2_AD+1;i<-i-1;break}

B2s4adALPHAtab_names[[j]][i,12]<-summary(fit0)$tTable["t_w70","p-value"]

B2s4adESTMtab_names[[j]][i,12]<-summary(fit0)$tTable["t_w70","Value"]

#Worst 60%

#x1

fm0<-as.formula(paste0("y0~t_w60+x1"))

fit0<-try(lme(fixed=fm0,random=~1|cluster,control=lmeControl(opt = "optim")),silent=F)

if(class(fit0)=="try-error"){errorB2_AD<-errorB2_AD+1;i<-i-1;break}

B2s1adALPHAtab_names[[j]][i,13]<-summary(fit0)$tTable["t_w60","p-value"]

B2s1adESTMtab_names[[j]][i,13]<-summary(fit0)$tTable["t_w60","Value"]

#x2

fm0<-as.formula(paste0("y0~t_w60+x1+x2"))

fit0<-try(lme(fixed=fm0,random=~1|cluster,control=lmeControl(opt = "optim")),silent=F)

if(class(fit0)=="try-error"){errorB2_AD<-errorB2_AD+1;i<-i-1;break}

B2s2adALPHAtab_names[[j]][i,13]<-summary(fit0)$tTable["t_w60","p-value"]

B2s2adESTMtab_names[[j]][i,13]<-summary(fit0)$tTable["t_w60","Value"]

#x3

fm0<-as.formula(paste0("y0~t_w60+x1+x2+x3"))

fit0<-try(lme(fixed=fm0,random=~1|cluster,control=lmeControl(opt = "optim")),silent=F)

if(class(fit0)=="try-error"){errorB2_AD<-errorB2_AD+1;i<-i-1;break}

B2s3adALPHAtab_names[[j]][i,13]<-summary(fit0)$tTable["t_w60","p-value"]

B2s3adESTMtab_names[[j]][i,13]<-summary(fit0)$tTable["t_w60","Value"]

#x4

fm0<-as.formula(paste0("y0~t_w60+x1+x2+x3+x4"))

fit0<-try(lme(fixed=fm0,random=~1|cluster,control=lmeControl(opt = "optim")),silent=F)

if(class(fit0)=="try-error"){errorB2_AD<-errorB2_AD+1;i<-i-1;break}

B2s4adALPHAtab_names[[j]][i,13]<-summary(fit0)$tTable["t_w60","p-value"]

B2s4adESTMtab_names[[j]][i,13]<-summary(fit0)$tTable["t_w60","Value"]

#Worst 50%

#x1

fm0<-as.formula(paste0("y0~t_w50+x1"))

fit0<-try(lme(fixed=fm0,random=~1|cluster,control=lmeControl(opt = "optim")),silent=F)

if(class(fit0)=="try-error"){errorB2_AD<-errorB2_AD+1;i<-i-1;break}

B2s1adALPHAtab_names[[j]][i,14]<-summary(fit0)$tTable["t_w50","p-value"]

B2s1adESTMtab_names[[j]][i,14]<-summary(fit0)$tTable["t_w50","Value"]

#x2

fm0<-as.formula(paste0("y0~t_w50+x1+x2"))

fit0<-try(lme(fixed=fm0,random=~1|cluster,control=lmeControl(opt = "optim")),silent=F)

if(class(fit0)=="try-error"){errorB2_AD<-errorB2_AD+1;i<-i-1;break}

B2s2adALPHAtab_names[[j]][i,14]<-summary(fit0)$tTable["t_w50","p-value"]

B2s2adESTMtab_names[[j]][i,14]<-summary(fit0)$tTable["t_w50","Value"]

#x3

fm0<-as.formula(paste0("y0~t_w50+x1+x2+x3"))

fit0<-try(lme(fixed=fm0,random=~1|cluster,control=lmeControl(opt = "optim")),silent=F)

if(class(fit0)=="try-error"){errorB2_AD<-errorB2_AD+1;i<-i-1;break}

B2s3adALPHAtab_names[[j]][i,14]<-summary(fit0)$tTable["t_w50","p-value"]

B2s3adESTMtab_names[[j]][i,14]<-summary(fit0)$tTable["t_w50","Value"]

#x4

fm0<-as.formula(paste0("y0~t_w50+x1+x2+x3+x4"))

fit0<-try(lme(fixed=fm0,random=~1|cluster,control=lmeControl(opt = "optim")),silent=F)

if(class(fit0)=="try-error"){errorB2_AD<-errorB2_AD+1;i<-i-1;break}

B2s4adALPHAtab_names[[j]][i,14]<-summary(fit0)$tTable["t_w50","p-value"]

B2s4adESTMtab_names[[j]][i,14]<-summary(fit0)$tTable["t_w50","Value"]

#Worst 40%

#x1

fm0<-as.formula(paste0("y0~t_w40+x1"))

fit0<-try(lme(fixed=fm0,random=~1|cluster,control=lmeControl(opt = "optim")),silent=F)

if(class(fit0)=="try-error"){errorB2_AD<-errorB2_AD+1;i<-i-1;break}

B2s1adALPHAtab_names[[j]][i,15]<-summary(fit0)$tTable["t_w40","p-value"]

B2s1adESTMtab_names[[j]][i,15]<-summary(fit0)$tTable["t_w40","Value"]

#x2

fm0<-as.formula(paste0("y0~t_w40+x1+x2"))

fit0<-try(lme(fixed=fm0,random=~1|cluster,control=lmeControl(opt = "optim")),silent=F)

if(class(fit0)=="try-error"){errorB2_AD<-errorB2_AD+1;i<-i-1;break}

B2s2adALPHAtab_names[[j]][i,15]<-summary(fit0)$tTable["t_w40","p-value"]

B2s2adESTMtab_names[[j]][i,15]<-summary(fit0)$tTable["t_w40","Value"]

#x3

fm0<-as.formula(paste0("y0~t_w40+x1+x2+x3"))

fit0<-try(lme(fixed=fm0,random=~1|cluster,control=lmeControl(opt = "optim")),silent=F)

if(class(fit0)=="try-error"){errorB2_AD<-errorB2_AD+1;i<-i-1;break}

B2s3adALPHAtab_names[[j]][i,15]<-summary(fit0)$tTable["t_w40","p-value"]

B2s3adESTMtab_names[[j]][i,15]<-summary(fit0)$tTable["t_w40","Value"]

#x4

fm0<-as.formula(paste0("y0~t_w40+x1+x2+x3+x4"))

fit0<-try(lme(fixed=fm0,random=~1|cluster,control=lmeControl(opt = "optim")),silent=F)

if(class(fit0)=="try-error"){errorB2_AD<-errorB2_AD+1;i<-i-1;break}

B2s4adALPHAtab_names[[j]][i,15]<-summary(fit0)$tTable["t_w40","p-value"]

B2s4adESTMtab_names[[j]][i,15]<-summary(fit0)$tTable["t_w40","Value"]

#Worst 30%

#x1

fm0<-as.formula(paste0("y0~t_w30+x1"))

fit0<-try(lme(fixed=fm0,random=~1|cluster,control=lmeControl(opt = "optim")),silent=F)

if(class(fit0)=="try-error"){errorB2_AD<-errorB2_AD+1;i<-i-1;break}

B2s1adALPHAtab_names[[j]][i,16]<-summary(fit0)$tTable["t_w30","p-value"]

B2s1adESTMtab_names[[j]][i,16]<-summary(fit0)$tTable["t_w30","Value"]

#x2

fm0<-as.formula(paste0("y0~t_w30+x1+x2"))

fit0<-try(lme(fixed=fm0,random=~1|cluster,control=lmeControl(opt = "optim")),silent=F)

if(class(fit0)=="try-error"){errorB2_AD<-errorB2_AD+1;i<-i-1;break}

B2s2adALPHAtab_names[[j]][i,16]<-summary(fit0)$tTable["t_w30","p-value"]

B2s2adESTMtab_names[[j]][i,16]<-summary(fit0)$tTable["t_w30","Value"]

#x3

fm0<-as.formula(paste0("y0~t_w30+x1+x2+x3"))

fit0<-try(lme(fixed=fm0,random=~1|cluster,control=lmeControl(opt = "optim")),silent=F)

if(class(fit0)=="try-error"){errorB2_AD<-errorB2_AD+1;i<-i-1;break}

B2s3adALPHAtab_names[[j]][i,16]<-summary(fit0)$tTable["t_w30","p-value"]

B2s3adESTMtab_names[[j]][i,16]<-summary(fit0)$tTable["t_w30","Value"]

#x4

fm0<-as.formula(paste0("y0~t_w30+x1+x2+x3+x4"))

fit0<-try(lme(fixed=fm0,random=~1|cluster,control=lmeControl(opt = "optim")),silent=F)

if(class(fit0)=="try-error"){errorB2_AD<-errorB2_AD+1;i<-i-1;break}

B2s4adALPHAtab_names[[j]][i,16]<-summary(fit0)$tTable["t_w30","p-value"]

B2s4adESTMtab_names[[j]][i,16]<-summary(fit0)$tTable["t_w30","Value"]

#Worst 20%

#x1

fm0<-as.formula(paste0("y0~t_w20+x1"))

fit0<-try(lme(fixed=fm0,random=~1|cluster,control=lmeControl(opt = "optim")),silent=F)

if(class(fit0)=="try-error"){errorB2_AD<-errorB2_AD+1;i<-i-1;break}

B2s1adALPHAtab_names[[j]][i,17]<-summary(fit0)$tTable["t_w20","p-value"]

B2s1adESTMtab_names[[j]][i,17]<-summary(fit0)$tTable["t_w20","Value"]

#x2

fm0<-as.formula(paste0("y0~t_w20+x1+x2"))

fit0<-try(lme(fixed=fm0,random=~1|cluster,control=lmeControl(opt = "optim")),silent=F)

if(class(fit0)=="try-error"){errorB2_AD<-errorB2_AD+1;i<-i-1;break}

B2s2adALPHAtab_names[[j]][i,17]<-summary(fit0)$tTable["t_w20","p-value"]

B2s2adESTMtab_names[[j]][i,17]<-summary(fit0)$tTable["t_w20","Value"]

#x3

fm0<-as.formula(paste0("y0~t_w20+x1+x2+x3"))

fit0<-try(lme(fixed=fm0,random=~1|cluster,control=lmeControl(opt = "optim")),silent=F)

if(class(fit0)=="try-error"){errorB2_AD<-errorB2_AD+1;i<-i-1;break}

B2s3adALPHAtab_names[[j]][i,17]<-summary(fit0)$tTable["t_w20","p-value"]

B2s3adESTMtab_names[[j]][i,17]<-summary(fit0)$tTable["t_w20","Value"]

#x4

fm0<-as.formula(paste0("y0~t_w20+x1+x2+x3+x4"))

fit0<-try(lme(fixed=fm0,random=~1|cluster,control=lmeControl(opt = "optim")),silent=F)

if(class(fit0)=="try-error"){errorB2_AD<-errorB2_AD+1;i<-i-1;break}

B2s4adALPHAtab_names[[j]][i,17]<-summary(fit0)$tTable["t_w20","p-value"]

B2s4adESTMtab_names[[j]][i,17]<-summary(fit0)$tTable["t_w20","Value"]

#Worst 10%

#x1

fm0<-as.formula(paste0("y0~t_w10+x1"))

fit0<-try(lme(fixed=fm0,random=~1|cluster,control=lmeControl(opt = "optim")),silent=F)

if(class(fit0)=="try-error"){errorB2_AD<-errorB2_AD+1;i<-i-1;break}

B2s1adALPHAtab_names[[j]][i,18]<-summary(fit0)$tTable["t_w10","p-value"]

B2s1adESTMtab_names[[j]][i,18]<-summary(fit0)$tTable["t_w10","Value"]

#x2

fm0<-as.formula(paste0("y0~t_w10+x1+x2"))

fit0<-try(lme(fixed=fm0,random=~1|cluster,control=lmeControl(opt = "optim")),silent=F)

if(class(fit0)=="try-error"){errorB2_AD<-errorB2_AD+1;i<-i-1;break}

B2s2adALPHAtab_names[[j]][i,18]<-summary(fit0)$tTable["t_w10","p-value"]

B2s2adESTMtab_names[[j]][i,18]<-summary(fit0)$tTable["t_w10","Value"]

#x3

fm0<-as.formula(paste0("y0~t_w10+x1+x2+x3"))

fit0<-try(lme(fixed=fm0,random=~1|cluster,control=lmeControl(opt = "optim")),silent=F)

if(class(fit0)=="try-error"){errorB2_AD<-errorB2_AD+1;i<-i-1;break}

B2s3adALPHAtab_names[[j]][i,18]<-summary(fit0)$tTable["t_w10","p-value"]

B2s3adESTMtab_names[[j]][i,18]<-summary(fit0)$tTable["t_w10","Value"]

#x4

fm0<-as.formula(paste0("y0~t_w10+x1+x2+x3+x4"))

fit0<-try(lme(fixed=fm0,random=~1|cluster,control=lmeControl(opt = "optim")),silent=F)

if(class(fit0)=="try-error"){errorB2_AD<-errorB2_AD+1;i<-i-1;break}

B2s4adALPHAtab_names[[j]][i,18]<-summary(fit0)$tTable["t_w10","p-value"]

B2s4adESTMtab_names[[j]][i,18]<-summary(fit0)$tTable["t_w10","Value"]

################################

#generate the average y for those in the trt and control from each candidate set

mu.pwr_b10<-mu.y+theta*t_b10

mu.pwr_b20<-mu.y+theta*t_b20

mu.pwr_b30<-mu.y+theta*t_b30

mu.pwr_b40<-mu.y+theta*t_b40

mu.pwr_b50<-mu.y+theta*t_b50

mu.pwr_b60<-mu.y+theta*t_b60

mu.pwr_b70<-mu.y+theta*t_b70

mu.pwr_b80<-mu.y+theta*t_b80

mu.pwr_b90<-mu.y+theta*t_b90

mu.pwr_w10<-mu.y+theta*t_w10

mu.pwr_w20<-mu.y+theta*t_w20

mu.pwr_w30<-mu.y+theta*t_w30

mu.pwr_w40<-mu.y+theta*t_w40

mu.pwr_w50<-mu.y+theta*t_w50

mu.pwr_w60<-mu.y+theta*t_w60

mu.pwr_w70<-mu.y+theta*t_w70

mu.pwr_w80<-mu.y+theta*t_w80

mu.pwr_w90<-mu.y+theta*t_w90

#y generated from normal with mean dependent on trt group

y1_b10<-rnorm(obs,mu.pwr_b10,sig_e)

y1_b20<-rnorm(obs,mu.pwr_b20,sig_e)

y1_b30<-rnorm(obs,mu.pwr_b30,sig_e)

y1_b40<-rnorm(obs,mu.pwr_b40,sig_e)

y1_b50<-rnorm(obs,mu.pwr_b50,sig_e)

y1_b60<-rnorm(obs,mu.pwr_b60,sig_e)

y1_b70<-rnorm(obs,mu.pwr_b70,sig_e)

y1_b80<-rnorm(obs,mu.pwr_b80,sig_e)

y1_b90<-rnorm(obs,mu.pwr_b90,sig_e)

y1_w10<-rnorm(obs,mu.pwr_w10,sig_e)

y1_w20<-rnorm(obs,mu.pwr_w20,sig_e)

y1_w30<-rnorm(obs,mu.pwr_w30,sig_e)

y1_w40<-rnorm(obs,mu.pwr_w40,sig_e)

y1_w50<-rnorm(obs,mu.pwr_w50,sig_e)

y1_w60<-rnorm(obs,mu.pwr_w60,sig_e)

y1_w70<-rnorm(obs,mu.pwr_w70,sig_e)

y1_w80<-rnorm(obs,mu.pwr_w80,sig_e)

y1_w90<-rnorm(obs,mu.pwr_w90,sig_e)

## Power ##

# Adjusted analysis only: #

#Best 10%

#x1

fm1<-as.formula(paste0("y1_b10~t_b10+x1"))

fit1<-try(lme(fixed=fm1,random=~1|cluster,control=lmeControl(opt = "optim")),silent=F)

if(class(fit1)=="try-error"){errorB2_AD<-errorB2_AD+1;i<-i-1;break}

B2s1adPOWERtab_names[[j]][i,1]<-summary(fit1)$tTable["t_b10","p-value"]

B2s1adBIAStab_names[[j]][i,1]<-summary(fit1)$tTable["t_b10","Value"]-theta

#x2

fm1<-as.formula(paste0("y1_b10~t_b10+x1+x2"))

fit1<-try(lme(fixed=fm1,random=~1|cluster,control=lmeControl(opt = "optim")),silent=F)

if(class(fit1)=="try-error"){errorB2_AD<-errorB2_AD+1;i<-i-1;break}

B2s2adPOWERtab_names[[j]][i,1]<-summary(fit1)$tTable["t_b10","p-value"]

B2s2adBIAStab_names[[j]][i,1]<-summary(fit1)$tTable["t_b10","Value"]-theta

#x3

fm1<-as.formula(paste0("y1_b10~t_b10+x1+x2+x3"))

fit1<-try(lme(fixed=fm1,random=~1|cluster,control=lmeControl(opt = "optim")),silent=F)

if(class(fit1)=="try-error"){errorB2_AD<-errorB2_AD+1;i<-i-1;break}

B2s3adPOWERtab_names[[j]][i,1]<-summary(fit1)$tTable["t_b10","p-value"]

B2s3adBIAStab_names[[j]][i,1]<-summary(fit1)$tTable["t_b10","Value"]-theta

#x4

fm1<-as.formula(paste0("y1_b10~t_b10+x1+x2+x3+x4"))

fit1<-try(lme(fixed=fm1,random=~1|cluster,control=lmeControl(opt = "optim")),silent=F)

if(class(fit1)=="try-error"){errorB2_AD<-errorB2_AD+1;i<-i-1;break}

B2s4adPOWERtab_names[[j]][i,1]<-summary(fit1)$tTable["t_b10","p-value"]

B2s4adBIAStab_names[[j]][i,1]<-summary(fit1)$tTable["t_b10","Value"]-theta

#Best 20%

#x1

fm1<-as.formula(paste0("y1_b20~t_b20+x1"))

fit1<-try(lme(fixed=fm1,random=~1|cluster,control=lmeControl(opt = "optim")),silent=F)

if(class(fit1)=="try-error"){errorB2_AD<-errorB2_AD+1;i<-i-1;break}

B2s1adPOWERtab_names[[j]][i,2]<-summary(fit1)$tTable["t_b20","p-value"]

B2s1adBIAStab_names[[j]][i,2]<-summary(fit1)$tTable["t_b20","Value"]-theta

#x2

fm1<-as.formula(paste0("y1_b20~t_b20+x1+x2"))

fit1<-try(lme(fixed=fm1,random=~1|cluster,control=lmeControl(opt = "optim")),silent=F)

if(class(fit1)=="try-error"){errorB2_AD<-errorB2_AD+1;i<-i-1;break}

B2s2adPOWERtab_names[[j]][i,2]<-summary(fit1)$tTable["t_b20","p-value"]

B2s2adBIAStab_names[[j]][i,2]<-summary(fit1)$tTable["t_b20","Value"]-theta

#x3

fm1<-as.formula(paste0("y1_b20~t_b20+x1+x2+x3"))

fit1<-try(lme(fixed=fm1,random=~1|cluster,control=lmeControl(opt = "optim")),silent=F)

if(class(fit1)=="try-error"){errorB2_AD<-errorB2_AD+1;i<-i-1;break}

B2s3adPOWERtab_names[[j]][i,2]<-summary(fit1)$tTable["t_b20","p-value"]

B2s3adBIAStab_names[[j]][i,2]<-summary(fit1)$tTable["t_b20","Value"]-theta

#x4

fm1<-as.formula(paste0("y1_b20~t_b20+x1+x2+x3+x4"))

fit1<-try(lme(fixed=fm1,random=~1|cluster,control=lmeControl(opt = "optim")),silent=F)

if(class(fit1)=="try-error"){errorB2_AD<-errorB2_AD+1;i<-i-1;break}

B2s4adPOWERtab_names[[j]][i,2]<-summary(fit1)$tTable["t_b20","p-value"]

B2s4adBIAStab_names[[j]][i,2]<-summary(fit1)$tTable["t_b20","Value"]-theta

#Best 30%

#x1

fm1<-as.formula(paste0("y1_b30~t_b30+x1"))

fit1<-try(lme(fixed=fm1,random=~1|cluster,control=lmeControl(opt = "optim")),silent=F)

if(class(fit1)=="try-error"){errorB2_AD<-errorB2_AD+1;i<-i-1;break}

B2s1adPOWERtab_names[[j]][i,3]<-summary(fit1)$tTable["t_b30","p-value"]

B2s1adBIAStab_names[[j]][i,3]<-summary(fit1)$tTable["t_b30","Value"]-theta

#x2

fm1<-as.formula(paste0("y1_b30~t_b30+x1+x2"))

fit1<-try(lme(fixed=fm1,random=~1|cluster,control=lmeControl(opt = "optim")),silent=F)

if(class(fit1)=="try-error"){errorB2_AD<-errorB2_AD+1;i<-i-1;break}

B2s2adPOWERtab_names[[j]][i,3]<-summary(fit1)$tTable["t_b30","p-value"]

B2s2adBIAStab_names[[j]][i,3]<-summary(fit1)$tTable["t_b30","Value"]-theta

#x3

fm1<-as.formula(paste0("y1_b30~t_b30+x1+x2+x3"))

fit1<-try(lme(fixed=fm1,random=~1|cluster,control=lmeControl(opt = "optim")),silent=F)

if(class(fit1)=="try-error"){errorB2_AD<-errorB2_AD+1;i<-i-1;break}

B2s3adPOWERtab_names[[j]][i,3]<-summary(fit1)$tTable["t_b30","p-value"]

B2s3adBIAStab_names[[j]][i,3]<-summary(fit1)$tTable["t_b30","Value"]-theta

#x4

fm1<-as.formula(paste0("y1_b30~t_b30+x1+x2+x3+x4"))

fit1<-try(lme(fixed=fm1,random=~1|cluster,control=lmeControl(opt = "optim")),silent=F)

if(class(fit1)=="try-error"){errorB2_AD<-errorB2_AD+1;i<-i-1;break}

B2s4adPOWERtab_names[[j]][i,3]<-summary(fit1)$tTable["t_b30","p-value"]

B2s4adBIAStab_names[[j]][i,3]<-summary(fit1)$tTable["t_b30","Value"]-theta

#Best 40%

#x1

fm1<-as.formula(paste0("y1_b40~t_b40+x1"))

fit1<-try(lme(fixed=fm1,random=~1|cluster,control=lmeControl(opt = "optim")),silent=F)

if(class(fit1)=="try-error"){errorB2_AD<-errorB2_AD+1;i<-i-1;break}

B2s1adPOWERtab_names[[j]][i,4]<-summary(fit1)$tTable["t_b40","p-value"]

B2s1adBIAStab_names[[j]][i,4]<-summary(fit1)$tTable["t_b40","Value"]-theta

#x2

fm1<-as.formula(paste0("y1_b40~t_b40+x1+x2"))

fit1<-try(lme(fixed=fm1,random=~1|cluster,control=lmeControl(opt = "optim")),silent=F)

if(class(fit1)=="try-error"){errorB2_AD<-errorB2_AD+1;i<-i-1;break}

B2s2adPOWERtab_names[[j]][i,4]<-summary(fit1)$tTable["t_b40","p-value"]

B2s2adBIAStab_names[[j]][i,4]<-summary(fit1)$tTable["t_b40","Value"]-theta

#x3

fm1<-as.formula(paste0("y1_b40~t_b40+x1+x2+x3"))

fit1<-try(lme(fixed=fm1,random=~1|cluster,control=lmeControl(opt = "optim")),silent=F)

if(class(fit1)=="try-error"){errorB2_AD<-errorB2_AD+1;i<-i-1;break}

B2s3adPOWERtab_names[[j]][i,4]<-summary(fit1)$tTable["t_b40","p-value"]

B2s3adBIAStab_names[[j]][i,4]<-summary(fit1)$tTable["t_b40","Value"]-theta

#x4

fm1<-as.formula(paste0("y1_b40~t_b40+x1+x2+x3+x4"))

fit1<-try(lme(fixed=fm1,random=~1|cluster,control=lmeControl(opt = "optim")),silent=F)

if(class(fit1)=="try-error"){errorB2_AD<-errorB2_AD+1;i<-i-1;break}

B2s4adPOWERtab_names[[j]][i,4]<-summary(fit1)$tTable["t_b40","p-value"]

B2s4adBIAStab_names[[j]][i,4]<-summary(fit1)$tTable["t_b40","Value"]-theta

#Best 50%

#x1

fm1<-as.formula(paste0("y1_b50~t_b50+x1"))

fit1<-try(lme(fixed=fm1,random=~1|cluster,control=lmeControl(opt = "optim")),silent=F)

if(class(fit1)=="try-error"){errorB2_AD<-errorB2_AD+1;i<-i-1;break}

B2s1adPOWERtab_names[[j]][i,5]<-summary(fit1)$tTable["t_b50","p-value"]

B2s1adBIAStab_names[[j]][i,5]<-summary(fit1)$tTable["t_b50","Value"]-theta

#x2

fm1<-as.formula(paste0("y1_b50~t_b50+x1+x2"))

fit1<-try(lme(fixed=fm1,random=~1|cluster,control=lmeControl(opt = "optim")),silent=F)

if(class(fit1)=="try-error"){errorB2_AD<-errorB2_AD+1;i<-i-1;break}

B2s2adPOWERtab_names[[j]][i,5]<-summary(fit1)$tTable["t_b50","p-value"]

B2s2adBIAStab_names[[j]][i,5]<-summary(fit1)$tTable["t_b50","Value"]-theta

#x3

fm1<-as.formula(paste0("y1_b50~t_b50+x1+x2+x3"))

fit1<-try(lme(fixed=fm1,random=~1|cluster,control=lmeControl(opt = "optim")),silent=F)

if(class(fit1)=="try-error"){errorB2_AD<-errorB2_AD+1;i<-i-1;break}

B2s3adPOWERtab_names[[j]][i,5]<-summary(fit1)$tTable["t_b50","p-value"]

B2s3adBIAStab_names[[j]][i,5]<-summary(fit1)$tTable["t_b50","Value"]-theta

#x4

fm1<-as.formula(paste0("y1_b50~t_b50+x1+x2+x3+x4"))

fit1<-try(lme(fixed=fm1,random=~1|cluster,control=lmeControl(opt = "optim")),silent=F)

if(class(fit1)=="try-error"){errorB2_AD<-errorB2_AD+1;i<-i-1;break}

B2s4adPOWERtab_names[[j]][i,5]<-summary(fit1)$tTable["t_b50","p-value"]

B2s4adBIAStab_names[[j]][i,5]<-summary(fit1)$tTable["t_b50","Value"]-theta

#Best 60%

#x1

fm1<-as.formula(paste0("y1_b60~t_b60+x1"))

fit1<-try(lme(fixed=fm1,random=~1|cluster,control=lmeControl(opt = "optim")),silent=F)

if(class(fit1)=="try-error"){errorB2_AD<-errorB2_AD+1;i<-i-1;break}

B2s1adPOWERtab_names[[j]][i,6]<-summary(fit1)$tTable["t_b60","p-value"]

B2s1adBIAStab_names[[j]][i,6]<-summary(fit1)$tTable["t_b60","Value"]-theta

#x2

fm1<-as.formula(paste0("y1_b60~t_b60+x1+x2"))

fit1<-try(lme(fixed=fm1,random=~1|cluster,control=lmeControl(opt = "optim")),silent=F)

if(class(fit1)=="try-error"){errorB2_AD<-errorB2_AD+1;i<-i-1;break}

B2s2adPOWERtab_names[[j]][i,6]<-summary(fit1)$tTable["t_b60","p-value"]

B2s2adBIAStab_names[[j]][i,6]<-summary(fit1)$tTable["t_b60","Value"]-theta

#x3

fm1<-as.formula(paste0("y1_b60~t_b60+x1+x2+x3"))

fit1<-try(lme(fixed=fm1,random=~1|cluster,control=lmeControl(opt = "optim")),silent=F)

if(class(fit1)=="try-error"){errorB2_AD<-errorB2_AD+1;i<-i-1;break}

B2s3adPOWERtab_names[[j]][i,6]<-summary(fit1)$tTable["t_b60","p-value"]

B2s3adBIAStab_names[[j]][i,6]<-summary(fit1)$tTable["t_b60","Value"]-theta

#x4

fm1<-as.formula(paste0("y1_b60~t_b60+x1+x2+x3+x4"))

fit1<-try(lme(fixed=fm1,random=~1|cluster,control=lmeControl(opt = "optim")),silent=F)

if(class(fit1)=="try-error"){errorB2_AD<-errorB2_AD+1;i<-i-1;break}

B2s4adPOWERtab_names[[j]][i,6]<-summary(fit1)$tTable["t_b60","p-value"]

B2s4adBIAStab_names[[j]][i,6]<-summary(fit1)$tTable["t_b60","Value"]-theta

#Best 70%

#x1

fm1<-as.formula(paste0("y1_b70~t_b70+x1"))

fit1<-try(lme(fixed=fm1,random=~1|cluster,control=lmeControl(opt = "optim")),silent=F)

if(class(fit1)=="try-error"){errorB2_AD<-errorB2_AD+1;i<-i-1;break}

B2s1adPOWERtab_names[[j]][i,7]<-summary(fit1)$tTable["t_b70","p-value"]

B2s1adBIAStab_names[[j]][i,7]<-summary(fit1)$tTable["t_b70","Value"]-theta

#x2

fm1<-as.formula(paste0("y1_b70~t_b70+x1+x2"))

fit1<-try(lme(fixed=fm1,random=~1|cluster,control=lmeControl(opt = "optim")),silent=F)

if(class(fit1)=="try-error"){errorB2_AD<-errorB2_AD+1;i<-i-1;break}

B2s2adPOWERtab_names[[j]][i,7]<-summary(fit1)$tTable["t_b70","p-value"]

B2s2adBIAStab_names[[j]][i,7]<-summary(fit1)$tTable["t_b70","Value"]-theta

#x3

fm1<-as.formula(paste0("y1_b70~t_b70+x1+x2+x3"))

fit1<-try(lme(fixed=fm1,random=~1|cluster,control=lmeControl(opt = "optim")),silent=F)

if(class(fit1)=="try-error"){errorB2_AD<-errorB2_AD+1;i<-i-1;break}

B2s3adPOWERtab_names[[j]][i,7]<-summary(fit1)$tTable["t_b70","p-value"]

B2s3adBIAStab_names[[j]][i,7]<-summary(fit1)$tTable["t_b70","Value"]-theta

#x4

fm1<-as.formula(paste0("y1_b70~t_b70+x1+x2+x3+x4"))

fit1<-try(lme(fixed=fm1,random=~1|cluster,control=lmeControl(opt = "optim")),silent=F)

if(class(fit1)=="try-error"){errorB2_AD<-errorB2_AD+1;i<-i-1;break}

B2s4adPOWERtab_names[[j]][i,7]<-summary(fit1)$tTable["t_b70","p-value"]

B2s4adBIAStab_names[[j]][i,7]<-summary(fit1)$tTable["t_b70","Value"]-theta

#Best 80%

#x1

fm1<-as.formula(paste0("y1_b80~t_b80+x1"))

fit1<-try(lme(fixed=fm1,random=~1|cluster,control=lmeControl(opt = "optim")),silent=F)

if(class(fit1)=="try-error"){errorB2_AD<-errorB2_AD+1;i<-i-1;break}

B2s1adPOWERtab_names[[j]][i,8]<-summary(fit1)$tTable["t_b80","p-value"]

B2s1adBIAStab_names[[j]][i,8]<-summary(fit1)$tTable["t_b80","Value"]-theta

#x2

fm1<-as.formula(paste0("y1_b80~t_b80+x1+x2"))

fit1<-try(lme(fixed=fm1,random=~1|cluster,control=lmeControl(opt = "optim")),silent=F)

if(class(fit1)=="try-error"){errorB2_AD<-errorB2_AD+1;i<-i-1;break}

B2s2adPOWERtab_names[[j]][i,8]<-summary(fit1)$tTable["t_b80","p-value"]

B2s2adBIAStab_names[[j]][i,8]<-summary(fit1)$tTable["t_b80","Value"]-theta

#x3

fm1<-as.formula(paste0("y1_b80~t_b80+x1+x2+x3"))

fit1<-try(lme(fixed=fm1,random=~1|cluster,control=lmeControl(opt = "optim")),silent=F)

if(class(fit1)=="try-error"){errorB2_AD<-errorB2_AD+1;i<-i-1;break}

B2s3adPOWERtab_names[[j]][i,8]<-summary(fit1)$tTable["t_b80","p-value"]

B2s3adBIAStab_names[[j]][i,8]<-summary(fit1)$tTable["t_b80","Value"]-theta

#x4

fm1<-as.formula(paste0("y1_b80~t_b80+x1+x2+x3+x4"))

fit1<-try(lme(fixed=fm1,random=~1|cluster,control=lmeControl(opt = "optim")),silent=F)

if(class(fit1)=="try-error"){errorB2_AD<-errorB2_AD+1;i<-i-1;break}

B2s4adPOWERtab_names[[j]][i,8]<-summary(fit1)$tTable["t_b80","p-value"]

B2s4adBIAStab_names[[j]][i,8]<-summary(fit1)$tTable["t_b80","Value"]-theta

#Best 90%

#x1

fm1<-as.formula(paste0("y1_b90~t_b90+x1"))

fit1<-try(lme(fixed=fm1,random=~1|cluster,control=lmeControl(opt = "optim")),silent=F)

if(class(fit1)=="try-error"){errorB2_AD<-errorB2_AD+1;i<-i-1;break}

B2s1adPOWERtab_names[[j]][i,9]<-summary(fit1)$tTable["t_b90","p-value"]

B2s1adBIAStab_names[[j]][i,9]<-summary(fit1)$tTable["t_b90","Value"]-theta

#x2

fm1<-as.formula(paste0("y1_b90~t_b90+x1+x2"))

fit1<-try(lme(fixed=fm1,random=~1|cluster,control=lmeControl(opt = "optim")),silent=F)

if(class(fit1)=="try-error"){errorB2_AD<-errorB2_AD+1;i<-i-1;break}

B2s2adPOWERtab_names[[j]][i,9]<-summary(fit1)$tTable["t_b90","p-value"]

B2s2adBIAStab_names[[j]][i,9]<-summary(fit1)$tTable["t_b90","Value"]-theta

#x3

fm1<-as.formula(paste0("y1_b90~t_b90+x1+x2+x3"))

fit1<-try(lme(fixed=fm1,random=~1|cluster,control=lmeControl(opt = "optim")),silent=F)

if(class(fit1)=="try-error"){errorB2_AD<-errorB2_AD+1;i<-i-1;break}

B2s3adPOWERtab_names[[j]][i,9]<-summary(fit1)$tTable["t_b90","p-value"]

B2s3adBIAStab_names[[j]][i,9]<-summary(fit1)$tTable["t_b90","Value"]-theta

#x4

fm1<-as.formula(paste0("y1_b90~t_b90+x1+x2+x3+x4"))

fit1<-try(lme(fixed=fm1,random=~1|cluster,control=lmeControl(opt = "optim")),silent=F)

if(class(fit1)=="try-error"){errorB2_AD<-errorB2_AD+1;i<-i-1;break}

B2s4adPOWERtab_names[[j]][i,9]<-summary(fit1)$tTable["t_b90","p-value"]

B2s4adBIAStab_names[[j]][i,9]<-summary(fit1)$tTable["t_b90","Value"]-theta

#Worst 90%

#x1

fm1<-as.formula(paste0("y1_w90~t_w90+x1"))

fit1<-try(lme(fixed=fm1,random=~1|cluster,control=lmeControl(opt = "optim")),silent=F)

if(class(fit1)=="try-error"){errorB2_AD<-errorB2_AD+1;i<-i-1;break}

B2s1adPOWERtab_names[[j]][i,10]<-summary(fit1)$tTable["t_w90","p-value"]

B2s1adBIAStab_names[[j]][i,10]<-summary(fit1)$tTable["t_w90","Value"]-theta

#x2

fm1<-as.formula(paste0("y1_w90~t_w90+x1+x2"))

fit1<-try(lme(fixed=fm1,random=~1|cluster,control=lmeControl(opt = "optim")),silent=F)

if(class(fit1)=="try-error"){errorB2_AD<-errorB2_AD+1;i<-i-1;break}

B2s2adPOWERtab_names[[j]][i,10]<-summary(fit1)$tTable["t_w90","p-value"]

B2s2adBIAStab_names[[j]][i,10]<-summary(fit1)$tTable["t_w90","Value"]-theta

#x3

fm1<-as.formula(paste0("y1_w90~t_w90+x1+x2+x3"))

fit1<-try(lme(fixed=fm1,random=~1|cluster,control=lmeControl(opt = "optim")),silent=F)

if(class(fit1)=="try-error"){errorB2_AD<-errorB2_AD+1;i<-i-1;break}

B2s3adPOWERtab_names[[j]][i,10]<-summary(fit1)$tTable["t_w90","p-value"]

B2s3adBIAStab_names[[j]][i,10]<-summary(fit1)$tTable["t_w90","Value"]-theta

#x4

fm1<-as.formula(paste0("y1_w90~t_w90+x1+x2+x3+x4"))

fit1<-try(lme(fixed=fm1,random=~1|cluster,control=lmeControl(opt = "optim")),silent=F)

if(class(fit1)=="try-error"){errorB2_AD<-errorB2_AD+1;i<-i-1;break}

B2s4adPOWERtab_names[[j]][i,10]<-summary(fit1)$tTable["t_w90","p-value"]

B2s4adBIAStab_names[[j]][i,10]<-summary(fit1)$tTable["t_w90","Value"]-theta

#Worst 80%

#x1

fm1<-as.formula(paste0("y1_w80~t_w80+x1"))

fit1<-try(lme(fixed=fm1,random=~1|cluster,control=lmeControl(opt = "optim")),silent=F)

if(class(fit1)=="try-error"){errorB2_AD<-errorB2_AD+1;i<-i-1;break}

B2s1adPOWERtab_names[[j]][i,11]<-summary(fit1)$tTable["t_w80","p-value"]

B2s1adBIAStab_names[[j]][i,11]<-summary(fit1)$tTable["t_w80","Value"]-theta

#x2

fm1<-as.formula(paste0("y1_w80~t_w80+x1+x2"))

fit1<-try(lme(fixed=fm1,random=~1|cluster,control=lmeControl(opt = "optim")),silent=F)

if(class(fit1)=="try-error"){errorB2_AD<-errorB2_AD+1;i<-i-1;break}

B2s2adPOWERtab_names[[j]][i,11]<-summary(fit1)$tTable["t_w80","p-value"]

B2s2adBIAStab_names[[j]][i,11]<-summary(fit1)$tTable["t_w80","Value"]-theta

#x3

fm1<-as.formula(paste0("y1_w80~t_w80+x1+x2+x3"))

fit1<-try(lme(fixed=fm1,random=~1|cluster,control=lmeControl(opt = "optim")),silent=F)

if(class(fit1)=="try-error"){errorB2_AD<-errorB2_AD+1;i<-i-1;break}

B2s3adPOWERtab_names[[j]][i,11]<-summary(fit1)$tTable["t_w80","p-value"]

B2s3adBIAStab_names[[j]][i,11]<-summary(fit1)$tTable["t_w80","Value"]-theta

#x4

fm1<-as.formula(paste0("y1_w80~t_w80+x1+x2+x3+x4"))

fit1<-try(lme(fixed=fm1,random=~1|cluster,control=lmeControl(opt = "optim")),silent=F)

if(class(fit1)=="try-error"){errorB2_AD<-errorB2_AD+1;i<-i-1;break}

B2s4adPOWERtab_names[[j]][i,11]<-summary(fit1)$tTable["t_w80","p-value"]

B2s4adBIAStab_names[[j]][i,11]<-summary(fit1)$tTable["t_w80","Value"]-theta

#Worst 70%

#x1

fm1<-as.formula(paste0("y1_w70~t_w70+x1"))

fit1<-try(lme(fixed=fm1,random=~1|cluster,control=lmeControl(opt = "optim")),silent=F)

if(class(fit1)=="try-error"){errorB2_AD<-errorB2_AD+1;i<-i-1;break}

B2s1adPOWERtab_names[[j]][i,12]<-summary(fit1)$tTable["t_w70","p-value"]

B2s1adBIAStab_names[[j]][i,12]<-summary(fit1)$tTable["t_w70","Value"]-theta

#x2

fm1<-as.formula(paste0("y1_w70~t_w70+x1+x2"))

fit1<-try(lme(fixed=fm1,random=~1|cluster,control=lmeControl(opt = "optim")),silent=F)

if(class(fit1)=="try-error"){errorB2_AD<-errorB2_AD+1;i<-i-1;break}

B2s2adPOWERtab_names[[j]][i,12]<-summary(fit1)$tTable["t_w70","p-value"]

B2s2adBIAStab_names[[j]][i,12]<-summary(fit1)$tTable["t_w70","Value"]-theta

#x3

fm1<-as.formula(paste0("y1_w70~t_w70+x1+x2+x3"))

fit1<-try(lme(fixed=fm1,random=~1|cluster,control=lmeControl(opt = "optim")),silent=F)

if(class(fit1)=="try-error"){errorB2_AD<-errorB2_AD+1;i<-i-1;break}

B2s3adPOWERtab_names[[j]][i,12]<-summary(fit1)$tTable["t_w70","p-value"]

B2s3adBIAStab_names[[j]][i,12]<-summary(fit1)$tTable["t_w70","Value"]-theta

#x4

fm1<-as.formula(paste0("y1_w70~t_w70+x1+x2+x3+x4"))

fit1<-try(lme(fixed=fm1,random=~1|cluster,control=lmeControl(opt = "optim")),silent=F)

if(class(fit1)=="try-error"){errorB2_AD<-errorB2_AD+1;i<-i-1;break}

B2s4adPOWERtab_names[[j]][i,12]<-summary(fit1)$tTable["t_w70","p-value"]

B2s4adBIAStab_names[[j]][i,12]<-summary(fit1)$tTable["t_w70","Value"]-theta

#Worst 60%

#x1

fm1<-as.formula(paste0("y1_w60~t_w60+x1"))

fit1<-try(lme(fixed=fm1,random=~1|cluster,control=lmeControl(opt = "optim")),silent=F)

if(class(fit1)=="try-error"){errorB2_AD<-errorB2_AD+1;i<-i-1;break}

B2s1adPOWERtab_names[[j]][i,13]<-summary(fit1)$tTable["t_w60","p-value"]

B2s1adBIAStab_names[[j]][i,13]<-summary(fit1)$tTable["t_w60","Value"]-theta

#x2

fm1<-as.formula(paste0("y1_w60~t_w60+x1+x2"))

fit1<-try(lme(fixed=fm1,random=~1|cluster,control=lmeControl(opt = "optim")),silent=F)

if(class(fit1)=="try-error"){errorB2_AD<-errorB2_AD+1;i<-i-1;break}

B2s2adPOWERtab_names[[j]][i,13]<-summary(fit1)$tTable["t_w60","p-value"]

B2s2adBIAStab_names[[j]][i,13]<-summary(fit1)$tTable["t_w60","Value"]-theta

#x3

fm1<-as.formula(paste0("y1_w60~t_w60+x1+x2+x3"))

fit1<-try(lme(fixed=fm1,random=~1|cluster,control=lmeControl(opt = "optim")),silent=F)

if(class(fit1)=="try-error"){errorB2_AD<-errorB2_AD+1;i<-i-1;break}

B2s3adPOWERtab_names[[j]][i,13]<-summary(fit1)$tTable["t_w60","p-value"]

B2s3adBIAStab_names[[j]][i,13]<-summary(fit1)$tTable["t_w60","Value"]-theta

#x4

fm1<-as.formula(paste0("y1_w60~t_w60+x1+x2+x3+x4"))

fit1<-try(lme(fixed=fm1,random=~1|cluster,control=lmeControl(opt = "optim")),silent=F)

if(class(fit1)=="try-error"){errorB2_AD<-errorB2_AD+1;i<-i-1;break}

B2s4adPOWERtab_names[[j]][i,13]<-summary(fit1)$tTable["t_w60","p-value"]

B2s4adBIAStab_names[[j]][i,13]<-summary(fit1)$tTable["t_w60","Value"]-theta

#Worst 50%

#x1

fm1<-as.formula(paste0("y1_w50~t_w50+x1"))

fit1<-try(lme(fixed=fm1,random=~1|cluster,control=lmeControl(opt = "optim")),silent=F)

if(class(fit1)=="try-error"){errorB2_AD<-errorB2_AD+1;i<-i-1;break}

B2s1adPOWERtab_names[[j]][i,14]<-summary(fit1)$tTable["t_w50","p-value"]

B2s1adBIAStab_names[[j]][i,14]<-summary(fit1)$tTable["t_w50","Value"]-theta

#x2

fm1<-as.formula(paste0("y1_w50~t_w50+x1+x2"))

fit1<-try(lme(fixed=fm1,random=~1|cluster,control=lmeControl(opt = "optim")),silent=F)

if(class(fit1)=="try-error"){errorB2_AD<-errorB2_AD+1;i<-i-1;break}

B2s2adPOWERtab_names[[j]][i,14]<-summary(fit1)$tTable["t_w50","p-value"]

B2s2adBIAStab_names[[j]][i,14]<-summary(fit1)$tTable["t_w50","Value"]-theta

#x3

fm1<-as.formula(paste0("y1_w50~t_w50+x1+x2+x3"))

fit1<-try(lme(fixed=fm1,random=~1|cluster,control=lmeControl(opt = "optim")),silent=F)

if(class(fit1)=="try-error"){errorB2_AD<-errorB2_AD+1;i<-i-1;break}

B2s3adPOWERtab_names[[j]][i,14]<-summary(fit1)$tTable["t_w50","p-value"]

B2s3adBIAStab_names[[j]][i,14]<-summary(fit1)$tTable["t_w50","Value"]-theta

#x4

fm1<-as.formula(paste0("y1_w50~t_w50+x1+x2+x3+x4"))

fit1<-try(lme(fixed=fm1,random=~1|cluster,control=lmeControl(opt = "optim")),silent=F)

if(class(fit1)=="try-error"){errorB2_AD<-errorB2_AD+1;i<-i-1;break}

B2s4adPOWERtab_names[[j]][i,14]<-summary(fit1)$tTable["t_w50","p-value"]

B2s4adBIAStab_names[[j]][i,14]<-summary(fit1)$tTable["t_w50","Value"]-theta

#Worst 40%

#x1

fm1<-as.formula(paste0("y1_w40~t_w40+x1"))

fit1<-try(lme(fixed=fm1,random=~1|cluster,control=lmeControl(opt = "optim")),silent=F)

if(class(fit1)=="try-error"){errorB2_AD<-errorB2_AD+1;i<-i-1;break}

B2s1adPOWERtab_names[[j]][i,15]<-summary(fit1)$tTable["t_w40","p-value"]

B2s1adBIAStab_names[[j]][i,15]<-summary(fit1)$tTable["t_w40","Value"]-theta

#x2

fm1<-as.formula(paste0("y1_w40~t_w40+x1+x2"))

fit1<-try(lme(fixed=fm1,random=~1|cluster,control=lmeControl(opt = "optim")),silent=F)

if(class(fit1)=="try-error"){errorB2_AD<-errorB2_AD+1;i<-i-1;break}

B2s2adPOWERtab_names[[j]][i,15]<-summary(fit1)$tTable["t_w40","p-value"]

B2s2adBIAStab_names[[j]][i,15]<-summary(fit1)$tTable["t_w40","Value"]-theta

#x3

fm1<-as.formula(paste0("y1_w40~t_w40+x1+x2+x3"))

fit1<-try(lme(fixed=fm1,random=~1|cluster,control=lmeControl(opt = "optim")),silent=F)

if(class(fit1)=="try-error"){errorB2_AD<-errorB2_AD+1;i<-i-1;break}

B2s3adPOWERtab_names[[j]][i,15]<-summary(fit1)$tTable["t_w40","p-value"]

B2s3adBIAStab_names[[j]][i,15]<-summary(fit1)$tTable["t_w40","Value"]-theta

#x4

fm1<-as.formula(paste0("y1_w40~t_w40+x1+x2+x3+x4"))

fit1<-try(lme(fixed=fm1,random=~1|cluster,control=lmeControl(opt = "optim")),silent=F)

if(class(fit1)=="try-error"){errorB2_AD<-errorB2_AD+1;i<-i-1;break}

B2s4adPOWERtab_names[[j]][i,15]<-summary(fit1)$tTable["t_w40","p-value"]

B2s4adBIAStab_names[[j]][i,15]<-summary(fit1)$tTable["t_w40","Value"]-theta

#Worst 30%

#x1

fm1<-as.formula(paste0("y1_w30~t_w30+x1"))

fit1<-try(lme(fixed=fm1,random=~1|cluster,control=lmeControl(opt = "optim")),silent=F)

if(class(fit1)=="try-error"){errorB2_AD<-errorB2_AD+1;i<-i-1;break}

B2s1adPOWERtab_names[[j]][i,16]<-summary(fit1)$tTable["t_w30","p-value"]

B2s1adBIAStab_names[[j]][i,16]<-summary(fit1)$tTable["t_w30","Value"]-theta

#x2

fm1<-as.formula(paste0("y1_w30~t_w30+x1+x2"))

fit1<-try(lme(fixed=fm1,random=~1|cluster,control=lmeControl(opt = "optim")),silent=F)

if(class(fit1)=="try-error"){errorB2_AD<-errorB2_AD+1;i<-i-1;break}

B2s2adPOWERtab_names[[j]][i,16]<-summary(fit1)$tTable["t_w30","p-value"]

B2s2adBIAStab_names[[j]][i,16]<-summary(fit1)$tTable["t_w30","Value"]-theta

#x3

fm1<-as.formula(paste0("y1_w30~t_w30+x1+x2+x3"))

fit1<-try(lme(fixed=fm1,random=~1|cluster,control=lmeControl(opt = "optim")),silent=F)

if(class(fit1)=="try-error"){errorB2_AD<-errorB2_AD+1;i<-i-1;break}

B2s3adPOWERtab_names[[j]][i,16]<-summary(fit1)$tTable["t_w30","p-value"]

B2s3adBIAStab_names[[j]][i,16]<-summary(fit1)$tTable["t_w30","Value"]-theta

#x4

fm1<-as.formula(paste0("y1_w30~t_w30+x1+x2+x3+x4"))

fit1<-try(lme(fixed=fm1,random=~1|cluster,control=lmeControl(opt = "optim")),silent=F)

if(class(fit1)=="try-error"){errorB2_AD<-errorB2_AD+1;i<-i-1;break}

B2s4adPOWERtab_names[[j]][i,16]<-summary(fit1)$tTable["t_w30","p-value"]

B2s4adBIAStab_names[[j]][i,16]<-summary(fit1)$tTable["t_w30","Value"]-theta

#Worst 20%

#x1

fm1<-as.formula(paste0("y1_w20~t_w20+x1"))

fit1<-try(lme(fixed=fm1,random=~1|cluster,control=lmeControl(opt = "optim")),silent=F)

if(class(fit1)=="try-error"){errorB2_AD<-errorB2_AD+1;i<-i-1;break}

B2s1adPOWERtab_names[[j]][i,17]<-summary(fit1)$tTable["t_w20","p-value"]

B2s1adBIAStab_names[[j]][i,17]<-summary(fit1)$tTable["t_w20","Value"]-theta

#x2

fm1<-as.formula(paste0("y1_w20~t_w20+x1+x2"))

fit1<-try(lme(fixed=fm1,random=~1|cluster,control=lmeControl(opt = "optim")),silent=F)

if(class(fit1)=="try-error"){errorB2_AD<-errorB2_AD+1;i<-i-1;break}

B2s2adPOWERtab_names[[j]][i,17]<-summary(fit1)$tTable["t_w20","p-value"]

B2s2adBIAStab_names[[j]][i,17]<-summary(fit1)$tTable["t_w20","Value"]-theta

#x3

fm1<-as.formula(paste0("y1_w20~t_w20+x1+x2+x3"))

fit1<-try(lme(fixed=fm1,random=~1|cluster,control=lmeControl(opt = "optim")),silent=F)

if(class(fit1)=="try-error"){errorB2_AD<-errorB2_AD+1;i<-i-1;break}

B2s3adPOWERtab_names[[j]][i,17]<-summary(fit1)$tTable["t_w20","p-value"]

B2s3adBIAStab_names[[j]][i,17]<-summary(fit1)$tTable["t_w20","Value"]-theta

#x4

fm1<-as.formula(paste0("y1_w20~t_w20+x1+x2+x3+x4"))

fit1<-try(lme(fixed=fm1,random=~1|cluster,control=lmeControl(opt = "optim")),silent=F)

if(class(fit1)=="try-error"){errorB2_AD<-errorB2_AD+1;i<-i-1;break}

B2s4adPOWERtab_names[[j]][i,17]<-summary(fit1)$tTable["t_w20","p-value"]

B2s4adBIAStab_names[[j]][i,17]<-summary(fit1)$tTable["t_w20","Value"]-theta

#Worst 10%

#x1

fm1<-as.formula(paste0("y1_w10~t_w10+x1"))

fit1<-try(lme(fixed=fm1,random=~1|cluster,control=lmeControl(opt = "optim")),silent=F)

if(class(fit1)=="try-error"){errorB2_AD<-errorB2_AD+1;i<-i-1;break}

B2s1adPOWERtab_names[[j]][i,18]<-summary(fit1)$tTable["t_w10","p-value"]

B2s1adBIAStab_names[[j]][i,18]<-summary(fit1)$tTable["t_w10","Value"]-theta

#x2

fm1<-as.formula(paste0("y1_w10~t_w10+x1+x2"))

fit1<-try(lme(fixed=fm1,random=~1|cluster,control=lmeControl(opt = "optim")),silent=F)

if(class(fit1)=="try-error"){errorB2_AD<-errorB2_AD+1;i<-i-1;break}

B2s2adPOWERtab_names[[j]][i,18]<-summary(fit1)$tTable["t_w10","p-value"]

B2s2adBIAStab_names[[j]][i,18]<-summary(fit1)$tTable["t_w10","Value"]-theta

#x3

fm1<-as.formula(paste0("y1_w10~t_w10+x1+x2+x3"))

fit1<-try(lme(fixed=fm1,random=~1|cluster,control=lmeControl(opt = "optim")),silent=F)

if(class(fit1)=="try-error"){errorB2_AD<-errorB2_AD+1;i<-i-1;break}

B2s3adPOWERtab_names[[j]][i,18]<-summary(fit1)$tTable["t_w10","p-value"]

B2s3adBIAStab_names[[j]][i,18]<-summary(fit1)$tTable["t_w10","Value"]-theta

#x4

fm1<-as.formula(paste0("y1_w10~t_w10+x1+x2+x3+x4"))

fit1<-try(lme(fixed=fm1,random=~1|cluster,control=lmeControl(opt = "optim")),silent=F)

if(class(fit1)=="try-error"){errorB2_AD<-errorB2_AD+1;i<-i-1;break}

B2s4adPOWERtab_names[[j]][i,18]<-summary(fit1)$tTable["t_w10","p-value"]

B2s4adBIAStab_names[[j]][i,18]<-summary(fit1)$tTable["t_w10","Value"]-theta

}

if(i<tempi){next}

######################

#program control

if(i%%100==0) print(i)

}

##############################################################

#save results

save(SRunALPHAtab,file="SRunALPHAtab.RData")

save(SRunESTMtab,file="SRunESTMtab.RData")

save(SRunPOWERtab,file="SRunPOWERtab.RData")

save(SRunBIAStab,file="SRunBIAStab.RData")

save(SRadALPHAtab,file="SRadALPHAtab.RData")

save(SRadESTMtab,file="SRadESTMtab.RData")

save(SRadPOWERtab,file="SRadPOWERtab.RData")

save(SRadBIAStab,file="SRadBIAStab.RData")

#calculate mean, bias, se and mse for type I error and power

SRunalpha<-apply(SRunALPHAtab<0.05,2,FUN=mean)

SRunaphse<-apply(SRunESTMtab,2,FUN=sd)

SRunaphbias<-apply(SRunESTMtab,2,FUN=mean)

SRunaphmse<-apply(SRunESTMtab^2,2,FUN=mean)

SRunpower<-apply(SRunPOWERtab<0.05,2,FUN=mean)

SRunpwse<-apply(SRunBIAStab,2,FUN=sd)

SRunpwbias<-apply(SRunBIAStab,2,FUN=mean)

SRunpwmse<-apply(SRunBIAStab^2,2,FUN=mean)

SRadalpha<-apply(SRadALPHAtab<0.05,2,FUN=mean)

SRadaphse<-apply(SRadESTMtab,2,FUN=sd)

SRadaphbias<-apply(SRadESTMtab,2,FUN=mean)

SRadaphmse<-apply(SRadESTMtab^2,2,FUN=mean)

SRadpower<-apply(SRadPOWERtab<0.05,2,FUN=mean)

SRadpwse<-apply(SRadBIAStab,2,FUN=sd)

SRadpwbias<-apply(SRadBIAStab,2,FUN=mean)

SRadpwmse<-apply(SRadBIAStab^2,2,FUN=mean)

# save

save(SRunalpha,file="SRunalpha.RData")

save(SRunaphse,file="SRunaphse.RData")

save(SRunaphbias,file="SRunaphbias.RData")

save(SRunaphmse,file="SRunaphmse.RData")

save(SRunpower,file="SRunpower.Rdata")

save(SRunpwse,file="SRunpwse.RData")

save(SRunpwbias,file="SRunpwbias.RData")

save(SRunpwmse,file="SRunpwmse.RData")

save(SRadalpha,file="SRadalpha.RData")

save(SRadaphse,file="SRadaphse.RData")

save(SRadaphbias,file="SRadaphbias.RData")

save(SRadaphmse,file="SRadaphmse.RData")

save(SRadpower,file="SRadpower.Rdata")

save(SRadpwse,file="SRadpwse.RData")

save(SRadpwbias,file="SRadpwbias.RData")

save(SRadpwmse,file="SRadpwmse.RData")

#sink the results to a txt file

sink("Imbalance Score_SRun.txt")

cat("type I error","\n")

apply(SRunALPHAtab<0.05,2,FUN=mean)

cat("s.e.","\n")

apply(SRunESTMtab,2,FUN=sd)

cat("bias","\n")

apply(SRunESTMtab,2,FUN=mean)

cat("MSE","\n")

apply(SRunESTMtab^2,2,FUN=mean)

cat("\n","\n")

cat("power","\n")

apply(SRunPOWERtab<0.05,2,FUN=mean)

cat("s.e.","\n")

apply(SRunBIAStab,2,FUN=sd)

cat("bias","\n")

apply(SRunBIAStab,2,FUN=mean)

cat("MSE","\n")

apply(SRunBIAStab^2,2,FUN=mean)

sink()

sink("Imbalance Score_SRad.txt")

cat("type I error","\n")

apply(SRadALPHAtab<0.05,2,FUN=mean)

cat("s.e.","\n")

apply(SRadESTMtab,2,FUN=sd)

cat("bias","\n")

apply(SRadESTMtab,2,FUN=mean)

cat("MSE","\n")

apply(SRadESTMtab^2,2,FUN=mean)

cat("\n","\n")

cat("power","\n")

apply(SRadPOWERtab<0.05,2,FUN=mean)

cat("s.e.","\n")

apply(SRadBIAStab,2,FUN=sd)

cat("bias","\n")

apply(SRadBIAStab,2,FUN=mean)

cat("MSE","\n")

apply(SRadBIAStab^2,2,FUN=mean)

sink()

###

#save results for B2

save(B2unALPHAtab_b10,file="B2unALPHAtab_b10.RData")

save(B2unESTMtab_b10,file="B2unESTMtab_b10.RData")

save(B2unPOWERtab_b10,file="B2unPOWERtab_b10.RData")

save(B2unBIAStab_b10,file="B2unBIAStab_b10.RData")

#calculate mean, bias, se and mse for type I error and power

B2unalpha_b10<-apply(B2unALPHAtab_b10<0.05,2,FUN=mean)

B2unaphse_b10<-apply(B2unESTMtab_b10,2,FUN=sd)

B2unaphbias_b10<-apply(B2unESTMtab_b10,2,FUN=mean)

B2unaphmse_b10<-apply(B2unESTMtab_b10^2,2,FUN=mean)

B2unpower_b10<-apply(B2unPOWERtab_b10<0.05,2,FUN=mean)

B2unpwse_b10<-apply(B2unBIAStab_b10,2,FUN=sd)

B2unpwbias_b10<-apply(B2unBIAStab_b10,2,FUN=mean)

B2unpwmse_b10<-apply(B2unBIAStab_b10^2,2,FUN=mean)

save(B2unALPHAtab_b20,file="B2unALPHAtab_b20.RData")

save(B2unESTMtab_b20,file="B2unESTMtab_b20.RData")

save(B2unPOWERtab_b20,file="B2unPOWERtab_b20.RData")

save(B2unBIAStab_b20,file="B2unBIAStab_b20.RData")

#calculate mean, bias, se and mse for type I error and power

B2unalpha_b20<-apply(B2unALPHAtab_b20<0.05,2,FUN=mean)

B2unaphse_b20<-apply(B2unESTMtab_b20,2,FUN=sd)

B2unaphbias_b20<-apply(B2unESTMtab_b20,2,FUN=mean)

B2unaphmse_b20<-apply(B2unESTMtab_b20^2,2,FUN=mean)

B2unpower_b20<-apply(B2unPOWERtab_b20<0.05,2,FUN=mean)

B2unpwse_b20<-apply(B2unBIAStab_b20,2,FUN=sd)

B2unpwbias_b20<-apply(B2unBIAStab_b20,2,FUN=mean)

B2unpwmse_b20<-apply(B2unBIAStab_b20^2,2,FUN=mean)

save(B2unALPHAtab_b30,file="B2unALPHAtab_b30.RData")

save(B2unESTMtab_b30,file="B2unESTMtab_b30.RData")

save(B2unPOWERtab_b30,file="B2unPOWERtab_b30.RData")

save(B2unBIAStab_b30,file="B2unBIAStab_b30.RData")

#calculate mean, bias, se and mse for type I error and power

B2unalpha_b30<-apply(B2unALPHAtab_b30<0.05,2,FUN=mean)

B2unaphse_b30<-apply(B2unESTMtab_b30,2,FUN=sd)

B2unaphbias_b30<-apply(B2unESTMtab_b30,2,FUN=mean)

B2unaphmse_b30<-apply(B2unESTMtab_b30^2,2,FUN=mean)

B2unpower_b30<-apply(B2unPOWERtab_b30<0.05,2,FUN=mean)

B2unpwse_b30<-apply(B2unBIAStab_b30,2,FUN=sd)

B2unpwbias_b30<-apply(B2unBIAStab_b30,2,FUN=mean)

B2unpwmse_b30<-apply(B2unBIAStab_b30^2,2,FUN=mean)

save(B2unALPHAtab_b40,file="B2unALPHAtab_b40.RData")

save(B2unESTMtab_b40,file="B2unESTMtab_b40.RData")

save(B2unPOWERtab_b40,file="B2unPOWERtab_b40.RData")

save(B2unBIAStab_b40,file="B2unBIAStab_b40.RData")

#calculate mean, bias, se and mse for type I error and power

B2unalpha_b40<-apply(B2unALPHAtab_b40<0.05,2,FUN=mean)

B2unaphse_b40<-apply(B2unESTMtab_b40,2,FUN=sd)

B2unaphbias_b40<-apply(B2unESTMtab_b40,2,FUN=mean)

B2unaphmse_b40<-apply(B2unESTMtab_b40^2,2,FUN=mean)

B2unpower_b40<-apply(B2unPOWERtab_b40<0.05,2,FUN=mean)

B2unpwse_b40<-apply(B2unBIAStab_b40,2,FUN=sd)

B2unpwbias_b40<-apply(B2unBIAStab_b40,2,FUN=mean)

B2unpwmse_b40<-apply(B2unBIAStab_b40^2,2,FUN=mean)

save(B2unALPHAtab_b50,file="B2unALPHAtab_b50.RData")

save(B2unESTMtab_b50,file="B2unESTMtab_b50.RData")

save(B2unPOWERtab_b50,file="B2unPOWERtab_b50.RData")

save(B2unBIAStab_b50,file="B2unBIAStab_b50.RData")

#calculate mean, bias, se and mse for type I error and power

B2unalpha_b50<-apply(B2unALPHAtab_b50<0.05,2,FUN=mean)

B2unaphse_b50<-apply(B2unESTMtab_b50,2,FUN=sd)

B2unaphbias_b50<-apply(B2unESTMtab_b50,2,FUN=mean)

B2unaphmse_b50<-apply(B2unESTMtab_b50^2,2,FUN=mean)

B2unpower_b50<-apply(B2unPOWERtab_b50<0.05,2,FUN=mean)

B2unpwse_b50<-apply(B2unBIAStab_b50,2,FUN=sd)

B2unpwbias_b50<-apply(B2unBIAStab_b50,2,FUN=mean)

B2unpwmse_b50<-apply(B2unBIAStab_b50^2,2,FUN=mean)

save(B2unALPHAtab_b60,file="B2unALPHAtab_b60.RData")

save(B2unESTMtab_b60,file="B2unESTMtab_b60.RData")

save(B2unPOWERtab_b60,file="B2unPOWERtab_b60.RData")

save(B2unBIAStab_b60,file="B2unBIAStab_b60.RData")

#calculate mean, bias, se and mse for type I error and power

B2unalpha_b60<-apply(B2unALPHAtab_b60<0.05,2,FUN=mean)

B2unaphse_b60<-apply(B2unESTMtab_b60,2,FUN=sd)

B2unaphbias_b60<-apply(B2unESTMtab_b60,2,FUN=mean)

B2unaphmse_b60<-apply(B2unESTMtab_b60^2,2,FUN=mean)

B2unpower_b60<-apply(B2unPOWERtab_b60<0.05,2,FUN=mean)

B2unpwse_b60<-apply(B2unBIAStab_b60,2,FUN=sd)

B2unpwbias_b60<-apply(B2unBIAStab_b60,2,FUN=mean)

B2unpwmse_b60<-apply(B2unBIAStab_b60^2,2,FUN=mean)

save(B2unALPHAtab_b70,file="B2unALPHAtab_b70.RData")

save(B2unESTMtab_b70,file="B2unESTMtab_b70.RData")

save(B2unPOWERtab_b70,file="B2unPOWERtab_b70.RData")

save(B2unBIAStab_b70,file="B2unBIAStab_b70.RData")

#calculate mean, bias, se and mse for type I error and power

B2unalpha_b70<-apply(B2unALPHAtab_b70<0.05,2,FUN=mean)

B2unaphse_b70<-apply(B2unESTMtab_b70,2,FUN=sd)

B2unaphbias_b70<-apply(B2unESTMtab_b70,2,FUN=mean)

B2unaphmse_b70<-apply(B2unESTMtab_b70^2,2,FUN=mean)

B2unpower_b70<-apply(B2unPOWERtab_b70<0.05,2,FUN=mean)

B2unpwse_b70<-apply(B2unBIAStab_b70,2,FUN=sd)

B2unpwbias_b70<-apply(B2unBIAStab_b70,2,FUN=mean)

B2unpwmse_b70<-apply(B2unBIAStab_b70^2,2,FUN=mean)

save(B2unALPHAtab_b80,file="B2unALPHAtab_b80.RData")

save(B2unESTMtab_b80,file="B2unESTMtab_b80.RData")

save(B2unPOWERtab_b80,file="B2unPOWERtab_b80.RData")

save(B2unBIAStab_b80,file="B2unBIAStab_b80.RData")

#calculate mean, bias, se and mse for type I error and power

B2unalpha_b80<-apply(B2unALPHAtab_b80<0.05,2,FUN=mean)

B2unaphse_b80<-apply(B2unESTMtab_b80,2,FUN=sd)

B2unaphbias_b80<-apply(B2unESTMtab_b80,2,FUN=mean)

B2unaphmse_b80<-apply(B2unESTMtab_b80^2,2,FUN=mean)

B2unpower_b80<-apply(B2unPOWERtab_b80<0.05,2,FUN=mean)

B2unpwse_b80<-apply(B2unBIAStab_b80,2,FUN=sd)

B2unpwbias_b80<-apply(B2unBIAStab_b80,2,FUN=mean)

B2unpwmse_b80<-apply(B2unBIAStab_b80^2,2,FUN=mean)

save(B2unALPHAtab_b90,file="B2unALPHAtab_b90.RData")

save(B2unESTMtab_b90,file="B2unESTMtab_b90.RData")

save(B2unPOWERtab_b90,file="B2unPOWERtab_b90.RData")

save(B2unBIAStab_b90,file="B2unBIAStab_b90.RData")

#calculate mean, bias, se and mse for type I error and power

B2unalpha_b90<-apply(B2unALPHAtab_b90<0.05,2,FUN=mean)

B2unaphse_b90<-apply(B2unESTMtab_b90,2,FUN=sd)

B2unaphbias_b90<-apply(B2unESTMtab_b90,2,FUN=mean)

B2unaphmse_b90<-apply(B2unESTMtab_b90^2,2,FUN=mean)

B2unpower_b90<-apply(B2unPOWERtab_b90<0.05,2,FUN=mean)

B2unpwse_b90<-apply(B2unBIAStab_b90,2,FUN=sd)

B2unpwbias_b90<-apply(B2unBIAStab_b90,2,FUN=mean)

B2unpwmse_b90<-apply(B2unBIAStab_b90^2,2,FUN=mean)

save(B2unALPHAtab_w10,file="B2unALPHAtab_w10.RData")

save(B2unESTMtab_w10,file="B2unESTMtab_w10.RData")

save(B2unPOWERtab_w10,file="B2unPOWERtab_w10.RData")

save(B2unBIAStab_w10,file="B2unBIAStab_w10.RData")

#calculate mean, bias, se and mse for type I error and power

B2unalpha_w10<-apply(B2unALPHAtab_w10<0.05,2,FUN=mean)

B2unaphse_w10<-apply(B2unESTMtab_w10,2,FUN=sd)

B2unaphbias_w10<-apply(B2unESTMtab_w10,2,FUN=mean)

B2unaphmse_w10<-apply(B2unESTMtab_w10^2,2,FUN=mean)

B2unpower_w10<-apply(B2unPOWERtab_w10<0.05,2,FUN=mean)

B2unpwse_w10<-apply(B2unBIAStab_w10,2,FUN=sd)

B2unpwbias_w10<-apply(B2unBIAStab_w10,2,FUN=mean)

B2unpwmse_w10<-apply(B2unBIAStab_w10^2,2,FUN=mean)

save(B2unALPHAtab_w20,file="B2unALPHAtab_w20.RData")

save(B2unESTMtab_w20,file="B2unESTMtab_w20.RData")

save(B2unPOWERtab_w20,file="B2unPOWERtab_w20.RData")

save(B2unBIAStab_w20,file="B2unBIAStab_w20.RData")

#calculate mean, bias, se and mse for type I error and power

B2unalpha_w20<-apply(B2unALPHAtab_w20<0.05,2,FUN=mean)

B2unaphse_w20<-apply(B2unESTMtab_w20,2,FUN=sd)

B2unaphbias_w20<-apply(B2unESTMtab_w20,2,FUN=mean)

B2unaphmse_w20<-apply(B2unESTMtab_w20^2,2,FUN=mean)

B2unpower_w20<-apply(B2unPOWERtab_w20<0.05,2,FUN=mean)

B2unpwse_w20<-apply(B2unBIAStab_w20,2,FUN=sd)

B2unpwbias_w20<-apply(B2unBIAStab_w20,2,FUN=mean)

B2unpwmse_w20<-apply(B2unBIAStab_w20^2,2,FUN=mean)

save(B2unALPHAtab_w30,file="B2unALPHAtab_w30.RData")

save(B2unESTMtab_w30,file="B2unESTMtab_w30.RData")

save(B2unPOWERtab_w30,file="B2unPOWERtab_w30.RData")

save(B2unBIAStab_w30,file="B2unBIAStab_w30.RData")

#calculate mean, bias, se and mse for type I error and power

B2unalpha_w30<-apply(B2unALPHAtab_w30<0.05,2,FUN=mean)

B2unaphse_w30<-apply(B2unESTMtab_w30,2,FUN=sd)

B2unaphbias_w30<-apply(B2unESTMtab_w30,2,FUN=mean)

B2unaphmse_w30<-apply(B2unESTMtab_w30^2,2,FUN=mean)

B2unpower_w30<-apply(B2unPOWERtab_w30<0.05,2,FUN=mean)

B2unpwse_w30<-apply(B2unBIAStab_w30,2,FUN=sd)

B2unpwbias_w30<-apply(B2unBIAStab_w30,2,FUN=mean)

B2unpwmse_w30<-apply(B2unBIAStab_w30^2,2,FUN=mean)

save(B2unALPHAtab_w40,file="B2unALPHAtab_w40.RData")

save(B2unESTMtab_w40,file="B2unESTMtab_w40.RData")

save(B2unPOWERtab_w40,file="B2unPOWERtab_w40.RData")

save(B2unBIAStab_w40,file="B2unBIAStab_w40.RData")

#calculate mean, bias, se and mse for type I error and power

B2unalpha_w40<-apply(B2unALPHAtab_w40<0.05,2,FUN=mean)

B2unaphse_w40<-apply(B2unESTMtab_w40,2,FUN=sd)

B2unaphbias_w40<-apply(B2unESTMtab_w40,2,FUN=mean)

B2unaphmse_w40<-apply(B2unESTMtab_w40^2,2,FUN=mean)

B2unpower_w40<-apply(B2unPOWERtab_w40<0.05,2,FUN=mean)

B2unpwse_w40<-apply(B2unBIAStab_w40,2,FUN=sd)

B2unpwbias_w40<-apply(B2unBIAStab_w40,2,FUN=mean)

B2unpwmse_w40<-apply(B2unBIAStab_w40^2,2,FUN=mean)

save(B2unALPHAtab_w50,file="B2unALPHAtab_w50.RData")

save(B2unESTMtab_w50,file="B2unESTMtab_w50.RData")

save(B2unPOWERtab_w50,file="B2unPOWERtab_w50.RData")

save(B2unBIAStab_w50,file="B2unBIAStab_w50.RData")

#calculate mean, bias, se and mse for type I error and power

B2unalpha_w50<-apply(B2unALPHAtab_w50<0.05,2,FUN=mean)

B2unaphse_w50<-apply(B2unESTMtab_w50,2,FUN=sd)

B2unaphbias_w50<-apply(B2unESTMtab_w50,2,FUN=mean)

B2unaphmse_w50<-apply(B2unESTMtab_w50^2,2,FUN=mean)

B2unpower_w50<-apply(B2unPOWERtab_w50<0.05,2,FUN=mean)

B2unpwse_w50<-apply(B2unBIAStab_w50,2,FUN=sd)

B2unpwbias_w50<-apply(B2unBIAStab_w50,2,FUN=mean)

B2unpwmse_w50<-apply(B2unBIAStab_w50^2,2,FUN=mean)

save(B2unALPHAtab_w60,file="B2unALPHAtab_w60.RData")

save(B2unESTMtab_w60,file="B2unESTMtab_w60.RData")

save(B2unPOWERtab_w60,file="B2unPOWERtab_w60.RData")

save(B2unBIAStab_w60,file="B2unBIAStab_w60.RData")

#calculate mean, bias, se and mse for type I error and power

B2unalpha_w60<-apply(B2unALPHAtab_w60<0.05,2,FUN=mean)

B2unaphse_w60<-apply(B2unESTMtab_w60,2,FUN=sd)

B2unaphbias_w60<-apply(B2unESTMtab_w60,2,FUN=mean)

B2unaphmse_w60<-apply(B2unESTMtab_w60^2,2,FUN=mean)

B2unpower_w60<-apply(B2unPOWERtab_w60<0.05,2,FUN=mean)

B2unpwse_w60<-apply(B2unBIAStab_w60,2,FUN=sd)

B2unpwbias_w60<-apply(B2unBIAStab_w60,2,FUN=mean)

B2unpwmse_w60<-apply(B2unBIAStab_w60^2,2,FUN=mean)

save(B2unALPHAtab_w70,file="B2unALPHAtab_w70.RData")

save(B2unESTMtab_w70,file="B2unESTMtab_w70.RData")

save(B2unPOWERtab_w70,file="B2unPOWERtab_w70.RData")

save(B2unBIAStab_w70,file="B2unBIAStab_w70.RData")

#calculate mean, bias, se and mse for type I error and power

B2unalpha_w70<-apply(B2unALPHAtab_w70<0.05,2,FUN=mean)

B2unaphse_w70<-apply(B2unESTMtab_w70,2,FUN=sd)

B2unaphbias_w70<-apply(B2unESTMtab_w70,2,FUN=mean)

B2unaphmse_w70<-apply(B2unESTMtab_w70^2,2,FUN=mean)

B2unpower_w70<-apply(B2unPOWERtab_w70<0.05,2,FUN=mean)

B2unpwse_w70<-apply(B2unBIAStab_w70,2,FUN=sd)

B2unpwbias_w70<-apply(B2unBIAStab_w70,2,FUN=mean)

B2unpwmse_w70<-apply(B2unBIAStab_w70^2,2,FUN=mean)

save(B2unALPHAtab_w80,file="B2unALPHAtab_w80.RData")

save(B2unESTMtab_w80,file="B2unESTMtab_w80.RData")

save(B2unPOWERtab_w80,file="B2unPOWERtab_w80.RData")

save(B2unBIAStab_w80,file="B2unBIAStab_w80.RData")

#calculate mean, bias, se and mse for type I error and power

B2unalpha_w80<-apply(B2unALPHAtab_w80<0.05,2,FUN=mean)

B2unaphse_w80<-apply(B2unESTMtab_w80,2,FUN=sd)

B2unaphbias_w80<-apply(B2unESTMtab_w80,2,FUN=mean)

B2unaphmse_w80<-apply(B2unESTMtab_w80^2,2,FUN=mean)

B2unpower_w80<-apply(B2unPOWERtab_w80<0.05,2,FUN=mean)

B2unpwse_w80<-apply(B2unBIAStab_w80,2,FUN=sd)

B2unpwbias_w80<-apply(B2unBIAStab_w80,2,FUN=mean)

B2unpwmse_w80<-apply(B2unBIAStab_w80^2,2,FUN=mean)

save(B2unALPHAtab_w90,file="B2unALPHAtab_w90.RData")

save(B2unESTMtab_w90,file="B2unESTMtab_w90.RData")

save(B2unPOWERtab_w90,file="B2unPOWERtab_w90.RData")

save(B2unBIAStab_w90,file="B2unBIAStab_w90.RData")

#calculate mean, bias, se and mse for type I error and power

B2unalpha_w90<-apply(B2unALPHAtab_w90<0.05,2,FUN=mean)

B2unaphse_w90<-apply(B2unESTMtab_w90,2,FUN=sd)

B2unaphbias_w90<-apply(B2unESTMtab_w90,2,FUN=mean)

B2unaphmse_w90<-apply(B2unESTMtab_w90^2,2,FUN=mean)

B2unpower_w90<-apply(B2unPOWERtab_w90<0.05,2,FUN=mean)

B2unpwse_w90<-apply(B2unBIAStab_w90,2,FUN=sd)

B2unpwbias_w90<-apply(B2unBIAStab_w90,2,FUN=mean)

B2unpwmse_w90<-apply(B2unBIAStab_w90^2,2,FUN=mean)

#save

save(B2unalpha_b10,file="B2unalpha_b10.RData")

save(B2unaphse_b10,file="B2unaphse_b10.RData")

save(B2unaphbias_b10,file="B2unaphbias_b10.RData")

save(B2unaphmse_b10,file="B2unaphmse_b10.RData")

save(B2unpower_b10,file="B2unpower_b10.Rdata")

save(B2unpwse_b10,file="B2unpwse_b10.RData")

save(B2unpwbias_b10,file="B2unpwbias_b10.RData")

save(B2unpwmse_b10,file="B2unpwmse_b10.RData")

save(B2unalpha_b20,file="B2unalpha_b20.RData")

save(B2unaphse_b20,file="B2unaphse_b20.RData")

save(B2unaphbias_b20,file="B2unaphbias_b20.RData")

save(B2unaphmse_b20,file="B2unaphmse_b20.RData")

save(B2unpower_b20,file="B2unpower_b20.Rdata")

save(B2unpwse_b20,file="B2unpwse_b20.RData")

save(B2unpwbias_b20,file="B2unpwbias_b20.RData")

save(B2unpwmse_b20,file="B2unpwmse_b20.RData")

save(B2unalpha_b30,file="B2unalpha_b30.RData")

save(B2unaphse_b30,file="B2unaphse_b30.RData")

save(B2unaphbias_b30,file="B2unaphbias_b30.RData")

save(B2unaphmse_b30,file="B2unaphmse_b30.RData")

save(B2unpower_b30,file="B2unpower_b30.Rdata")

save(B2unpwse_b30,file="B2unpwse_b30.RData")

save(B2unpwbias_b30,file="B2unpwbias_b30.RData")

save(B2unpwmse_b30,file="B2unpwmse_b30.RData")

save(B2unalpha_b40,file="B2unalpha_b40.RData")

save(B2unaphse_b40,file="B2unaphse_b40.RData")

save(B2unaphbias_b40,file="B2unaphbias_b40.RData")

save(B2unaphmse_b40,file="B2unaphmse_b40.RData")

save(B2unpower_b40,file="B2unpower_b40.Rdata")

save(B2unpwse_b40,file="B2unpwse_b40.RData")

save(B2unpwbias_b40,file="B2unpwbias_b40.RData")

save(B2unpwmse_b40,file="B2unpwmse_b40.RData")

save(B2unalpha_b50,file="B2unalpha_b50.RData")

save(B2unaphse_b50,file="B2unaphse_b50.RData")

save(B2unaphbias_b50,file="B2unaphbias_b50.RData")

save(B2unaphmse_b50,file="B2unaphmse_b50.RData")

save(B2unpower_b50,file="B2unpower_b50.Rdata")

save(B2unpwse_b50,file="B2unpwse_b50.RData")

save(B2unpwbias_b50,file="B2unpwbias_b50.RData")

save(B2unpwmse_b50,file="B2unpwmse_b50.RData")

save(B2unalpha_b60,file="B2unalpha_b60.RData")

save(B2unaphse_b60,file="B2unaphse_b60.RData")

save(B2unaphbias_b60,file="B2unaphbias_b60.RData")

save(B2unaphmse_b60,file="B2unaphmse_b60.RData")

save(B2unpower_b60,file="B2unpower_b60.Rdata")

save(B2unpwse_b60,file="B2unpwse_b60.RData")

save(B2unpwbias_b60,file="B2unpwbias_b60.RData")

save(B2unpwmse_b60,file="B2unpwmse_b60.RData")

save(B2unalpha_b70,file="B2unalpha_b70.RData")

save(B2unaphse_b70,file="B2unaphse_b70.RData")

save(B2unaphbias_b70,file="B2unaphbias_b70.RData")

save(B2unaphmse_b70,file="B2unaphmse_b70.RData")

save(B2unpower_b70,file="B2unpower_b70.Rdata")

save(B2unpwse_b70,file="B2unpwse_b70.RData")

save(B2unpwbias_b70,file="B2unpwbias_b70.RData")

save(B2unpwmse_b70,file="B2unpwmse_b70.RData")

save(B2unalpha_b80,file="B2unalpha_b80.RData")

save(B2unaphse_b80,file="B2unaphse_b80.RData")

save(B2unaphbias_b80,file="B2unaphbias_b80.RData")

save(B2unaphmse_b80,file="B2unaphmse_b80.RData")

save(B2unpower_b80,file="B2unpower_b80.Rdata")

save(B2unpwse_b80,file="B2unpwse_b80.RData")

save(B2unpwbias_b80,file="B2unpwbias_b80.RData")

save(B2unpwmse_b80,file="B2unpwmse_b80.RData")

save(B2unalpha_b90,file="B2unalpha_b90.RData")

save(B2unaphse_b90,file="B2unaphse_b90.RData")

save(B2unaphbias_b90,file="B2unaphbias_b90.RData")

save(B2unaphmse_b90,file="B2unaphmse_b90.RData")

save(B2unpower_b90,file="B2unpower_b90.Rdata")

save(B2unpwse_b90,file="B2unpwse_b90.RData")

save(B2unpwbias_b90,file="B2unpwbias_b90.RData")

save(B2unpwmse_b90,file="B2unpwmse_b90.RData")

save(B2unalpha_w10,file="B2unalpha_w10.RData")

save(B2unaphse_w10,file="B2unaphse_w10.RData")

save(B2unaphbias_w10,file="B2unaphbias_w10.RData")

save(B2unaphmse_w10,file="B2unaphmse_w10.RData")

save(B2unpower_w10,file="B2unpower_w10.Rdata")

save(B2unpwse_w10,file="B2unpwse_w10.RData")

save(B2unpwbias_w10,file="B2unpwbias_w10.RData")

save(B2unpwmse_w10,file="B2unpwmse_w10.RData")

save(B2unalpha_w20,file="B2unalpha_w20.RData")

save(B2unaphse_w20,file="B2unaphse_w20.RData")

save(B2unaphbias_w20,file="B2unaphbias_w20.RData")

save(B2unaphmse_w20,file="B2unaphmse_w20.RData")

save(B2unpower_w20,file="B2unpower_w20.Rdata")

save(B2unpwse_w20,file="B2unpwse_w20.RData")

save(B2unpwbias_w20,file="B2unpwbias_w20.RData")

save(B2unpwmse_w20,file="B2unpwmse_w20.RData")

save(B2unalpha_w30,file="B2unalpha_w30.RData")

save(B2unaphse_w30,file="B2unaphse_w30.RData")

save(B2unaphbias_w30,file="B2unaphbias_w30.RData")

save(B2unaphmse_w30,file="B2unaphmse_w30.RData")

save(B2unpower_w30,file="B2unpower_w30.Rdata")

save(B2unpwse_w30,file="B2unpwse_w30.RData")

save(B2unpwbias_w30,file="B2unpwbias_w30.RData")

save(B2unpwmse_w30,file="B2unpwmse_w30.RData")

save(B2unalpha_w40,file="B2unalpha_w40.RData")

save(B2unaphse_w40,file="B2unaphse_w40.RData")

save(B2unaphbias_w40,file="B2unaphbias_w40.RData")

save(B2unaphmse_w40,file="B2unaphmse_w40.RData")

save(B2unpower_w40,file="B2unpower_w40.Rdata")

save(B2unpwse_w40,file="B2unpwse_w40.RData")

save(B2unpwbias_w40,file="B2unpwbias_w40.RData")

save(B2unpwmse_w40,file="B2unpwmse_w40.RData")

save(B2unalpha_w50,file="B2unalpha_w50.RData")

save(B2unaphse_w50,file="B2unaphse_w50.RData")

save(B2unaphbias_w50,file="B2unaphbias_w50.RData")

save(B2unaphmse_w50,file="B2unaphmse_w50.RData")

save(B2unpower_w50,file="B2unpower_w50.Rdata")

save(B2unpwse_w50,file="B2unpwse_w50.RData")

save(B2unpwbias_w50,file="B2unpwbias_w50.RData")

save(B2unpwmse_w50,file="B2unpwmse_w50.RData")

save(B2unalpha_w60,file="B2unalpha_w60.RData")

save(B2unaphse_w60,file="B2unaphse_w60.RData")

save(B2unaphbias_w60,file="B2unaphbias_w60.RData")

save(B2unaphmse_w60,file="B2unaphmse_w60.RData")

save(B2unpower_w60,file="B2unpower_w60.Rdata")

save(B2unpwse_w60,file="B2unpwse_w60.RData")

save(B2unpwbias_w60,file="B2unpwbias_w60.RData")

save(B2unpwmse_w60,file="B2unpwmse_w60.RData")

save(B2unalpha_w70,file="B2unalpha_w70.RData")

save(B2unaphse_w70,file="B2unaphse_w70.RData")

save(B2unaphbias_w70,file="B2unaphbias_w70.RData")

save(B2unaphmse_w70,file="B2unaphmse_w70.RData")

save(B2unpower_w70,file="B2unpower_w70.Rdata")

save(B2unpwse_w70,file="B2unpwse_w70.RData")

save(B2unpwbias_w70,file="B2unpwbias_w70.RData")

save(B2unpwmse_w70,file="B2unpwmse_w70.RData")

save(B2unalpha_w80,file="B2unalpha_w80.RData")

save(B2unaphse_w80,file="B2unaphse_w80.RData")

save(B2unaphbias_w80,file="B2unaphbias_w80.RData")

save(B2unaphmse_w80,file="B2unaphmse_w80.RData")

save(B2unpower_w80,file="B2unpower_w80.Rdata")

save(B2unpwse_w80,file="B2unpwse_w80.RData")

save(B2unpwbias_w80,file="B2unpwbias_w80.RData")

save(B2unpwmse_w80,file="B2unpwmse_w80.RData")

save(B2unalpha_w90,file="B2unalpha_w90.RData")

save(B2unaphse_w90,file="B2unaphse_w90.RData")

save(B2unaphbias_w90,file="B2unaphbias_w90.RData")

save(B2unaphmse_w90,file="B2unaphmse_w90.RData")

save(B2unpower_w90,file="B2unpower_w90.Rdata")

save(B2unpwse_w90,file="B2unpwse_w90.RData")

save(B2unpwbias_w90,file="B2unpwbias_w90.RData")

save(B2unpwmse_w90,file="B2unpwmse_w90.RData")

###

#calculate mean, bias, se and mse for type I error and power

B2B1s1adalpha<-apply(B2s1adALPHAtab_names[[1]]<0.05,2,FUN=mean)

B2B1s1adaphse<-apply(B2s1adESTMtab_names[[1]],2,FUN=sd)

B2B1s1adaphbias<-apply(B2s1adESTMtab_names[[1]],2,FUN=mean)

B2B1s1adaphmse<-apply(B2s1adESTMtab_names[[1]]^2,2,FUN=mean)

B2B1s1adpower<-apply(B2s1adPOWERtab_names[[1]]<0.05,2,FUN=mean)

B2B1s1adpwse<-apply(B2s1adBIAStab_names[[1]],2,FUN=sd)

B2B1s1adpwbias<-apply(B2s1adBIAStab_names[[1]],2,FUN=mean)

B2B1s1adpwmse<-apply(B2s1adBIAStab_names[[1]]^2,2,FUN=mean)

#calculate mean, bias, se and mse for type I error and power

B2B2s1adalpha<-apply(B2s1adALPHAtab_names[[2]]<0.05,2,FUN=mean)

B2B2s1adaphse<-apply(B2s1adESTMtab_names[[2]],2,FUN=sd)

B2B2s1adaphbias<-apply(B2s1adESTMtab_names[[2]],2,FUN=mean)

B2B2s1adaphmse<-apply(B2s1adESTMtab_names[[2]]^2,2,FUN=mean)

B2B2s1adpower<-apply(B2s1adPOWERtab_names[[2]]<0.05,2,FUN=mean)

B2B2s1adpwse<-apply(B2s1adBIAStab_names[[2]],2,FUN=sd)

B2B2s1adpwbias<-apply(B2s1adBIAStab_names[[2]],2,FUN=mean)

B2B2s1adpwmse<-apply(B2s1adBIAStab_names[[2]]^2,2,FUN=mean)

#calculate mean, bias, se and mse for type I error and power

B2B3s1adalpha<-apply(B2s1adALPHAtab_names[[3]]<0.05,2,FUN=mean)

B2B3s1adaphse<-apply(B2s1adESTMtab_names[[3]],2,FUN=sd)

B2B3s1adaphbias<-apply(B2s1adESTMtab_names[[3]],2,FUN=mean)

B2B3s1adaphmse<-apply(B2s1adESTMtab_names[[3]]^2,2,FUN=mean)

B2B3s1adpower<-apply(B2s1adPOWERtab_names[[3]]<0.05,2,FUN=mean)

B2B3s1adpwse<-apply(B2s1adBIAStab_names[[3]],2,FUN=sd)

B2B3s1adpwbias<-apply(B2s1adBIAStab_names[[3]],2,FUN=mean)

B2B3s1adpwmse<-apply(B2s1adBIAStab_names[[3]]^2,2,FUN=mean)

#calculate mean, bias, se and mse for type I error and power

B2B4s1adalpha<-apply(B2s1adALPHAtab_names[[4]]<0.05,2,FUN=mean)

B2B4s1adaphse<-apply(B2s1adESTMtab_names[[4]],2,FUN=sd)

B2B4s1adaphbias<-apply(B2s1adESTMtab_names[[4]],2,FUN=mean)

B2B4s1adaphmse<-apply(B2s1adESTMtab_names[[4]]^2,2,FUN=mean)

B2B4s1adpower<-apply(B2s1adPOWERtab_names[[4]]<0.05,2,FUN=mean)

B2B4s1adpwse<-apply(B2s1adBIAStab_names[[4]],2,FUN=sd)

B2B4s1adpwbias<-apply(B2s1adBIAStab_names[[4]],2,FUN=mean)

B2B4s1adpwmse<-apply(B2s1adBIAStab_names[[4]]^2,2,FUN=mean)

#calculate mean, bias, se and mse for type I error and power

B2B1s2adalpha<-apply(B2s2adALPHAtab_names[[1]]<0.05,2,FUN=mean)

B2B1s2adaphse<-apply(B2s2adESTMtab_names[[1]],2,FUN=sd)

B2B1s2adaphbias<-apply(B2s2adESTMtab_names[[1]],2,FUN=mean)

B2B1s2adaphmse<-apply(B2s2adESTMtab_names[[1]]^2,2,FUN=mean)

B2B1s2adpower<-apply(B2s2adPOWERtab_names[[1]]<0.05,2,FUN=mean)

B2B1s2adpwse<-apply(B2s2adBIAStab_names[[1]],2,FUN=sd)

B2B1s2adpwbias<-apply(B2s2adBIAStab_names[[1]],2,FUN=mean)

B2B1s2adpwmse<-apply(B2s2adBIAStab_names[[1]]^2,2,FUN=mean)

#calculate mean, bias, se and mse for type I error and power

B2B2s2adalpha<-apply(B2s2adALPHAtab_names[[2]]<0.05,2,FUN=mean)

B2B2s2adaphse<-apply(B2s2adESTMtab_names[[2]],2,FUN=sd)

B2B2s2adaphbias<-apply(B2s2adESTMtab_names[[2]],2,FUN=mean)

B2B2s2adaphmse<-apply(B2s2adESTMtab_names[[2]]^2,2,FUN=mean)

B2B2s2adpower<-apply(B2s2adPOWERtab_names[[2]]<0.05,2,FUN=mean)

B2B2s2adpwse<-apply(B2s2adBIAStab_names[[2]],2,FUN=sd)

B2B2s2adpwbias<-apply(B2s2adBIAStab_names[[2]],2,FUN=mean)

B2B2s2adpwmse<-apply(B2s2adBIAStab_names[[2]]^2,2,FUN=mean)

#calculate mean, bias, se and mse for type I error and power

B2B3s2adalpha<-apply(B2s2adALPHAtab_names[[3]]<0.05,2,FUN=mean)

B2B3s2adaphse<-apply(B2s2adESTMtab_names[[3]],2,FUN=sd)

B2B3s2adaphbias<-apply(B2s2adESTMtab_names[[3]],2,FUN=mean)

B2B3s2adaphmse<-apply(B2s2adESTMtab_names[[3]]^2,2,FUN=mean)

B2B3s2adpower<-apply(B2s2adPOWERtab_names[[3]]<0.05,2,FUN=mean)

B2B3s2adpwse<-apply(B2s2adBIAStab_names[[3]],2,FUN=sd)

B2B3s2adpwbias<-apply(B2s2adBIAStab_names[[3]],2,FUN=mean)

B2B3s2adpwmse<-apply(B2s2adBIAStab_names[[3]]^2,2,FUN=mean)

#calculate mean, bias, se and mse for type I error and power

B2B4s2adalpha<-apply(B2s2adALPHAtab_names[[4]]<0.05,2,FUN=mean)

B2B4s2adaphse<-apply(B2s2adESTMtab_names[[4]],2,FUN=sd)

B2B4s2adaphbias<-apply(B2s2adESTMtab_names[[4]],2,FUN=mean)

B2B4s2adaphmse<-apply(B2s2adESTMtab_names[[4]]^2,2,FUN=mean)

B2B4s2adpower<-apply(B2s2adPOWERtab_names[[4]]<0.05,2,FUN=mean)

B2B4s2adpwse<-apply(B2s2adBIAStab_names[[4]],2,FUN=sd)

B2B4s2adpwbias<-apply(B2s2adBIAStab_names[[4]],2,FUN=mean)

B2B4s2adpwmse<-apply(B2s2adBIAStab_names[[4]]^2,2,FUN=mean)

#calculate mean, bias, se and mse for type I error and power

B2B1s3adalpha<-apply(B2s3adALPHAtab_names[[1]]<0.05,2,FUN=mean)

B2B1s3adaphse<-apply(B2s3adESTMtab_names[[1]],2,FUN=sd)

B2B1s3adaphbias<-apply(B2s3adESTMtab_names[[1]],2,FUN=mean)

B2B1s3adaphmse<-apply(B2s3adESTMtab_names[[1]]^2,2,FUN=mean)

B2B1s3adpower<-apply(B2s3adPOWERtab_names[[1]]<0.05,2,FUN=mean)

B2B1s3adpwse<-apply(B2s3adBIAStab_names[[1]],2,FUN=sd)

B2B1s3adpwbias<-apply(B2s3adBIAStab_names[[1]],2,FUN=mean)

B2B1s3adpwmse<-apply(B2s3adBIAStab_names[[1]]^2,2,FUN=mean)

#calculate mean, bias, se and mse for type I error and power

B2B2s3adalpha<-apply(B2s3adALPHAtab_names[[2]]<0.05,2,FUN=mean)

B2B2s3adaphse<-apply(B2s3adESTMtab_names[[2]],2,FUN=sd)

B2B2s3adaphbias<-apply(B2s3adESTMtab_names[[2]],2,FUN=mean)

B2B2s3adaphmse<-apply(B2s3adESTMtab_names[[2]]^2,2,FUN=mean)

B2B2s3adpower<-apply(B2s3adPOWERtab_names[[2]]<0.05,2,FUN=mean)

B2B2s3adpwse<-apply(B2s3adBIAStab_names[[2]],2,FUN=sd)

B2B2s3adpwbias<-apply(B2s3adBIAStab_names[[2]],2,FUN=mean)

B2B2s3adpwmse<-apply(B2s3adBIAStab_names[[2]]^2,2,FUN=mean)

#calculate mean, bias, se and mse for type I error and power

B2B3s3adalpha<-apply(B2s3adALPHAtab_names[[3]]<0.05,2,FUN=mean)

B2B3s3adaphse<-apply(B2s3adESTMtab_names[[3]],2,FUN=sd)

B2B3s3adaphbias<-apply(B2s3adESTMtab_names[[3]],2,FUN=mean)

B2B3s3adaphmse<-apply(B2s3adESTMtab_names[[3]]^2,2,FUN=mean)

B2B3s3adpower<-apply(B2s3adPOWERtab_names[[3]]<0.05,2,FUN=mean)

B2B3s3adpwse<-apply(B2s3adBIAStab_names[[3]],2,FUN=sd)

B2B3s3adpwbias<-apply(B2s3adBIAStab_names[[3]],2,FUN=mean)

B2B3s3adpwmse<-apply(B2s3adBIAStab_names[[3]]^2,2,FUN=mean)

#calculate mean, bias, se and mse for type I error and power

B2B4s3adalpha<-apply(B2s3adALPHAtab_names[[4]]<0.05,2,FUN=mean)

B2B4s3adaphse<-apply(B2s3adESTMtab_names[[4]],2,FUN=sd)

B2B4s3adaphbias<-apply(B2s3adESTMtab_names[[4]],2,FUN=mean)

B2B4s3adaphmse<-apply(B2s3adESTMtab_names[[4]]^2,2,FUN=mean)

B2B4s3adpower<-apply(B2s3adPOWERtab_names[[4]]<0.05,2,FUN=mean)

B2B4s3adpwse<-apply(B2s3adBIAStab_names[[4]],2,FUN=sd)

B2B4s3adpwbias<-apply(B2s3adBIAStab_names[[4]],2,FUN=mean)

B2B4s3adpwmse<-apply(B2s3adBIAStab_names[[4]]^2,2,FUN=mean)

#calculate mean, bias, se and mse for type I error and power

B2B1s4adalpha<-apply(B2s4adALPHAtab_names[[1]]<0.05,2,FUN=mean)

B2B1s4adaphse<-apply(B2s4adESTMtab_names[[1]],2,FUN=sd)

B2B1s4adaphbias<-apply(B2s4adESTMtab_names[[1]],2,FUN=mean)

B2B1s4adaphmse<-apply(B2s4adESTMtab_names[[1]]^2,2,FUN=mean)

B2B1s4adpower<-apply(B2s4adPOWERtab_names[[1]]<0.05,2,FUN=mean)

B2B1s4adpwse<-apply(B2s4adBIAStab_names[[1]],2,FUN=sd)

B2B1s4adpwbias<-apply(B2s4adBIAStab_names[[1]],2,FUN=mean)

B2B1s4adpwmse<-apply(B2s4adBIAStab_names[[1]]^2,2,FUN=mean)

#calculate mean, bias, se and mse for type I error and power

B2B2s4adalpha<-apply(B2s4adALPHAtab_names[[2]]<0.05,2,FUN=mean)

B2B2s4adaphse<-apply(B2s4adESTMtab_names[[2]],2,FUN=sd)

B2B2s4adaphbias<-apply(B2s4adESTMtab_names[[2]],2,FUN=mean)

B2B2s4adaphmse<-apply(B2s4adESTMtab_names[[2]]^2,2,FUN=mean)

B2B2s4adpower<-apply(B2s4adPOWERtab_names[[2]]<0.05,2,FUN=mean)

B2B2s4adpwse<-apply(B2s4adBIAStab_names[[2]],2,FUN=sd)

B2B2s4adpwbias<-apply(B2s4adBIAStab_names[[2]],2,FUN=mean)

B2B2s4adpwmse<-apply(B2s4adBIAStab_names[[2]]^2,2,FUN=mean)

#calculate mean, bias, se and mse for type I error and power

B2B3s4adalpha<-apply(B2s4adALPHAtab_names[[3]]<0.05,2,FUN=mean)

B2B3s4adaphse<-apply(B2s4adESTMtab_names[[3]],2,FUN=sd)

B2B3s4adaphbias<-apply(B2s4adESTMtab_names[[3]],2,FUN=mean)

B2B3s4adaphmse<-apply(B2s4adESTMtab_names[[3]]^2,2,FUN=mean)

B2B3s4adpower<-apply(B2s4adPOWERtab_names[[3]]<0.05,2,FUN=mean)

B2B3s4adpwse<-apply(B2s4adBIAStab_names[[3]],2,FUN=sd)

B2B3s4adpwbias<-apply(B2s4adBIAStab_names[[3]],2,FUN=mean)

B2B3s4adpwmse<-apply(B2s4adBIAStab_names[[3]]^2,2,FUN=mean)

#save(B2s4adALPHAtab_names[[4]],file="B2B4s4adALPHAtab.RData")

#save(B2s4adESTMtab_names[[4]],file="B2B4s4adESTMtab.RData")

#save(B2s4adPOWERtab_names[[4]],file="B2B4s4adPOWERtab.RData")

#save(B2s4adBIAStab_names[[4]],file="B2B4s4adBIAStab.RData")

#calculate mean, bias, se and mse for type I error and power

B2B4s4adalpha<-apply(B2s4adALPHAtab_names[[4]]<0.05,2,FUN=mean)

B2B4s4adaphse<-apply(B2s4adESTMtab_names[[4]],2,FUN=sd)

B2B4s4adaphbias<-apply(B2s4adESTMtab_names[[4]],2,FUN=mean)

B2B4s4adaphmse<-apply(B2s4adESTMtab_names[[4]]^2,2,FUN=mean)

B2B4s4adpower<-apply(B2s4adPOWERtab_names[[4]]<0.05,2,FUN=mean)

B2B4s4adpwse<-apply(B2s4adBIAStab_names[[4]],2,FUN=sd)

B2B4s4adpwbias<-apply(B2s4adBIAStab_names[[4]],2,FUN=mean)

B2B4s4adpwmse<-apply(B2s4adBIAStab_names[[4]]^2,2,FUN=mean)

# save

save(B2B1s1adalpha,file="B2B1s1adalpha.RData")

save(B2B1s1adaphse,file="B2B1s1adaphse.RData")

save(B2B1s1adaphbias,file="B2B1s1adaphbias.RData")

save(B2B1s1adaphmse,file="B2B1s1adaphmse.RData")

save(B2B1s1adpower,file="B2B1s1adpower.Rdata")

save(B2B1s1adpwse,file="B2B1s1adpwse.RData")

save(B2B1s1adpwbias,file="B2B1s1adpwbias.RData")

save(B2B1s1adpwmse,file="B2B1s1adpwmse.RData")

save(B2B2s1adalpha,file="B2B2s1adalpha.RData")

save(B2B2s1adaphse,file="B2B2s1adaphse.RData")

save(B2B2s1adaphbias,file="B2B2s1adaphbias.RData")

save(B2B2s1adaphmse,file="B2B2s1adaphmse.RData")

save(B2B2s1adpower,file="B2B2s1adpower.Rdata")

save(B2B2s1adpwse,file="B2B2s1adpwse.RData")

save(B2B2s1adpwbias,file="B2B2s1adpwbias.RData")

save(B2B2s1adpwmse,file="B2B2s1adpwmse.RData")

save(B2B3s1adalpha,file="B2B3s1adalpha.RData")

save(B2B3s1adaphse,file="B2B3s1adaphse.RData")

save(B2B3s1adaphbias,file="B2B3s1adaphbias.RData")

save(B2B3s1adaphmse,file="B2B3s1adaphmse.RData")

save(B2B3s1adpower,file="B2B3s1adpower.Rdata")

save(B2B3s1adpwse,file="B2B3s1adpwse.RData")

save(B2B3s1adpwbias,file="B2B3s1adpwbias.RData")

save(B2B3s1adpwmse,file="B2B3s1adpwmse.RData")

save(B2B4s1adalpha,file="B2B4s1adalpha.RData")

save(B2B4s1adaphse,file="B2B4s1adaphse.RData")

save(B2B4s1adaphbias,file="B2B4s1adaphbias.RData")

save(B2B4s1adaphmse,file="B2B4s1adaphmse.RData")

save(B2B4s1adpower,file="B2B4s1adpower.Rdata")

save(B2B4s1adpwse,file="B2B4s1adpwse.RData")

save(B2B4s1adpwbias,file="B2B4s1adpwbias.RData")

save(B2B4s1adpwmse,file="B2B4s1adpwmse.RData")

save(B2B1s2adalpha,file="B2B1s2adalpha.RData")

save(B2B1s2adaphse,file="B2B1s2adaphse.RData")

save(B2B1s2adaphbias,file="B2B1s2adaphbias.RData")

save(B2B1s2adaphmse,file="B2B1s2adaphmse.RData")

save(B2B1s2adpower,file="B2B1s2adpower.Rdata")

save(B2B1s2adpwse,file="B2B1s2adpwse.RData")

save(B2B1s2adpwbias,file="B2B1s2adpwbias.RData")

save(B2B1s2adpwmse,file="B2B1s2adpwmse.RData")

save(B2B2s2adalpha,file="B2B2s2adalpha.RData")

save(B2B2s2adaphse,file="B2B2s2adaphse.RData")

save(B2B2s2adaphbias,file="B2B2s2adaphbias.RData")

save(B2B2s2adaphmse,file="B2B2s2adaphmse.RData")

save(B2B2s2adpower,file="B2B2s2adpower.Rdata")

save(B2B2s2adpwse,file="B2B2s2adpwse.RData")

save(B2B2s2adpwbias,file="B2B2s2adpwbias.RData")

save(B2B2s2adpwmse,file="B2B2s2adpwmse.RData")

save(B2B3s2adalpha,file="B2B3s2adalpha.RData")

save(B2B3s2adaphse,file="B2B3s2adaphse.RData")

save(B2B3s2adaphbias,file="B2B3s2adaphbias.RData")

save(B2B3s2adaphmse,file="B2B3s2adaphmse.RData")

save(B2B3s2adpower,file="B2B3s2adpower.Rdata")

save(B2B3s2adpwse,file="B2B3s2adpwse.RData")

save(B2B3s2adpwbias,file="B2B3s2adpwbias.RData")

save(B2B3s2adpwmse,file="B2B3s2adpwmse.RData")

save(B2B4s2adalpha,file="B2B4s2adalpha.RData")

save(B2B4s2adaphse,file="B2B4s2adaphse.RData")

save(B2B4s2adaphbias,file="B2B4s2adaphbias.RData")

save(B2B4s2adaphmse,file="B2B4s2adaphmse.RData")

save(B2B4s2adpower,file="B2B4s2adpower.Rdata")

save(B2B4s2adpwse,file="B2B4s2adpwse.RData")

save(B2B4s2adpwbias,file="B2B4s2adpwbias.RData")

save(B2B4s2adpwmse,file="B2B4s2adpwmse.RData")

save(B2B1s3adalpha,file="B2B1s3adalpha.RData")

save(B2B1s3adaphse,file="B2B1s3adaphse.RData")

save(B2B1s3adaphbias,file="B2B1s3adaphbias.RData")

save(B2B1s3adaphmse,file="B2B1s3adaphmse.RData")

save(B2B1s3adpower,file="B2B1s3adpower.Rdata")

save(B2B1s3adpwse,file="B2B1s3adpwse.RData")

save(B2B1s3adpwbias,file="B2B1s3adpwbias.RData")

save(B2B1s3adpwmse,file="B2B1s3adpwmse.RData")

save(B2B2s3adalpha,file="B2B2s3adalpha.RData")

save(B2B2s3adaphse,file="B2B2s3adaphse.RData")

save(B2B2s3adaphbias,file="B2B2s3adaphbias.RData")

save(B2B2s3adaphmse,file="B2B2s3adaphmse.RData")

save(B2B2s3adpower,file="B2B2s3adpower.Rdata")

save(B2B2s3adpwse,file="B2B2s3adpwse.RData")

save(B2B2s3adpwbias,file="B2B2s3adpwbias.RData")

save(B2B2s3adpwmse,file="B2B2s3adpwmse.RData")

save(B2B3s3adalpha,file="B2B3s3adalpha.RData")

save(B2B3s3adaphse,file="B2B3s3adaphse.RData")

save(B2B3s3adaphbias,file="B2B3s3adaphbias.RData")

save(B2B3s3adaphmse,file="B2B3s3adaphmse.RData")

save(B2B3s3adpower,file="B2B3s3adpower.Rdata")

save(B2B3s3adpwse,file="B2B3s3adpwse.RData")

save(B2B3s3adpwbias,file="B2B3s3adpwbias.RData")

save(B2B3s3adpwmse,file="B2B3s3adpwmse.RData")

save(B2B4s3adalpha,file="B2B4s3adalpha.RData")

save(B2B4s3adaphse,file="B2B4s3adaphse.RData")

save(B2B4s3adaphbias,file="B2B4s3adaphbias.RData")

save(B2B4s3adaphmse,file="B2B4s3adaphmse.RData")

save(B2B4s3adpower,file="B2B4s3adpower.Rdata")

save(B2B4s3adpwse,file="B2B4s3adpwse.RData")

save(B2B4s3adpwbias,file="B2B4s3adpwbias.RData")

save(B2B4s3adpwmse,file="B2B4s3adpwmse.RData")

save(B2B1s4adalpha,file="B2B1s4adalpha.RData")

save(B2B1s4adaphse,file="B2B1s4adaphse.RData")

save(B2B1s4adaphbias,file="B2B1s4adaphbias.RData")

save(B2B1s4adaphmse,file="B2B1s4adaphmse.RData")

save(B2B1s4adpower,file="B2B1s4adpower.Rdata")

save(B2B1s4adpwse,file="B2B1s4adpwse.RData")

save(B2B1s4adpwbias,file="B2B1s4adpwbias.RData")

save(B2B1s4adpwmse,file="B2B1s4adpwmse.RData")

save(B2B2s4adalpha,file="B2B2s4adalpha.RData")

save(B2B2s4adaphse,file="B2B2s4adaphse.RData")

save(B2B2s4adaphbias,file="B2B2s4adaphbias.RData")

save(B2B2s4adaphmse,file="B2B2s4adaphmse.RData")

save(B2B2s4adpower,file="B2B2s4adpower.Rdata")

save(B2B2s4adpwse,file="B2B2s4adpwse.RData")

save(B2B2s4adpwbias,file="B2B2s4adpwbias.RData")

save(B2B2s4adpwmse,file="B2B2s4adpwmse.RData")

save(B2B3s4adalpha,file="B2B3s4adalpha.RData")

save(B2B3s4adaphse,file="B2B3s4adaphse.RData")

save(B2B3s4adaphbias,file="B2B3s4adaphbias.RData")

save(B2B3s4adaphmse,file="B2B3s4adaphmse.RData")

save(B2B3s4adpower,file="B2B3s4adpower.Rdata")

save(B2B3s4adpwse,file="B2B3s4adpwse.RData")

save(B2B3s4adpwbias,file="B2B3s4adpwbias.RData")

save(B2B3s4adpwmse,file="B2B3s4adpwmse.RData")

save(B2B4s4adalpha,file="B2B4s4adalpha.RData")

save(B2B4s4adaphse,file="B2B4s4adaphse.RData")

save(B2B4s4adaphbias,file="B2B4s4adaphbias.RData")

save(B2B4s4adaphmse,file="B2B4s4adaphmse.RData")

save(B2B4s4adpower,file="B2B4s4adpower.Rdata")

save(B2B4s4adpwse,file="B2B4s4adpwse.RData")

save(B2B4s4adpwbias,file="B2B4s4adpwbias.RData")

save(B2B4s4adpwmse,file="B2B4s4adpwmse.RData")

########################################################################
